# *Bothrops* venom variation drives niche-specific pharmacology through Ca^2+^ signalling and membrane damage

**DOI:** 10.3389/fphar.2026.1769550

**Published:** 2026-03-31

**Authors:** Lachlan A. Bourke, Julien Slagboom, Matthew A. Waller, G. Gregory Neely, Jeroen Kool, Fernanda C. Cardoso

**Affiliations:** 1 Institute for Molecular Bioscience, The University of Queensland, Brisbane, QLD, Australia; 2 School of the Environment, The University of Queensland, Brisbane, QLD, Australia; 3 Department of Chemistry and Pharmaceutical Sciences, Amsterdam Institute of Molecular and Life Sciences, Division of BioAnalytical Chemistry, Faculty of Science, Vrije Universiteit Amsterdam, Amsterdam, Netherlands; 4 Centre for Analytical Sciences Amsterdam (CASA), Amsterdam, Netherlands; 5 The Dr John and Anne Chong Laboratory for Functional Genomics, Charles Perkins Centre and School of Life and Environmental Sciences, University of Sydney, Sydney, NSW, Australia; 6 Centre for Innovation in Pain and Health Research, Faculty of Health, Medicine and Behavioural Sciences, The University of Queensland, Brisbane, QLD, Australia; 7 Centre for Motor Neuron Disease Research, Faculty of Health, Medicine and Behavioural Sciences, The University of Queensland, Brisbane, QLD, Australia

**Keywords:** bothrops, calcium, cytotoxic, ion channels, membrane, pantanal, venom

## Abstract

Snake venom activity exhibits evolutionary patterns within genera and varies according to ecological niche. By examining venoms from species inhabiting distinct environments, niche-specific functional differences can be uncovered. *Bothrops*, a diverse and medically important Neotropical pit viper genus, shows known clade-specific differences in coagulotoxicity. Here, we expanded this framework by assessing intra-clade variation in cellular activity across twelve *Bothrops* species using a high-content fluorescence assay that simultaneously measures ion channel responses and membrane cytotoxicity. Most venoms induced rapid membrane damage and cell lysis, whereas arboreal species lacked both activities, suggesting reduced selection for these activities. In contrast, the terrestrial *Bothrops mattogrossensis* and *Bothrops pauloensis,* which are sister species inhabiting the Pantanal wetlands and outskirts, respectively, displayed unique intracellular calcium-modulatory effects in the absence of membrane disruption. High throughput venomics revealed candidate toxin families underlying these calcium responses. Our findings demonstrate niche-specific diversification of venom bioactivity and highlight *Bothrops* venoms as promising sources of pharmacological agents.

## Introduction

Snake venom is a highly diverse mixture of bioactive compounds, mainly proteins and peptides, that aids in the incapacitation of prey and defence (Chan et al., 2016). Venom modulates many physiological processes, including blood coagulation and neuronal transmission, and variability in venom activity is documented both between and within species (Casewell et al., 2020; Gutiérrez et al., 2017). Research has shown that the main driver of snake venom variation, unsurprisingly, is diet (Barlow et al., 2009; Daltry et al., 1996; Jackson et al., 2016; Schaeffer et al., 2023; Lyons et al., 2020; Holding et al., 2021; Smith et al., 2023). Indeed, venom may evolve to target certain prey, and many venoms are prey-specific (Michálek et al., 2024). However, prey type alone is not sufficient to explain variation, as some studies have shown venom composition also correlates with environmental conditions (Smith et al., 2023; Zancolli et al., 2019). Furthermore, prey escape, rather than prey type, might account for venom variation (Sousa et al., 2018). Evidently, venom composition is determined by complex abiotic and biotic variables, and as such, snake venom holds a wealth of ecological and evolutionary information. In closely related species with vastly differing ecology, one might expect this ecological difference to be reflected in their venom.

*Bothrops* is a diverse group of pit-viper inhabiting numerous ecological niches—including islands, terrestrial habitats, and arboreal habitats (http://www.reptile-database.org/, accessed: 10 September 2025). Indeed, venom variation between species occupying different niches has been observed in *Bothrops*. We previously investigated the coagulotoxicity of venoms from nineteen *Bothrops* species, including three arboreal species (*Bothrops bilineatus*, *Bothrops oligolepis*, and *Bothrops taeniatus*) (Bourke et al., 2022). Both *B. bilineatus* and *B. taeniatus* lacked procoagulant activity and exhibited fibrinogen-degrading activity, contrary to most of the other terrestrial *Bothrops* species tested. The most basal *Bothrops, Bothrops pictus*, was strongly anticoagulant, indicating evolutionary differences between venoms (Bourke et al., 2022). Furthermore, intraspecific differences have been observed, with *Bothrops atrox* from floodplain habitats less haemorrhagic but more procoagulant than venoms from species in other habitats (Sousa et al., 2017). Current evidence points to venom variation in *Bothrops* being associated with ecological niche differences, although research on venom composition and bioactivity in many species is still limited, especially since many *Bothrops* remain understudied.

In this study, we investigated the bioactivity of venoms from twelve *Bothrops* species, including understudied species, spanning numerous niches (island-dwelling, arboreal and terrestrial). *Bothrops* venoms are highly active on cells and cause myotoxicity (Mamede et al., 2020) and general cytotoxicity (Marinho et al., 2024) in experimental studies, and myotoxicity-associated effects such as blistering, dermonecrosis, and myonecrosis in humans (Mora-Obando et al., 2023; Gutiérrez et al., 2009; Resiere et al., 2020). As myotoxic effects can be permanent—potentially causing limb amputations, ocular damage, muscle tissue loss, contractures, and chronic renal failure (Gutiérrez et al., 2017; Bittenbinder et al., 2024; Ferraz et al., 2019)—and since *Bothrops* are responsible for the majority of snake envenomations across Latin America (Chippaux, 2015; Costa et al., 2019; Dolab et al., 2014; da Silva et al., 2020; Otero-Patino, 2009), studying their cytotoxic venom effects is critical.

We evaluated venom activity on two human cell lines using a validated high-content bioassay that simultaneously measures intracellular calcium ([Ca^2+^]_i_) responses and DNA release (Kramer et al., 2024). These are key markers for ion channel/receptor modulation or cell-lytic activity, and with both types of bioactivity described in venoms of *Bothrops* species (Ferraz et al., 2019; Kramer et al., 2024; Bourke et al., 2025). Our results revealed remarkable differences between the venoms of arboreal and terrestrial *Bothrops*. In addition, an interesting and exclusive venom activity was observed in two terrestrial *Bothrops* species inhabiting the Pantanal region of Brazil and neighbouring countries Paraguay and Bolivia: *Bothrops pauloensis* and *Bothrops mattogrossensis*, spurring further investigations on their active components.

Accordingly, we expanded our investigations by combining venom nanofractionation, high-content bioassaying, and proteomic analysis to identify potential venom components in *B. pauloensis* and *B. mattogrossensis* exerting ion channel modulatory-like activity. This work is fundamental in characterising the cellular activity of venoms from numerous *Bothrops* species inhabiting distinct ecological niches and in revealing novel venom activities and components that expand the potential of venom-based pharmacological tools. We highlight that broader ecological and evolution-focused studies can aid in better understanding the pathophysiology leading to envenomation syndromes and advance venom-to-drug discovery by revealing venom activities with pharmacological potential.

## Methods

### Reagents and cell lines

All chemical reagents were from Sigma-Aldrich, otherwise as stated. All cell culture reagents were from Gibco ThermoFisher Scientific, otherwise as stated. Human embryonic kidney cell line (HEK293 and HEK293T) and neuroblastoma SHSY5Y cells were cultured following a similar protocol to previous work (Kramer et al., 2024). Briefly, HEK293 cells were cultured in low-glucose Dulbecco’s modified Eagle’s medium (DMEM), supplemented with 10% fetal bovine serum (FBS), and 100 units/mL penicillin and 100 μg/mL streptomycin. SHSY5Y cells were cultured in Roswell Park Memorial Institute (RPMI) medium supplemented with 15% FBS, 2 mM glutamine and 100 units/mL penicillin and 100 μg/mL streptomycin. All cultured cells were incubated at 37 °C in a humidified 5% CO_2_ incubator. Cells were subcultured approx. 2–3 times per week, following 70–80% confluency, using Dulbecco’s phosphate buffered saline (D-PBS) and 0.05% Trypsin/EDTA.

### Venom samples and preparation

A diverse set of twelve *Bothrops* species’ venom, encompassing terrestrial, arboreal, and island-dwelling species, was analysed in this study and described in Table 1. Most *Bothrops* tested are primarily terrestrial, two species are arboreal, and two species are island-dwelling.

**TABLE 1:** *Bothrops* venom samples and associated information.

Species	Pooled (n) or individual	Gender	Locality	Source, wild-caught or captive bred	Niche	Evolutionary clade
*B. alternatus*	ND	ND	South America	Latoxan	T	*B. alternatus*
*B. pictus*	Pooled (n = 7)	ND	Districts of Carabayllo and Comas, Peru	UNMSM, wild-caught	T	*B. alternatus*
*B. asper*	Pooled (n = 2)	M	Ecuador	Latoxan, captive-born	T	*B. atrox*
*B. atrox*	Pooled (n = 66)	M + F	French Guiana	Latoxan, captive-born and wild-caught	T*	*B. atrox*
*B. caribbaeus*	ND	ND	St. Lucia, West Indies	Kentucky reptile zoo, wild-caught	I*	*B. atrox*
*B. lanceolatus*	ND	ND	Martinque	Latoxan, imported	I*	*B. atrox*
*B. leucurus*	ND	ND	Brazil	Latoxan	T*	*B. atrox*
*B. diporus*	ND	ND	Unknown	ABL, imported	T	*B. neuwiedi*
*B. mattogrossensis*	ND	ND	Brazil	Latoxan	T	*B. neuwiedi*
*B. pauloensis*	ND	ND	Paraguay	Latoxan	T	*B. neuwiedi*
*B. oligolepis*	Pooled (n = 3)	ND	La Merced, Chanchamayo, Peru (Central rainforest region)	UNMSM, wild-caught	A	*B. taeniatus*
*B. taeniatus*	ND	ND	ND	ABL, imported	A	*B. taeniatus*

Niche information sourced from Martins et al. (2002); Martins et al. (2001); Campbell and Lamar (2004) and clade information from Carrasco et al. (2023). T, terrestrial, A, arboreal, I, island-dwelling. ABL, adaptive biotoxicology lab; UNMSM, armando yarleque, Universidad Nacional Mayor de San Marcos. 1. *Species may have some arboreal tendencies, especially juveniles. ND, not determined.

All snake venom samples were obtained in the lyophilised form for long-term storage. Prior to bioassays, venoms were weighed and stored at −20 °C. During bioassays, venom and synthetic melittin (Smartox Biotechnology, Saint Egrève, France) were reconstituted in assay buffer [PSS buffer with 0.1% Bovine Serum Albumin (BSA)]. Physiological saline solution (PSS) buffer was prepared with 140 mM NaCl, 11.5 mM glucose, 5.9 mM KCl, 1.4 mM MgCl_2_, 1.2 mM NaH_2_PO_4_, 5 mM NaHCO_3_, 1.8 mM CaCl_2_, and 10 mM HEPES (pH 7.4, dissolved in Milli-Q water).

### Fluorescence-imaging high-content duplex bioassay

A FLIPR Penta High-Throughput Cellular Screening System (Molecular Devices, CA, United States) was used to measure the bioactivity of venoms on SHSY5Y and HEK293 cells, as we previously described (Kramer et al., 2024). This bioassay is a duplex bioassay that simultaneously measures [Ca^2+^]_i_ responses and DNA release induced by venoms and other complex mixtures.

Cells were seeded in 384-well clear-bottom plates and incubated at 37 °C with 5% CO_2_ for 48 h prior to running the assay. On the assay day, a reagent plate was prepared containing a negative control (PSS buffer with 0.1% BSA), a positive control for DNA release (melittin, 50 µM), and venoms reconstituted in PSS buffer with 0.1% BSA (venoms are described in Table 1). Reconstituted venom was added into each well at a 20 mg/mL concentration and serially diluted into successive wells. The reagent plate was kept on ice during preparation, stored at 4 °C, and brought to room temperature 15 min prior to the assay run.

Once the reagent plate was prepared, culture media was pipetted out of the seeded cell plates and replaced with 20 µL of dye solution containing propidium iodide (PI) in Calcium 4 dye (Ca4 dye, Molecular Devices) reconstituted in PSS (PI = 50 µM and Ca4 dye = 1:10 dilution). PI binds to DNA and RNA, while Ca4 dye binds to intracellular calcium. Note that Ca4 dye contains an extracellular Ca^2+^ quencher. Plates were then incubated at 37 °C and 5% CO_2_ for 30 min prior to the assay run. FLIPR settings were as follows: reads before dispensing = 5, read interval = 2 s, read mode 1 (PI) recorded at 470–495 nm excitation and 565–625 nm emission, read mode 2 (Ca4 dye) recorded at 470–495 nm excitation and 515–575 nm emission. For experiments testing the efficacy of varespladib [Company: Chemietek, preparation: solubilised in 10% dimethyl sulfoxide (DMSO)] in reducing venom activity, varespladib at concentrations of 1.67 mM or 0.081 mM was incubated with venom. All wells were prepared to contain equal amounts of 10% DMSO.

### Flow cytometry

HEK293T cells were dissociated and aliquoted into 1.5 mL tubes at 5 × 10^6^ cells/tube. Cells were spun down, media were removed, and pellets were washed with 500 µL Dulbecco’s phosphate buffered saline (D-PBS; Sigma-Aldrich). Indo-1 AM (Invitrogen) was resuspended in dimethyl sulfoxide (DMSO; Sigma-Aldrich) to prepare a 100 µM stock solution, which was aliquoted and frozen for later use. The stock Indo-1 solution was thawed and diluted to 5 µM in D-PBS to prepare staining solution. Samples were resuspended in 250 µL Indo-1 staining solution and incubated at 37 °C and in the dark for 1 h. Samples were then spun down, staining solution was removed, and pellets were resuspended in 250 µL of D-PBS containing Live/Dead Fixable Near-IR Dead Cell stain for 633 or 635 nm excitation (Invitrogen), diluted 1:1,000. Samples were incubated in the dark at 37 °C for a further 30 min. Finally, samples were spun down, media was removed, and cells were washed once with PSS. Cells were left as pellets with media removed. All spins were carried out at 300 × g for 5 min.

Flow cytometry was then performed using the BD Biosciences Influx 7 L. Cell pellets were resuspended in either PSS only, PSS containing 10 µM ionomycin (Sigma-Aldrich), or *B. mattogrossensis* crude venom at 500 or 1,000 μg/mL, then passed through a 0.45 μM cell strainer into a 5 mL polypropylene tube and immediately run on the Influx 7 L. All solutions were kept on ice until 15 min prior to addition to a cell pellet. Samples were run for a total of 5 min at approximately 1 × 10^4^ events/s. Cells were gated based on: (i) FSC Perp vs. SSC, (ii) FSC-Perp vs. Trigger Pulse Width, (iii) SSC vs. SSC-W, (iv) FSC Perp vs. 750 nm fluorescence intensity, (v) 460 ± 50 nm fluorescence vs. 379 ± 28 nm fluorescence. Analysis was performed using FlowJo (v10). Indo-1 ratio was defined as 379 ± 28 nm fluorescence/460 ± 50 nm fluorescence. The gate for responding cells was set to the top ∼5% of cells in the PSS-only sample (Indo-1 ratio ≥0.7). Time-based analyses were performed by binning all events occurring in each 10 s interval, giving a total of 30 bins for 5 min of recording.

### Proteomics

We performed the proteomic analysis following the procedure outlined by us previously (Slagboom et al., 2023). Briefly, crude venom was fractionated using a gradient reversed-phase high-performance liquid chromatography (RP-HPLC) separation. Mobile phase A comprised 98% H_2_O, 2% acetonitrile (ACN), and 0.1% trifluoroacetic acid (TFA), while mobile phase B comprised 98% ACN, 2% H_2_O, and 0.1% TFA. Venom fractionation was performed over 50 min: mobile phase B increased linearly from 0% to 30% in 5 min, 30%–50% in 25 min, and 50%–90% in 4 min, followed by a 5-min isocratic elution at 90%, and final column equilibration for 10 min at starting conditions (1% B). The fractionation resolution was 12 s. Mass spectrometry and NanoLC-MS/MS were performed, and the Mascot database was used to identify proteins in each fraction. For further methodological details, see our previous work (Slagboom et al., 2023).

### Data analysis

ScreenWorks® Software (Molecular Devices) was used to extract raw trace data and kinetic reductions (area under the curve and Max-Min) from the FLIPR assays. The ratiometric (DNA response/[Ca^2+^]_i_ response) Max-Min was also extracted. Raw trace data were automatically sorted in R before further processing in GraphPad Prism 9.5.1 software (GraphPad Prism Inc., La Jolla, CA, United States). GraphPad Prism was used to analyse the data and generate graphs and statistical analysis, with *P* = 0.05 set as the significance threshold. To produce concentration-response curves, selected kinetic reduction data were normalised (100% defined as the maximum kinetic reduction value observed for the experiment). Following this, concentrations were log-transformed and then plotted using the nonlinear regression analysis with the concentration-response –stimulation, log (agonist vs. normalised response) – variable slope equation. EC_50_ values and associated standard deviation (SD) were calculated. EC_50_ values were calculated separately for independent FLIPR experiments (triplicate in each experiment, three independent experiments), then averaged, and SD determined. Although EC_50_ values are provided in Supplementary Material 1 for transparency, these estimates must be interpreted with caution, as most venoms did not reach 50% of the maximal effect within the concentration range tested. Under these conditions, the calculated EC_50_ values are relative (extrapolated) and are not suitable for comparing venoms. Therefore, to compare venoms, we instead used the normalised kinetic reduction values (AUC or Max-Min) at the highest or lowest venom concentration tested, depending on the analysis. These values were statistically compared using a Brown-Forsythe ANOVA and Welch’s ANOVA, with Dunnett’s T3 multiple comparisons test. Quantile-quantile (QQ) plots showed the data was approximately normal with some deviation; however, residual plots indicated violation of the homoscedasticity of variance assumption, thus a Brown-Forsythe ANOVA and Welch’s ANOVA test was used (Supplementary Material 3). Data are shown as mean ± SEM unless otherwise stated.

## Results

### [Ca^2+^]_i_ responses and DNA release induced by bothrops venoms

**FIGURE 1:**
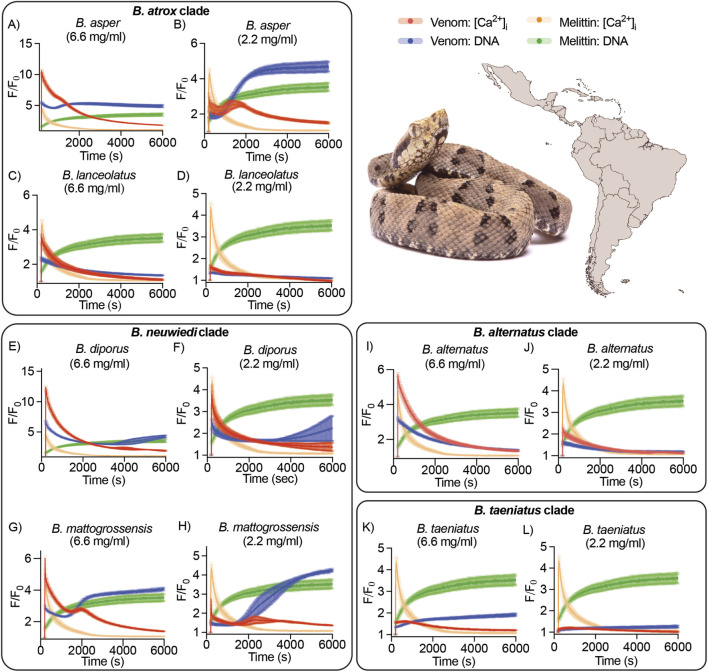
Bioactivity of *Bothrops* venoms on SHSY5Y cells. Fluorescence traces of propidium iodide (DNA response, blue traces) and Ca 4 dye ([Ca^2+^]_i_) response, red traces) in SHSY5Y cells exposed to venom from representative species of the clades *B. atrox* **(A–D)**, *B. neuwiedi* **(E–H)**, *B. alternatus* **(I,J)**, and *B. taeniatus* **(K,L)**. All graphs include the positive control melittin: fluorescence traces of cells exposed to melittin (DNA response, green traces; Ca 4 dye ([Ca^2+^]_i_) response, orange traces). Venom concentration tested is displayed under the species name. Data are represented by mean ± SEM, n = 9 wells of F/F_0_. Image: *B. atrox*, credit: ©Arthur. Map of America from Mexico to South America (BioRENDER).

We investigated the cellular activity of venoms from 12 species of *Bothrops* using a high-content bioassay that measures [Ca^2+^]_i_ responses and DNA release using fluorescence-imaging in a high-throughput platform. The bee venom toxin melittin, a pore-forming and lytic toxin, was used as a positive control for membrane damage. In both HEK293 and SHSY5Y cells, melittin (16.67 μM) induced a transient [Ca^2+^]_i_ response and sustained DNA response (Figure 1, Supplementary Material 2); this is characteristic of cytolytic activity. All venoms tested induced different responses to melittin. Within the *B. atrox* clade (*B. asper*, *B. atrox*, *B. carribaeus*, *B. lanceolatus*, and *B. leucurus*), two distinct activities were observed. Firstly, *B. asper* and *B. leucurus* induced an instantaneous increase in both [Ca^2+^]_i_ and DNA, with the [Ca^2+^]_i_ response decreasing over time and the DNA response increasing (Figures 1A,B, Supplementary Material 2). This is suggestive of cell lysis: both DNA and [Ca^2+^]_i_ are released from the cell, with subsequent termination of the [Ca^2+^]_i_ signal as it is exposed to an extracellular quencher. In comparision, *B. atrox* and the island dwelling *Bothrops* (*B. carribaeus* and *B. lanceolatus*) induced a similarly rapid increase in both [Ca^2+^]_i_ and DNA, followed by decreases in both responses over time (Figures 1C,D, Supplementary Material 2).

Venoms in the *B. neuwiedi* clade (*B.* *diporus*, *B. mattogrossensis*, and *B. pauloensis*) had similar lytic activity to *B. asper* and *B. leucurus*, inducing a transient [Ca^2+^]_i_ increase and a sustained DNA increase (Figures 1E–H, Supplementary Material 2). Interestingly [Ca^2+^]_i_ responses appeared to form a sharp peak in *B. mattogrossensis* and *B. pauloensis* venoms, which was absent in the other *Bothrops* species investigated. The *B. alternatus* clade (*B. alternatus* and *B. pictus*) showed rapid increase in both [Ca^2+^]_i_ and DNA, followed by decreases in both responses over time for *B. alternatus* venom. On the other hand, *B. pictus* venom led to a decrease in the Ca^2+^ response and an increase in the DNA response over time (Figures 1I,J, Supplementary Material 2). Lastly, the arboreal *B. taeniatus* clade (*B. taeniatus* and *B. oligolepis*) had minimal effects on both cell types, with low [Ca^2+^]_i_ and DNA responses (Figures 1K,L, Supplementary Material 2).

### Potency of bothrops venoms in modulating [Ca^2+^]_i_ responses and membrane damage

**FIGURE 2:**
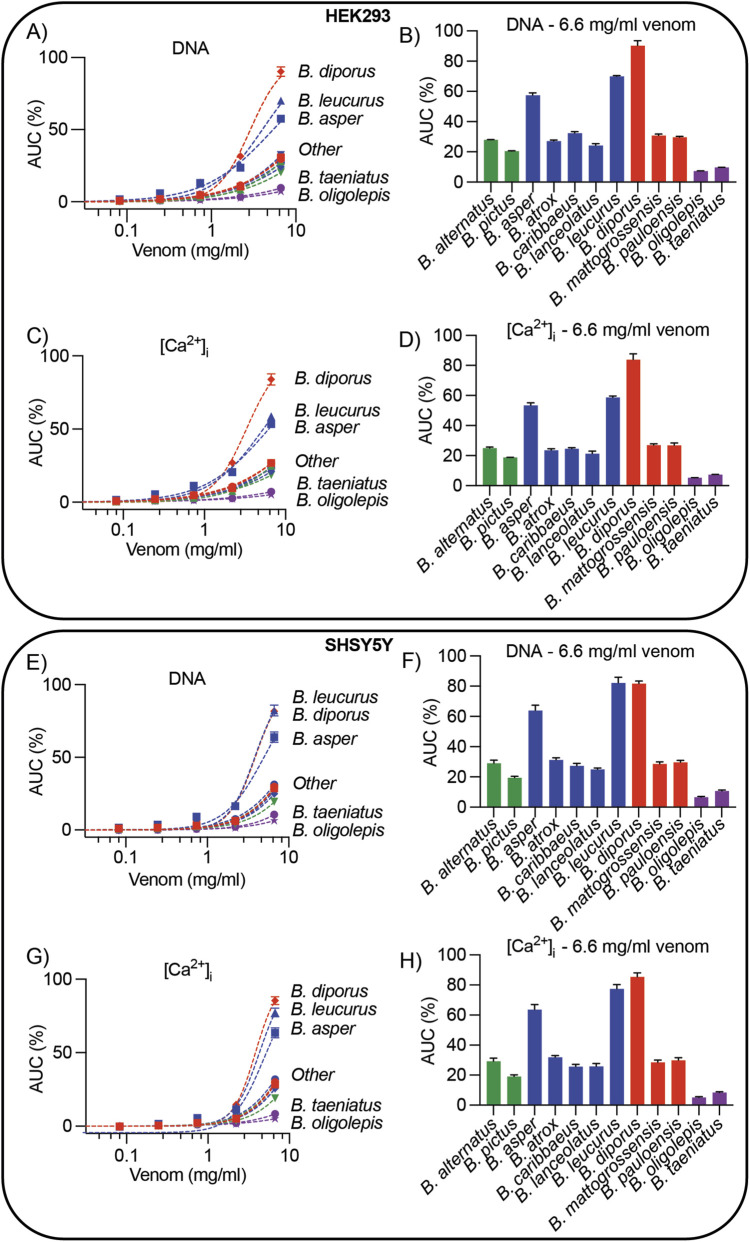
Potency of the bioactivity of *Bothrops* venoms on HEK293 and SHSY5Y cells. **(A,C,E,G)** Concentration-response curves of twelve *Bothrops* venoms measured using Propidium iodide [**(A,E)**, DNA response] or Ca 4 dye (**(C,G)** [Ca^2+^]_i_ response) on HEK293 cells **(A,C)** or SHSY5Y cells **(E,G)**. The response measured was the area under the curve (AUC, y-axis) across seven venom concentrations (mg/ml, x-axis). **(B,D,F,H)** Normalised AUC values at the highest venom concentration (6.66 mg/mL) were calculated for the DNA response **(B,F)** or [Ca^2+^]_i_ response **(D,H)**. Venoms are coloured according to evolutinary clade: *B. alternatus* clade (green), *B. atrox* clade (blue), *B. neuwiedi* clade (red), *B. taeniatus* clade (purple). All data is mean ± SEM, n = 3 independent experiments performed in triplicates and measured over approx. 900 s. Venom response measured for 900 s. Statistics are presented in Supplementary Material 3.

Concentration-response curves and EC_50_ values were calculated to compare the relative potency between venoms (Figure 2, Supplementary Material 1). However, most venoms did not reach 50% of the maximal effect within the concentration range tested, thus EC_50_ values could not be used to compare venoms. Instead, the normalised AUC value at the highest venom concentration was plotted alongside concenration response curves and used to compare venoms (Figures 2B,D,F,H). Unlike Figure 1, the following data were measured over approximately 900 s (15 min), focusing on the initial responses induced by each venom. A major clade-specific difference was observed for arboreal venoms (the *B. taeniatus* clade). Both arboreal *Bothrops* (*B. taeniatus* and *B. oligolepis*) were significantly less potent than all other venoms, with normalised AUC values at the 6.6 mg/mL venom concentration of 9.642 ± 0.144 and 7.337 ± 0.052, respectively, for DNA response, and 7.290 ± 0.149 and 5.222 ± 0.064, respectively, for [Ca^2+^]_i_ measurements (Figure 2; Supplementary Material 3). *Bothrops diporus* was the most potent venom on HEK293 cells, with a significantly higher normalised AUC value than all other venoms for both DNA and [Ca^2+^]_i_ measurements. The next most potent venoms were *B. asper* and *B. leucurus*. Patterns seen in HEK293 cells were also observed in SHSY5Y cells, except *B. leucurus* was comparable in potency to *B. diporus* (Figure 2; Supplementary Material 3).

### In-depth characterization of *Bothrops mattogrossensis* and *Bothrops pauloensis* crude venoms

**FIGURE 3:**
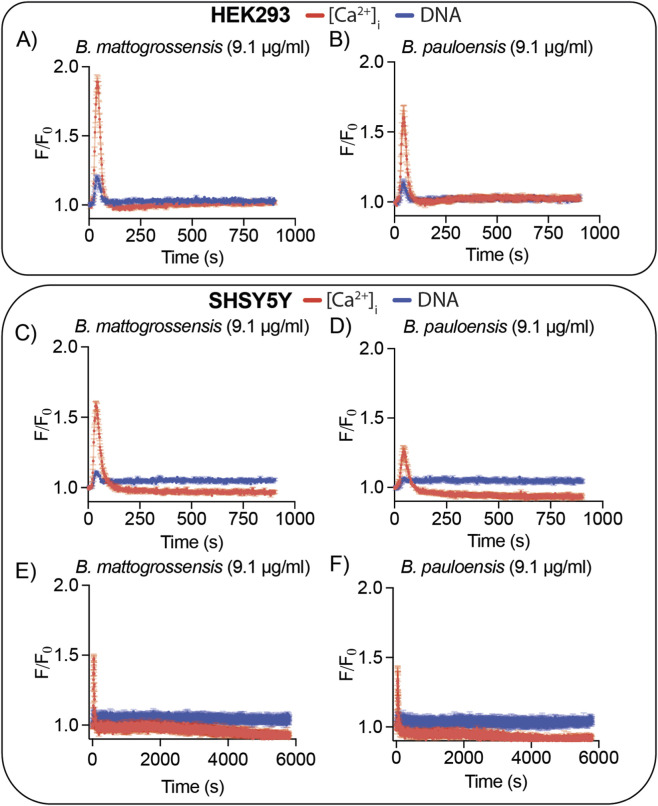
Bioactivity of *B. mattogrossensis* and *B. pauloensis* venoms on HEK293 and SHSY5Y cells highlighting their predominant [Ca^2+^]_i_ response. **(A–F)** Fluorescence traces of propidium iodide (DNA response, blue traces) and Ca 4 dye ([Ca^2+^]_i_ response, red traces) in cells exposed to venom. **(A,B)** Recordings of HEK293 cells exposed to 9.1 μg/mL venom of *B. mattogrossensis* **(A)** or *B. pauloensis* **(B)** for approx. 900 s. **(C–F)** Recordings of SHSY5Y cells exposed to venom of *B. mattogrossensis* **(C,E)** or *B. pauloensis* **(D,F)** for approx. 900 s **(C,D)** or 5,800 s **(E,F)**. Data are mean ± SEM, n = 9 wells **(A–D)** or n = 3 wells **(E,F)**.

Both *B. mattogrossensis* and *B. pauloensis* produced an early and rapid [Ca^2+^]_i_ spike in HEK293 and SHSY5Y cells (Figure 3). Unlike other venoms, testing of lower concentrations of venom revealed this rapid [Ca^2+^]_i_ spike persisted without a DNA response (Figure 3). In both HEK293 and SHSY5Y cells, *B. mattogrossensis* induced a larger [Ca^2+^]_i_ response than *B. pauloensis.* Long assay runs of approximately 6,000 s on SHY5Y revealed no later-stage cell lysis as characterized by DNA release (Figures 3E–F).

**FIGURE 4:**
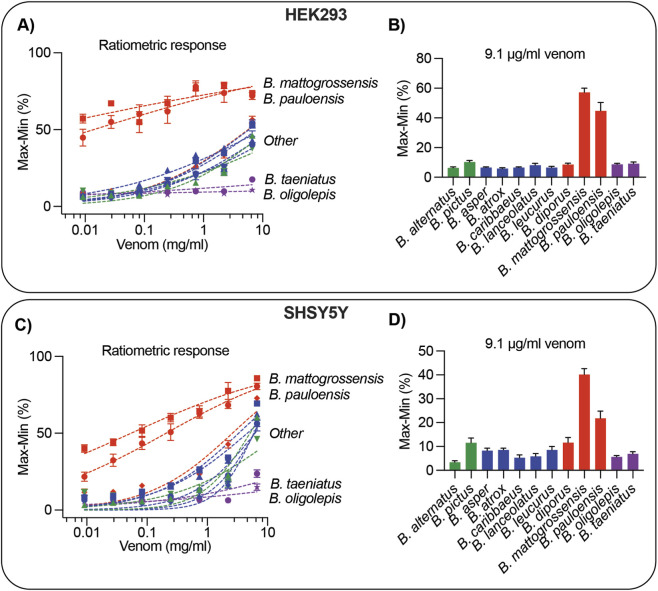
Ratiometry of the [Ca^2+^]_i_/DNA responses induced by *B. mattogrossensis* and *B. pauloensis* venoms bioactivity on HEK293 and SHSY5Y cells. **(A,C)** Concentration-response curves of twelve *Bothrops* venoms measured using propidium iodide (DNA response) and Ca 4 dye ([Ca^2+^]_i_ response) on HEK293 **(A)** or SHSY5Y **(C)** cells. The response measured was the Max-Min of the ratiometric value ([Ca^2+^]_i_ response/DNA response) (y-axis) across seven venom concentrations (mg/ml, x-axis). **(B,D)** Normalised Max-Min values at the lowest concentration of venom (9.1 μg/mL) for HEK293 **(B)** or SHSY5Y **(D)**. Venoms are coloured according to evolutinary clade: *B. alternatus* clade (green), *B. atrox* clade (blue), *B. neuwiedi* clade (red), *B. taeniatus* clade (purple). All data are mean ± SEM of n = 3 independent experiments performed in triplicates and measured over approx. 900 s. Statistics are presented in Supplementary Material 3.

Ratiometric analyses further highlighted the remarkable high potency of *B. mattogrossensis* and *B. pauloensis* venoms in producing a rapid [Ca^2+^]_i_ spike without DNA response (Figure 4). As above, normalised kinetic-reduction values were used to compare venoms, since some venoms did not reach 50% of the maximal effect. *Bothrops mattogrossensis* was more potent than *B. pauloensis* in HEK293 cells, with normalised Max-Min values at the lowest venom concentration (0.009 mg/mL) of 57.206 ± 2.848 and 44.774 ± 5.578, respectively; however, this difference was not significant (Figure 4; Supplementary Material 2). Conversely, *B. mattogrossensis* was significantly more potent than *B. pauloensis* in SHSY5Y cells, with normalised Max-Min values at the lowest venom concentration (0.009 mg/mL) of 40.129 ± 2.491 and 21.791 ± 3.015, respectively (Figure 4; Supplementary Material 2). Interestingly, both venoms had higher Max-Min values on HEK293 cells compared to SHSY5Y cells.

**FIGURE 5:**
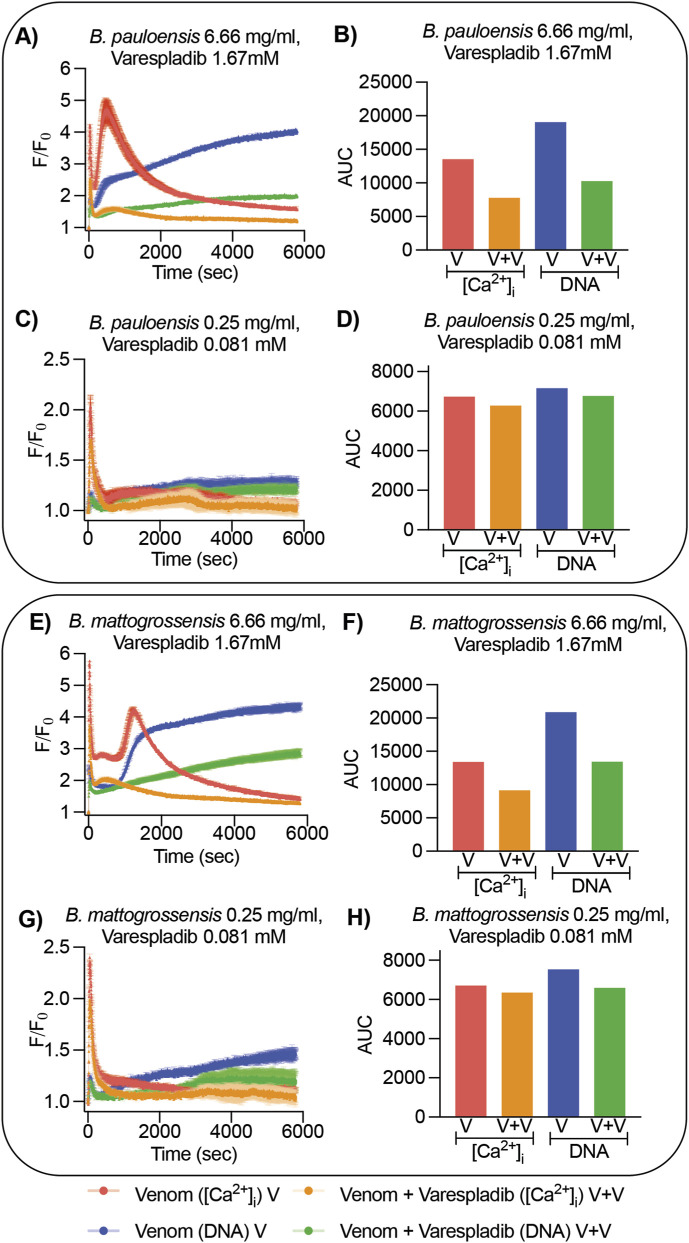
Bioactivity of *B. mattogrossensis* and *B. pauloensis* venoms on SHSY5Y cells in the presence of the PLA_2_ inhibitor varespladib. Propidium iodide (DNA response, blue and green traces) and Ca 4 dye ([Ca^2+^]_i_ response, red and orange traces) in SHSY5Y cells exposed to *B. pauloensis* and *B. mattogrossensis* venom with and without varespladib **(A,C,E,G)** Fluorescence trace recordings of *B. pauloensis* and *B. mattogrossensis* venom at concentration 6.66 or 0.25 mg/mL in the absence (red and blue) or presence (orange and green) of varespladib at 1.67 or 0.081 mM concentration. These results demonstrate that the rapid increases in intracellular Ca^2+^ concentration ([Ca^2+^]_i_) were preserved in the presence of varespladib, whereas the DNA-associated responses were markedly attenuated. **(B,D,F,H)** Area under the curve (AUC) calculated from the fluorescence traces of the entire recording in the presence of *B. pauloensis* and *B. mattogrossensis* venom at concentration of 6.66 or 0.25 mg/mL in the absence (red and blue) or presence (orange and green) of varespladib at 1.67 or 0.081 mM concentration. Data are represented by the mean (n = 2 wells per condition tested).

To determine whether snake venom PLA_2_s are involved in the [Ca^2+^]_i_ activity, *B. mattogrossensis* and *B. pauloensis* venoms were incubated with the PLA_2_ inhibitor varespladib before testing (Figure 5). In these experiments, we used 250 μg of varespladib per 1 mg of venom (final volume 1 mL), corresponding to a 0.25:1 (w/w) inhibitor:venom ratio, consistent with previously published protocols investigating the neutralization of PLA_2_-dependent hemotoxic activity (Zdenek et al., 2020; Youngman et al., 2020; Bourke et al., 2024). At the highest concentration of venom (6.66 mg/mL), where lytic activity is observed, varespladib reduced the AUC of both [Ca^2+^]_i_ and DNA responses, indicating PLA_2_s play a role in this activity. In contrast, at low concentration of venom (0.25 mg/mL), where the characteristic [Ca^2+^]_i_ spike occurs, varespladib did not reduce this response, suggesting this specific activity is not induced by varespladib-sensitive snake venom PLA_2_s.

We further investigated the bioactivity of *B. mattogrossensis* using cell-death staining coupled to [Ca^2+^]_i_ measurements in flow cytometry. Although significant effort was made to detect [Ca^2+^]_i_ responses independent of cell death, the rapid transient nature of the [Ca^2+^]_i_ responses (approximately 1 s duration) prevented detection using this methodological approach. However, we confirmed that *B. mattogrossensis* venom at 500 and 1,000 μg/mL induced significant cell death in HEK293 T cells in a concentration-dependent manner (Supplementary Figure S2). These results further confirm our findings from the high-content assay at high venom concentrations.

### High throughput nanovenomics and bioassaying of *Bothrops mattogrossensis* and *Bothrops pauloensis* venoms

**FIGURE 6:**
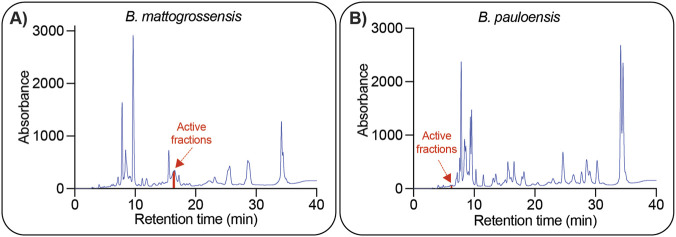
Chromatogram profiles of *B. mattogrossensis* **(A)** and *B. pauloensis* **(B)** venoms obtained from the RP-HPLC venom nanofractionation. The three active fractions of each venom identified from the high throughput screening assays (16:21 to 16:42 min for *B. mattogrossensis* in **(A)**; 6:13 to 6:37 min for *B. pauloensis* in **(B)** are indicated by red arrows and their chromatogram area coloured in red.

Upon RP-HPLC nanofractionation, *B. mattogrossensis* and *B. pauloensis* had chromatogram profiles with some notable differences (Figure 6). The number of peaks and heights of these peaks differed considerably between venoms, suggesting differences in the quantitative distribution of toxins and isoforms. *Bothrops pauloensis* also showed a more complex profile between 20 and 30 min retention time, with more well-defined peaks observed (Figure 6B).

**FIGURE 7:**
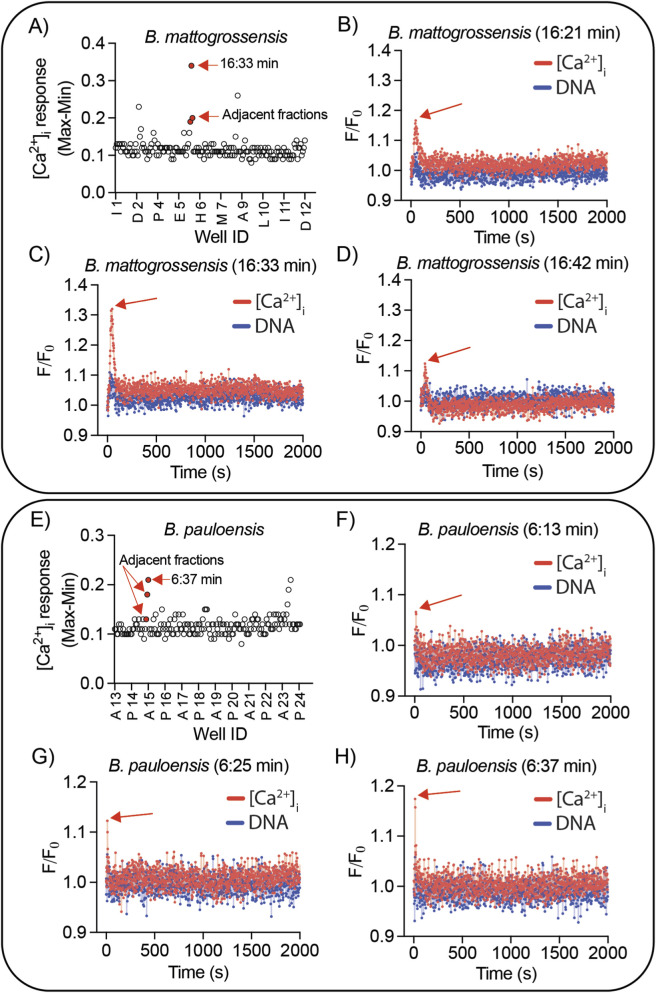
High throughput screening assays showing the active fractions identified in SHSY5Y cells exposed to fractions from *B. mattogrossensis* venom (top panel, A-D) and *B. pauloensis* venom (bottom panel, E-H) **(A,E)** Max-Min values extracted from the fluorescent traces for the [Ca^2+^]_i_ responses for all fractions tested. The fractions with the largest [Ca^2+^]_i_ responses **(A)** 16.33 min for *B. mattogrossensis* and **(E)** 6.37 min for *B. pauloensis*, are alongside adjacent fractions whose traces also showed a response **(B,C,D,F,G,H)** Fluorescence traces of Ca 4 dye ([Ca^2+^]_i_ response, red traces) and propidium iodide (DNA response, blue traces) in SHSY5Y cells exposed to the identified active fractions. Arrows highlight the [Ca^2+^]_i_ responses in active fractions.

RP-HPLC nanofractions of *B. mattogrossensis* and *B. pauloensis* venom were tested for their ability to induce [Ca^2+^]_i_ and DNA responses in SHSY5Y cells. Analysis of bioassay data revealed a strong [Ca^2+^]_i_ response induced by the fraction eluted at 16:33 min for *B. mattogrossensis* (Figures 6A, 7A) and by fraction eluted at 6:37 min for *B. pauloensis* (Figures 6B, 7E)*.* Fluorescence traces of these and adjacent fractions revealed rapid [Ca^2+^]_i_ without DNA release (Figures 7B–D, F–H), consistent with the activity of crude venom (Figure 3).

Interestingly, the identified active fractions differed in elution times on the chromatograms between the two venoms (Figure 6). The active fractions of *B. mattogrossensis* eluted at approximately 16–17 min, whereas the active fractions of *B. pauloensis* are eluted at approximately 6 min. Other RP-HPLC nanofractions showed lower-intensity [Ca^2+^]_i_ responses or were no-consecutive fractions; therefore, they were not selected for discussion but warrant future investigation. Fractions from anion- and cation-exchange chromatography were also tested; however, due to the presence of salts, it was not possible to distinguish between venom-induced activity or salt-induced effects.

### Identification of [Ca^2+^] modulators in *Bothrops mattogrossensis* and *Bothrops pauloensis* venoms

**FIGURE 8:**
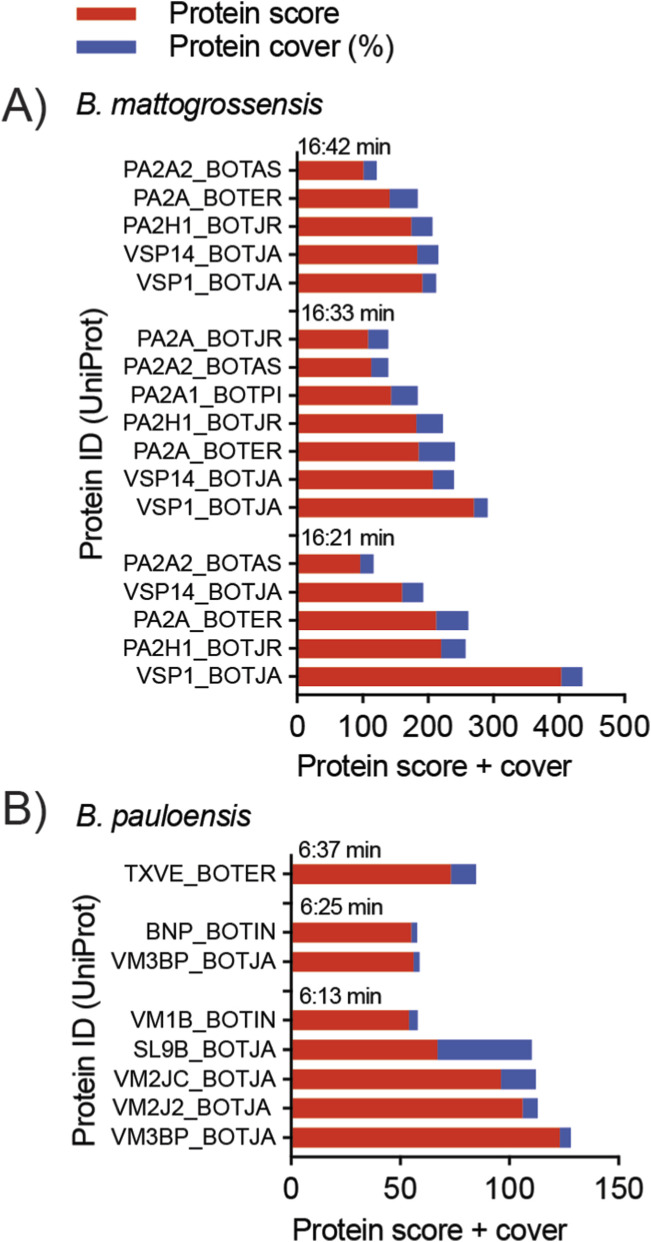
Identification of active toxin candidates in *B. mattogrossensis* and *B. pauloensis* venoms inducing exclusive [Ca^2+^]_i_ responses. **(A)** Snake toxins identified by proteomic analysis of active fractions 16:21, 16:33 and 16:42 min obtained from the fractionated venom of *B. mattogrossensis*. Highest probability combining the protein score (red) and the protein coverage (blue) is represented by bar graphs. These results pointed to a snake venom serine protease UniProt ID VSP1_BOJTA as the closest homolog to the active component in *B. mattogrossensis* venom. **(B)** Snake toxins identified by proteomic analysis of active fractions 6:13, 6:25 and 6:37 min obtained from the fractionated venom of *B. pauloensis*. Highest probability combining the protein score (red) and the protein coverage (blue) also pointed to a snake venom serine protease UniProt ID VM3BP_BOJTA as the closest homolog to the active component in *B. pauloensis* venom. Panels A and B were constructed with data described in Tables 2, 3.

Potential toxins inducing [Ca^2+^]_i_ response by *B. mattogrossensis* and *B. pauloensis* venoms were identified from proteomics data using Mascot database searching. To increase confidence in the results, toxins originating from *Bothrops* species with a protein score >50 and protein coverage >20% were selected, as per cut-offs used previously (Slagboom et al., 2020). Multiple potential toxins were identified per fraction (Figure 8; Tables 2, 3). Active fractions of *B. mattogrossensis* eluted between 16:21 to 16:42 min were identified as containing serine protease and/or PLA_2_ toxins (Figure 8A; Table 2). Similar proteins were detected in each fraction, suggesting that the same toxin or toxin family is responsible for the observed activity across these fractions.

**TABLE 2:** Mascot database search results of probable proteins in each active fraction of *Bothrops mattogrossensis* venom. Only proteins originating from *Bothrops* with protein score >50 and protein cover >20% are shown.

Retention time (min)	Protein ID (UniProt)	Protein score	Protein cover (%)	Protein family	Protein name, species
16:21	VSP1_BOTJA	403	32.9	SVSP	PA-BJ, *B. jararaca*
	PA2H1_BOTJR	220	37.2	Basic PLA_2_	BthTX-I, *B. jararacussu*
	PA2A_BOTER	212	49.3	Acidic PLA_2_	BE-I-PLA_2_, *B. erythromelas*
	VSP14_BOTJA	160	32.6	SVSP	HS114, *B. jararaca*
	PA2A2_BOTAS	96	20.2	Acidic PLA_2_	BaspPLA_2_-II, *B. asper*
16:33	VSP1_BOTJA	270	20.7	SVSP	PA-BJ, *B. jararaca*
	VSP14_BOTJA	207	32.6	SVSP	HS114, *B. jararaca*
	PA2A_BOTER	185	55.8	Acidic PLA_2_	BE-I-PLA_2_, *B. erythromelas*
	PA2H1_BOTJR	182	40.9	Basic PLA_2_	BthTX-I, *B. jararacussu*
	PA2A1_BOTPI	143	41	Acidic PLA_2_	BpirPLA_2_-I, *B. pirajai*
	PA2A2_BOTAS	113	25.8	Acidic PLA_2_	BaspPLA_2_-II, *B. asper*
	PA2A_BOTJR	108	31.2	Acidic PLA_2_	BthA-1, *B. jararacussu*
16:42	VSP1_BOTJA	191	20.7	SVSP	PA-BJ, *B. jararaca*
	VSP14_BOTJA	183	32.2	SVSP	HS114, *B. jararaca*
	PA2H1_BOTJR	174	32.1	Basic PLA_2_	BthTX-I, *B. jararacussu*
	PA2A_BOTER	141	42.8	Acidic PLA_2_	BE-I-PLA_2_, *B. erythromelas*
	PA2A2_BOTAS	101	20.2	Acidic PLA_2_	BaspPLA_2_-II, *B. asper*

*Abbreviations:* BthTX-I, Bothropstoxin-I; PLA_2_ = phospholipase A2; SVSP, snake venom serine protease.

**TABLE 3:** Mascot database search results of probable proteins in each active fraction of *Bothrops pauloensis* venom. Only protein originating from *Bothrops* with protein score >50 are shown. Only one identified protein had protein cover >20% (SL9B_BOTJA).

Retention time (min)	Protein ID (UniProt)	Protein score	Protein cover (%)	Protein family	Protein name, species
6:13	VM3BP_BOTJA	123	5.1	SVMP/disintegrin	bothropasin, *B. jararaca*
	VM2J2_BOTJA	106	6.9	SVMP/disintegrin	Jararafibrase II, *B. jararaca*
	VM2JC_BOTJA	96	16.1	SVMP/disintegrin	Jararacin, *B. jararaca*
	SL9B_BOTJA	67	43.3	Snaclec coagulation factor	IX/X-bp subunit B, *B. jararaca*
	VM1B_BOTIN	54	3.9	SVMP	BITM02A, *B. insularis*
6:25	VM3BP_BOTJA	56	2.8	SVMP/disintegrin	bothropasin, *B. jararaca*
	BNP_BOTIN	55	2.6	BP and C-type natriuretic peptide	BPP-CNP, *B. insularis*
6:37	TXVE_BOTER	73	11.6	Snake venom VEGF toxin	svVEGF, *B. erythromelas*

*Abbreviations:* BP, Bradykinin-potentiating; Snaclec, snake C-type lectin; SVMP, snake venom metalloproteinase protease; VEGF, vascular endothelial growth factor.

Only one protein that met the protein score and protein-coverage cut-offs was identified for *B. pauloensis* in fractions eluted at retention time 06:13 to 06:37 min—a C-type lection coagulation factor IX/X-binding protein subunit B (protein score = 67, protein coverage = 43.3%) (Figure 8B; Table 3). Most proteins identified had low protein coverage (<5.0%), so Table 3 highlights all *Bothrops* proteins with protein score >50 regardless of coverage. Low protein coverage indicates limited confidence in the identifications, making the results for *B. pauloensis* likely inconclusive.

## Discussion

This study aimed to characterise the cytotoxic activity of twelve *Bothrops* species venoms using a novel methodology applying high-content bioassaying developed by us (Kramer et al., 2024). By testing a diverse set of venoms that included representatives across the *Bothrops* phylogeny and numerous ecological niches (terrestrial, arboreal, and island-dwelling), we aimed to unravel ecological and evolutionary venom patterns. Our results revealed significant differences in venom activity between the *Bothrops* species. At high concentrations, most species’ venoms lysed cells or exhibited potential pore-forming activity; however, we observed interesting exceptions. Firstly, *B. oligolepis* and *B. taeniatus* had limited cytolytic activity, which is possibly associated with their arboreal lifestyle. Additionally, unique venom responses were observed for *B. mattogrossensis* and *B. pauloensis*, prompting expansion of the study to include proteomic analyses and follow-up bioassays on this group.

The methodological approach applied here provided substantial insights into *Bothrops* venoms’ cellular activity. At high venom concentrations, five species tested (*B. asper*, *B. leucurus*, *B. mattogrossensis*, *B. pauloensis*, and *B. diporus*) induced transient increases in [Ca^2+^]_i_ and sustained DNA release (Figure 1), indicative of rapid cell lysis (Kramer et al., 2024). This aligns with previous studies showing many *Bothrops* are cytotoxic *in vitro* (Marinho et al., 2024; Guerra-Duarte et al., 2015), and match clinical pathologies of *Bothrops* envenomations, including blistering, dermonecrosis, and myonecrosis (Mamede et al., 2020; Mora-Obando et al., 2023; Gutiérrez et al., 2009). Beyond cell lysis, some venoms (*B. atrox*, *Bothrops caribbaeus*, *B*. *lanceolatus*, *B. alternatus*, and *B. pictus*) induced immediate DNA and [Ca^2+^]_i_ responses followed by a decline of both signals (Figure 1), suggestive of pore formation as previously observed by us (Kramer et al., 2024). Apoptotic pathways may also be activated by these venoms, inducing cell death without rapid lysis during the experiment timeframe. Although beyond the scope of this study, this activity warrants further investigation. Overall, few clade-specific differences were found, aside from the consistent weak cytotoxicity of arboreal species.

Arboreal *taeniatus* group (*B. taeniatus* and *B. oligolepis*) exhibited no cytolytic activity (Figures 1, 2). This finding is notable because these were the only fully arboreal species tested, with all others primarily terrestrial. A reasonable hypothesis is that strong cytotoxic activity is not selected for in arboreal habitats. Our previous work showed that arboreal *B. bilineatus* and *B. taeniatus* venoms lacked coagulant activity, yet the arboreal *B. oligolepis* retained it (Bourke et al., 2022). Why *B. oligolepis* differ in coagulotoxicity remains unclear, and further natural history and ecological studies are needed to uncover this group’s venom variation. Contradicting our results, previous work has showed *B. taeniatus* has moderate myotoxic activity in avian muscle preparations (Romero-Vargas et al., 2014), although this method is vastly different from our assay conditions. Additionally, both *B. taeniatus* and *B. oligolepis* were cytotoxic across three tested cell lines after 24-h incubations (Guerra-Duarte et al., 2015); unlike our experiment which immediately measured cell activity after venom application.

Some studies suggest arboreal habitats may favour fast-acting venom due to high prey-escape potential in the canopy (Bourke et al., 2022). Indeed, neurotoxic activity has been reported for *B. bilineatus* and *B. taeniatus* (Romero-Vargas et al., 2014; Floriano et al., 2013; Rodrigues-Simioni et al., 2011), although no such work exists for *B. oligolepis*. In addition, a study found that isolated PLA_2_ toxin Bbil-TX from *B. bilineatus* had no cytotoxicity in skeletal muscle nor systemic myotoxic effects, but induced local myotoxicity in mice (Corasolla Carregari et al., 2013). Another study showed that the crude venom of *B. bilineatus smaragdinus* induced [Ca^2+^]_i_ increase in SK-N-SH neuroblastoma cell line and mouse triangularis sterni nerve-muscle preparations (Floriano et al., 2015). The contradictory results of *B. bilineatus* are evidence of the complex nature of venom evolution; however, our assays provided some evidence for unique venoms among this group by clustering the arboreal species, warranting further investigations of these venom bioactivities and compositions.

One of the most interesting results in our study were the [Ca^2+^]_i_ transients observed at low concentrations of *B. mattogrossensis* and *B. pauloensis* venom (Figures 3, 4). This activity is similar to the activity previously observed on the same assay for *Bothrops jararaca* and the Middle Eastern/Asian viper *Echis carinatus* (Kramer et al., 2024). Interestingly, *B. diporus*, the sister species of *B. mattogrossensis* and *B. pauloensis*, did not have this activity (Figure 3). One possible explanation is the unique niche occupied by *B. mattogrossensis* and in part by *B. pauloensis*, the Pantanal region, which is the world’s largest tropical wetland and flooded grassland area, in which prey escape might play a significant role in venom adaptation (Nogueira et al., 2019). While *Bothrops matttogrossensis* occupies the Pantanal, *B. pauloensis* predominantly inhabits the Cerrado and transitional areas meeting the Pantanal. This overlap may explain why *B. pauloensis* venom can also induce a similar activity. Although *B. diporus* also belongs to the *B. neuwiedi* clade (along with *B. mattogrossensis* and *B. pauloensis*), it differed in the cellular responses induced by its venom, suggesting this activity is a niche-specific adaptation rather than a shared trait of the *B. neuwiedi* clade. Further supporting our findings, studies of the venom of *Bothrops moojeni* also inhabiting the Pantanal and Cerrado regions of Brazil have shown its venom induces rapid intracellular calcium responses in the absence of membrane cytotoxicity (Zhang et al., 2017). Future venom and natural history studies are needed to test this hypothesis, including in other species within the *neuwiedi* clade. Since Ca^2+^ is vital second messenger signalling molecule regulated by numerous ion channels and receptors, this new activity observed in *Bothrops* inhabiting the Pantanal region and surrounds is suggestive of ion channel and/or receptor modulation. The [Ca^2+^]_i_ screen are a key first step in searching for venoms with biomedical drug properties, as shown in many studies (Cardoso et al., 2023; Cardoso et al., 2022; Cardoso et al., 2015; Cardoso et al., 2021) – if a toxin activates a specific pathway, it can be harnessed for therapeutic purposes. In fact, American pit viper venoms with Ca^2+^ activity have been shown to modulate a variety of ion channels and receptors (Bourke et al., 2025).

To discover the toxins involved in this activity, varespladib was used to investigate the role of PLA_2_s. Varespladib did not inhibit the [Ca^2+^]_i_ spike induced by the venoms (Figure 5), indicating varespladib-sensitive PLA_2_s are not responsible for this activity. Varespladib did, however, reduce the lytic activity seen at high concentrations of venoms. This corroborates previous work as PLA_2_ myotoxins are abundant in *Bothrops* venoms (Mora-Obando et al., 2023; Lomonte, 2023; Monteiro et al., 2020). Venom fractionation coupled to bioassays provided further insights into the toxins responsible for the [Ca^2+^]_i_ spike activity (Figures 6–8; Tables 2, 3). Active fractions from each venom were discovered; however, unexpectedly, proteomic results revealed different probable proteins for each venom. The most likely candidate for *B. mattogrossensis*, based on protein score, is a snake venom serine protease (SVSP) similar to the platelet-aggregating endopeptidase of *B. jararaca* (PA-BJ, Uniprot ID VSP1_BOTJA). Snake venom serine proteases are potent enzymes acting on the blood coagulation cascade and can act as procoagulant or anticoagulant agents, causing significant disruption of blood coagulation during envenomation (Ferraz et al., 2019). PA-BJ has been shown to release Ca^2+^ from intracellular stores in platelets and lung fibroblasts, through mediation of Protease-activated receptor one and 4 (PAR1 and PAR4) (Santos et al., 2000). Other potential proteins include the PLA_2_ BthTX-I, which has also been shown to modulate [Ca^2+^]_i_ (Cintra-Francischinelli et al., 2009; Rodrigues-Simioni et al., 1995). Interestingly, the Lys49 PLA_2_ BomoTx isolated from *B. moojeni* increases [Ca^2+^]_i_ mediated by purinergic receptors (Zhang et al., 2017). The fact that these probable proteins have known [Ca^2+^]_i_ modulatory activity suggests identification of the correct active fractions responsible for the observed crude venom activity.

On the other hand, identification of potential proteins from *B. pauloensis* venom was less conclusive. Most had low protein coverage (<5%), which gives limited confidence in the results. One candidate had a protein cover of 43.3% – a C-type lectin from *B. jararaca* (Uniprot ID SL9B_BOTJA) (Figure 8B; Table 3). A previous study showed this toxin has [Ca^2+^]_i_ activity; the C-type lectin BIL from *B*. *leucurus* has been shown to increase [Ca^2+^]_i_ in B16-F10 melanoma cell line (Aranda-Souza et al., 2014). Other potential candidates are snake venom metalloproteinases (e.g., UniProt ID VM3BP_BOTJA) which are enzymes that digest the extracellular matrix and cause significant tissue damage during envenomation, as well as function as anticoagulation factors causing haemorrhage (Ferraz et al., 2019). The low absorbance of fractions on RP-HPLC chromatogram suggests the cellular response observed in Figure 7 could be derived from a highly potent toxin. This finding highlights the challenges in identifying active components in venoms, particularly if these are in low abundance or are novel proteins or peptides not represented in databases. Future work should explore this snake venom, deepening in the results we found in this study, for example, using other fractionation techniques to find the active component(s). Our results provide evidence for *B. mattogrossensis* and *B. pauloensis* venom as a potential source of new pharmacological agents, following drug-development-focused studies. These would involve an extensive array of assays to find the mechanism of action of each venom component identified. The future research this study will enable on these venoms is particularly exciting.

In conclusion, this study applied a novel high-content bioassay to test the bioactivity of venoms from a range of *Bothrops* species, alongside venomic approaches for toxin identification. Originally designed to explore the role of habitat type on venom activity, our study found strong evidence for this, with arboreal *Bothrops* lacking cytotoxic activity. Additionally, our study led to the discovery of potential [Ca^2+^]_i_ modulators in *B. mattogrossensis* and *B. pauloensis* inhabiting wetland regions, although future work is required to fully uncover the snake toxin(s) involved in this activity. Being an important area of research, venom-to-drugs research is often favoured over other insightful areas of venom research. In this study, we showed a more holistic view of venom bioactivity and variation, and how these traits can aid venom-to-drugs research, as well as shed light on potential new therapeutic approaches in envenomation treatment.

## Data Availability

The original contributions presented in the study are included in the article/Supplementary Material, further inquiries can be directed to the corresponding author.
