# Supplementary material for: Bothrops venom variation drives niche-specific pharmacology through Ca2+ signalling and membrane damage
Source: Front Pharmacol. 2026 Mar 31;17:1769550. doi: 10.3389/fphar.2026.1769550 (PMC13076241; doi:10.3389/fphar.2026.1769550)
Supplement: Supplementary file 1 [file Supplementaryfile1.docx]

**Supplementary Material 1**

**Supplementary Material 1.1.** Mean and standard deviation of the EC_50_ values (n = 3) for *Bothrops* venoms on HEK293 cells.

| **Venom** | **EC_50_ DNA (mg/ml)** | | **EC_50_ [Ca^2+^]_i_ (mg/ml)** | |
| --- | --- | --- | --- | --- |
|  | **Mean** | **Standard deviation** | **Mean** | **Standard deviation** |
| *B. pauloensis* | 15.42 | 0.97 | 20.62 | 6.15 |
| *B. mattogrossensis* | 14.78 | 4.47 | 19.24 | 6.34 |
| *B. alternatus* | 14.50 | 0.98 | 17.02 | 2.35 |
| *B. caribbaeus* | 12.70 | 1.79 | 20.84 | 4.96 |
| *B. lanceolatus* | 23.74 | 7.19 | 30.79 | 13.22 |
| *B. atrox* | 19.12 | 2.51 | 28.84 | 8.30 |
| *B. asper* | 5.39 | 0.82 | 6.22 | 0.74 |
| *B. leucurus* | 4.02 | 0.30 | 5.32 | 0.25 |
| *B. pictus* | 27.59 | 4.16 | 40.60 | 3.44 |
| *B. diporus* | 2.97 | 0.49 | 3.39 | 0.65 |
| *B. taeniata* | 96.73 | 38.44 | 219.27 | 70.40 |
| *B. oligolepis* | 137.08 | 63.74 | 505.62 | 223.58 |

**Supplementary Material 1.2.** Mean and standard deviation of the EC_50_ values (n = 3) for *Bothrops* venoms on SHSY5Y cells.

| **Venom** | **EC_50_ DNA (mg/ml)** | | **EC_50_ [Ca^2+^]_i_ (mg/ml)** | |
| --- | --- | --- | --- | --- |
|  | **Mean** | **Standard deviation** | **Mean** | **Standard deviation** |
| *B. pauloensis* | 12.85 | 4.13 | 11.90 | 3.34 |
| *B. mattogrossensis* | 13.86 | 5.34 | 12.40 | 4.08 |
| *B. alternatus* | 12.91 | 4.47 | 12.03 | 3.81 |
| *B. caribbaeus* | 13.40 | 3.19 | 13.94 | 3.73 |
| *B. lanceolatus* | 14.69 | 3.57 | 14.52 | 5.98 |
| *B. atrox* | 11.80 | 2.39 | 10.70 | 1.22 |
| *B. asper* | 5.15 | 1.11 | 5.33 | 0.97 |
| *B. leucurus* | 3.82 | 0.54 | 4.31 | 0.29 |
| *B. pictus* | 19.46 | 6.15 | 18.43 | 5.96 |
| *B. diporus* | 3.88 | 0.08 | 3.83 | 0.18 |
| *B. taeniata* | 29.13 | 10.21 | 67.92 | 42.18 |
| *B. oligolepis* | 56.29 | 12.60 | 473.15 | 590.83 |
