# Supplementary material for: Bothrops venom variation drives niche-specific pharmacology through Ca2+ signalling and membrane damage
Source: Front Pharmacol. 2026 Mar 31;17:1769550. doi: 10.3389/fphar.2026.1769550 (PMC13076241; doi:10.3389/fphar.2026.1769550)
Supplement: Supplementary file 2 [file Supplementaryfile3.docx]

**Supplementary Material 3 – statistics**

This supplementary file relates to statistics used in the manuscript.

Specifically, this supplementary file contains:

1. Normality assumption tests (QQ plots) and homodescity of variance tests (residual plot)
2. ANOVA tables
3. Multiple comparisons test tables (Dunnet’s T3)

**HEK293 DNA response (read mode 1) – comparing normalised AUC values at 6.66 mg/ml venom concentration**

**
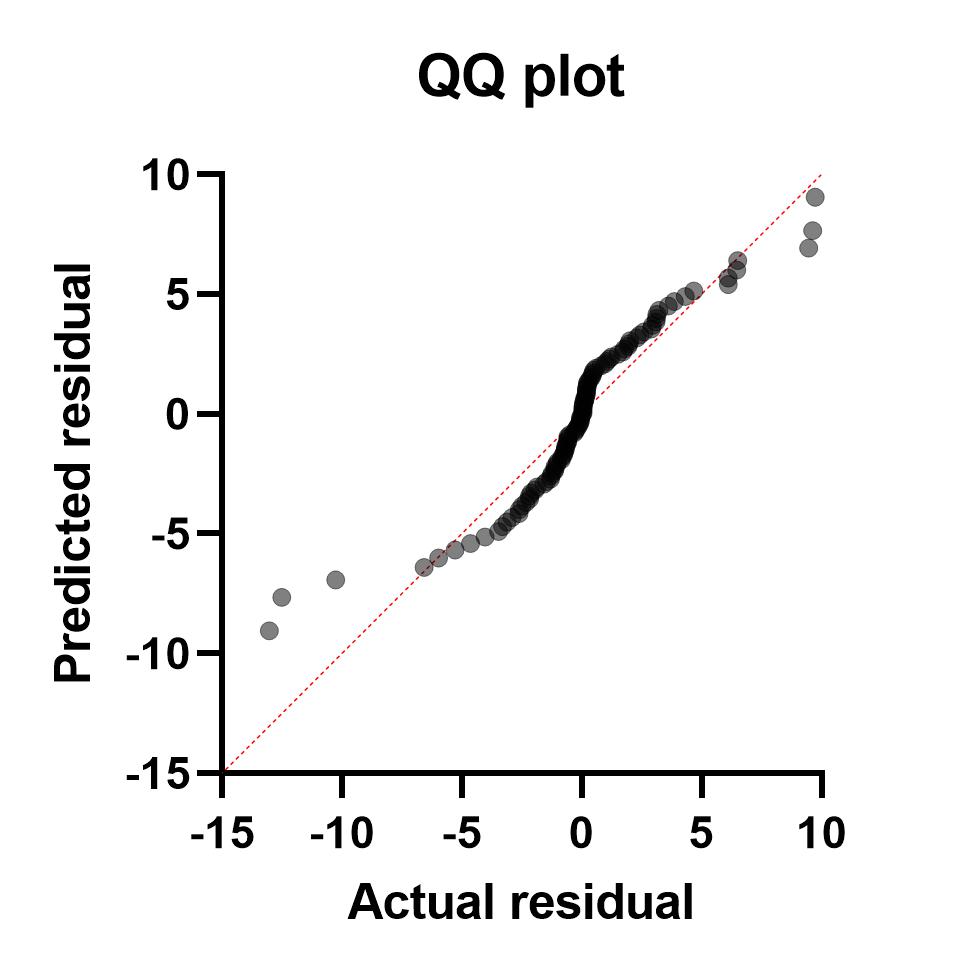
**

**Figure 1. QQ plot.**

**
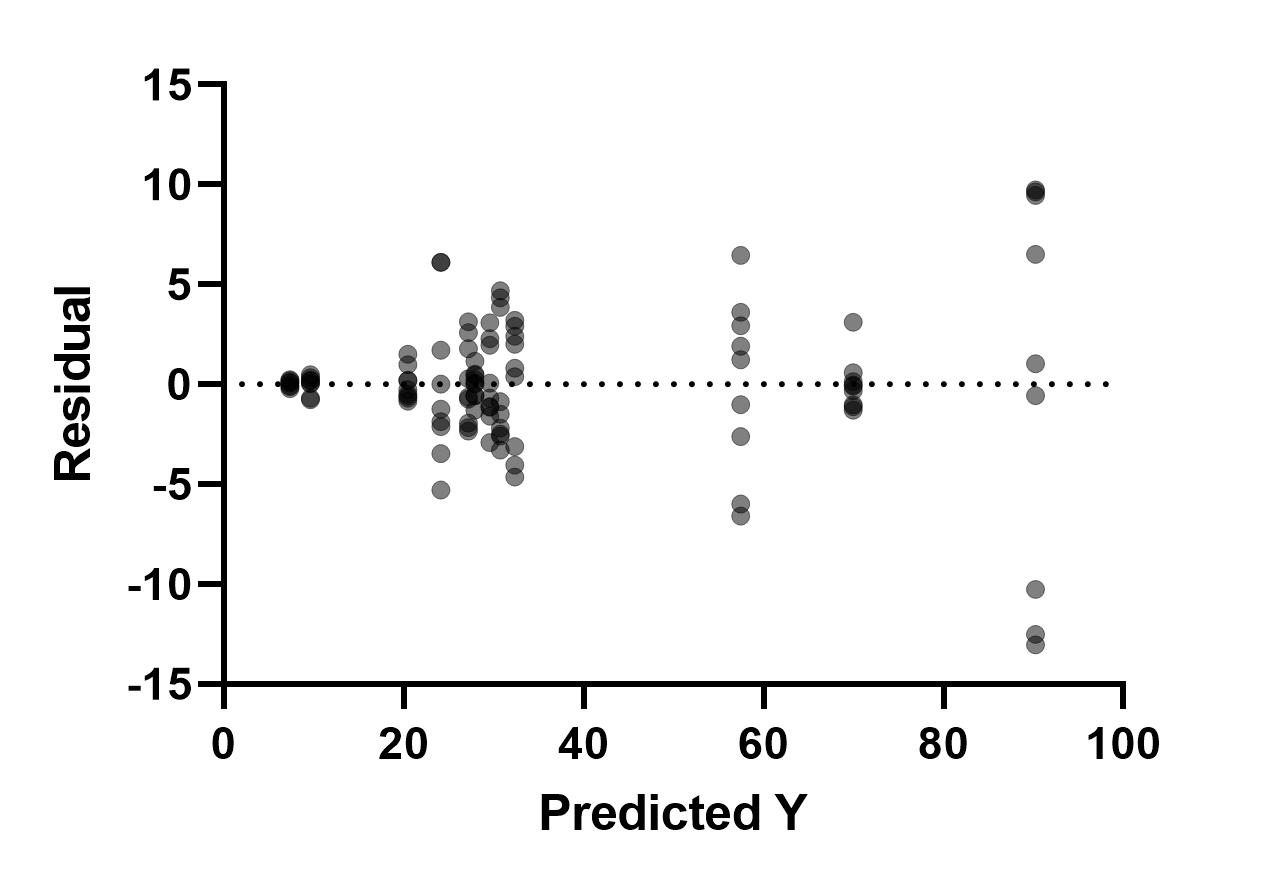
**

**Figure 2. Residual plot**

**Table 1. Brown-Forsythe and Welch ANOVA tests.**

| Brown-Forsythe ANOVA test |  |
| --- | --- |
| F* (DFn, DFd) | 406.1 (11.00, 21.44) |
| P value | <0.0001 |
| P value summary | **** |
| Significant diff. among means (P < 0.05)? | Yes |
|  |  |
| Welch's ANOVA test |  |
| W (DFn, DFd) | 2455 (11.00, 35.80) |
| P value | <0.0001 |
| P value summary | **** |
| Significant diff. among means (P < 0.05)? | Yes |

**Table 2. Dunnets’s T3 multiple comparisons test.**

| Dunnett's T3 multiple comparisons test | Mean Diff. | 95.00% CI of diff. | Summary | Adjusted P Value |
| --- | --- | --- | --- | --- |
| *B. pauloensis* vs. *B. mattogrossensis* | -1.145 | -6.502 to 4.213 | ns | >0.9999 |
| *B. pauloensis* vs. *B. alternatus* | 1.704 | -1.447 to 4.854 | ns | 0.6630 |
| *B. pauloensis* vs. *B. caribbaeus* | -2.766 | -7.818 to 2.285 | ns | 0.7518 |
| *B. pauloensis* vs. *B. lanceolatus* | 5.456 | -0.8371 to 11.75 | ns | 0.1221 |
| *B. pauloensis* vs. *B. atrox* | 2.416 | -1.451 to 6.283 | ns | 0.5889 |
| *B. pauloensis* vs. *B. asper* | -27.87 | -34.83 to -20.90 | **** | <0.0001 |
| *B. pauloensis* vs. *B. leucurus* | -40.35 | -43.65 to -37.05 | **** | <0.0001 |
| *B. pauloensis* vs. *B. pictus* | 9.128 | 5.937 to 12.32 | **** | <0.0001 |
| *B. pauloensis* vs. *B. diporus* | -60.66 | -75.63 to -45.68 | **** | <0.0001 |
| *B. pauloensis* vs. *B. taeniatus* | 19.97 | 16.85 to 23.09 | **** | <0.0001 |
| *B. pauloensis* vs. *B. oligolepis* | 22.27 | 19.11 to 25.44 | **** | <0.0001 |
| *B. mattogrossensis* vs. *B. alternatus* | 2.848 | -2.259 to 7.955 | ns | 0.5829 |
| *B. mattogrossensis* vs. *B. caribbaeus* | -1.622 | -7.661 to 4.418 | ns | >0.9999 |
| *B. mattogrossensis* vs. *B. lanceolatus* | 6.601 | -0.3781 to 13.58 | ns | 0.0747 |
| *B. mattogrossensis* vs. *B. atrox* | 3.560 | -1.844 to 8.965 | ns | 0.4501 |
| *B. mattogrossensis* vs. *B. asper* | -26.72 | -34.15 to -19.29 | **** | <0.0001 |
| *B. mattogrossensis* vs. *B. leucurus* | -39.21 | -44.32 to -34.10 | **** | <0.0001 |
| *B. mattogrossensis* vs. *B. pictus* | 10.27 | 5.139 to 15.41 | *** | 0.0004 |
| *B. mattogrossensis* vs. *B. diporus* | -59.51 | -74.57 to -44.45 | **** | <0.0001 |
| *B. mattogrossensis* vs. *B. taeniatus* | 21.11 | 15.90 to 26.32 | **** | <0.0001 |
| *B. mattogrossensis* vs. *B. oligolepis* | 23.42 | 18.25 to 28.59 | **** | <0.0001 |
| *B. alternatus* vs. *B. caribbaeus* | -4.470 | -9.283 to 0.3438 | ns | 0.0762 |
| *B. alternatus* vs. *B. lanceolatus* | 3.753 | -2.373 to 9.878 | ns | 0.4537 |
| *B. alternatus* vs. *B. atrox* | 0.7121 | -2.528 to 3.952 | ns | >0.9999 |
| *B. alternatus* vs. *B. asper* | -29.57 | -36.57 to -22.57 | **** | <0.0001 |
| *B. alternatus* vs. *B. leucurus* | -42.06 | -44.19 to -39.93 | **** | <0.0001 |
| *B. alternatus* vs. *B. pictus* | 7.425 | 5.984 to 8.865 | **** | <0.0001 |
| *B. alternatus* vs. *B. diporus* | -62.36 | -77.58 to -47.14 | **** | <0.0001 |
| *B. alternatus* vs. *B. taeniatus* | 18.26 | 17.10 to 19.43 | **** | <0.0001 |
| *B. alternatus* vs. *B. oligolepis* | 20.57 | 19.45 to 21.69 | **** | <0.0001 |
| *B. caribbaeus* vs. *B. lanceolatus* | 8.222 | 1.411 to 15.03 | * | 0.0105 |
| *B. caribbaeus* vs. *B. atrox* | 5.182 | 0.08179 to 10.28 | * | 0.0445 |
| *B. caribbaeus* vs. *B. asper* | -25.10 | -32.46 to -17.74 | **** | <0.0001 |
| *B. caribbaeus* vs. *B. leucurus* | -37.59 | -42.43 to -32.74 | **** | <0.0001 |
| *B. caribbaeus* vs. *B. pictus* | 11.89 | 7.053 to 16.74 | **** | <0.0001 |
| *B. caribbaeus* vs. *B. diporus* | -57.89 | -72.86 to -42.92 | **** | <0.0001 |
| *B. caribbaeus* vs. *B. taeniatus* | 22.73 | 17.83 to 27.63 | **** | <0.0001 |
| *B. caribbaeus* vs. *B. oligolepis* | 25.04 | 20.18 to 29.90 | **** | <0.0001 |
| *B. lanceolatus* vs. *B. atrox* | -3.041 | -9.375 to 3.294 | ns | 0.8601 |
| *B. lanceolatus* vs. *B. asper* | -33.32 | -41.25 to -25.39 | **** | <0.0001 |
| *B. lanceolatus* vs. *B. leucurus* | -45.81 | -51.99 to -39.63 | **** | <0.0001 |
| *B. lanceolatus* vs. *B. pictus* | 3.672 | -2.476 to 9.820 | ns | 0.4883 |
| *B. lanceolatus* vs. *B. diporus* | -66.11 | -81.17 to -51.05 | **** | <0.0001 |
| *B. lanceolatus* vs. *B. taeniatus* | 14.51 | 8.235 to 20.79 | *** | 0.0002 |
| *B. lanceolatus* vs. *B. oligolepis* | 16.82 | 10.57 to 23.06 | **** | <0.0001 |
| *B. atrox* vs. *B. asper* | -30.28 | -37.29 to -23.28 | **** | <0.0001 |
| *B. atrox* vs. *B. leucurus* | -42.77 | -46.14 to -39.40 | **** | <0.0001 |
| *B. atrox* vs. *B. pictus* | 6.712 | 3.433 to 9.992 | *** | 0.0002 |
| *B. atrox* vs. *B. diporus* | -63.07 | -78.07 to -48.08 | **** | <0.0001 |
| *B. atrox* vs. *B. taeniatus* | 17.55 | 14.34 to 20.77 | **** | <0.0001 |
| *B. atrox* vs. *B. oligolepis* | 19.86 | 16.59 to 23.13 | **** | <0.0001 |
| *B. asper* vs. *B. leucurus* | -12.49 | -19.45 to -5.524 | *** | 0.0008 |
| *B. asper* vs. *B. pictus* | 36.99 | 30.22 to 43.77 | **** | <0.0001 |
| *B. asper* vs. *B. diporus* | -32.79 | -48.09 to -17.49 | **** | <0.0001 |
| *B. asper* vs. *B. taeniatus* | 47.83 | 40.90 to 54.77 | **** | <0.0001 |
| *B. asper* vs. *B. oligolepis* | 50.14 | 43.23 to 57.05 | **** | <0.0001 |
| *B. leucurus* vs. *B. pictus* | 49.48 | 47.33 to 51.63 | **** | <0.0001 |
| *B. leucurus* vs. *B. diporus* | -20.30 | -35.63 to -4.981 | ** | 0.0092 |
| *B. leucurus* vs. *B. taeniatus* | 60.32 | 58.27 to 62.37 | **** | <0.0001 |
| *B. leucurus* vs. *B. oligolepis* | 62.63 | 60.53 to 64.72 | **** | <0.0001 |
| *B. pictus* vs. *B. diporus* | -69.78 | -85.02 to -54.55 | **** | <0.0001 |
| *B. pictus* vs. *B. taeniatus* | 10.84 | 9.559 to 12.12 | **** | <0.0001 |
| *B. pictus* vs. *B. oligolepis* | 13.15 | 11.91 to 14.38 | **** | <0.0001 |
| *B. diporus* vs. *B. taeniatus* | 80.62 | 65.43 to 95.82 | **** | <0.0001 |
| *B. diporus* vs. *B. oligolepis* | 82.93 | 67.75 to 98.11 | **** | <0.0001 |
| *B. taeniatus* vs. *B. oligolepis* | 2.305 | 1.632 to 2.979 | **** | <0.0001 |

**HEK293 calcium response (read mode 2) – comparing normalised AUC values at 6.66 mg/ml venom concentration**


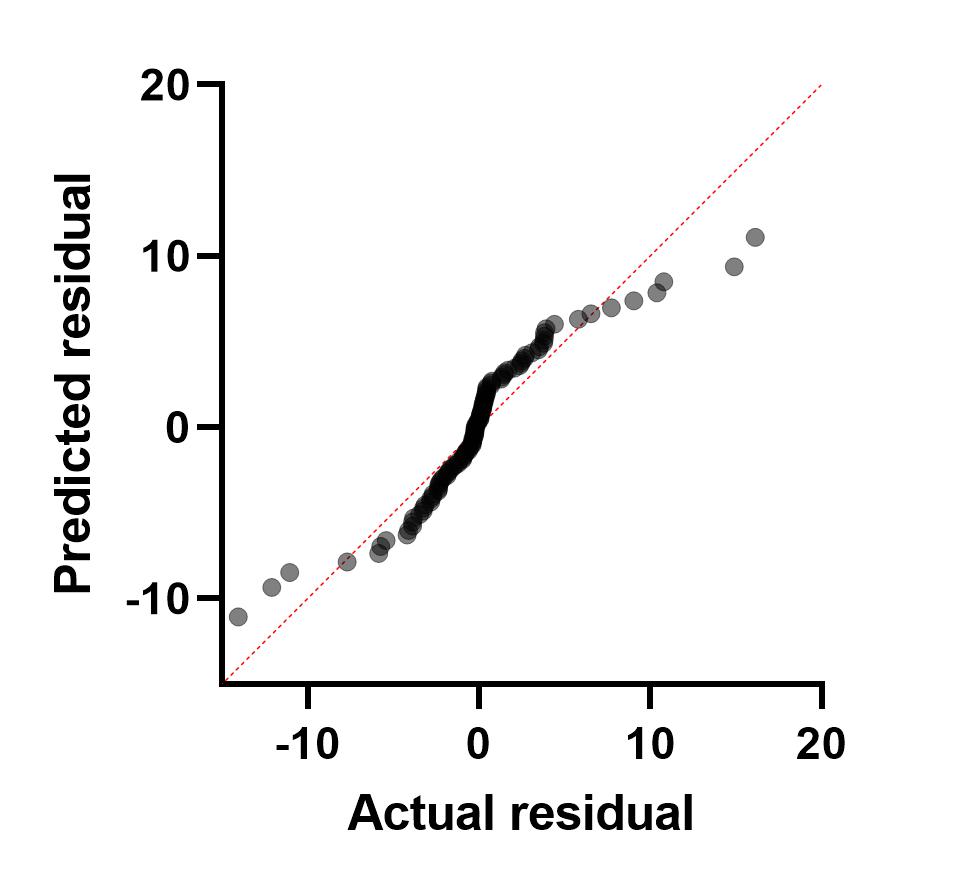


**Figure 3. QQ plot.**

**
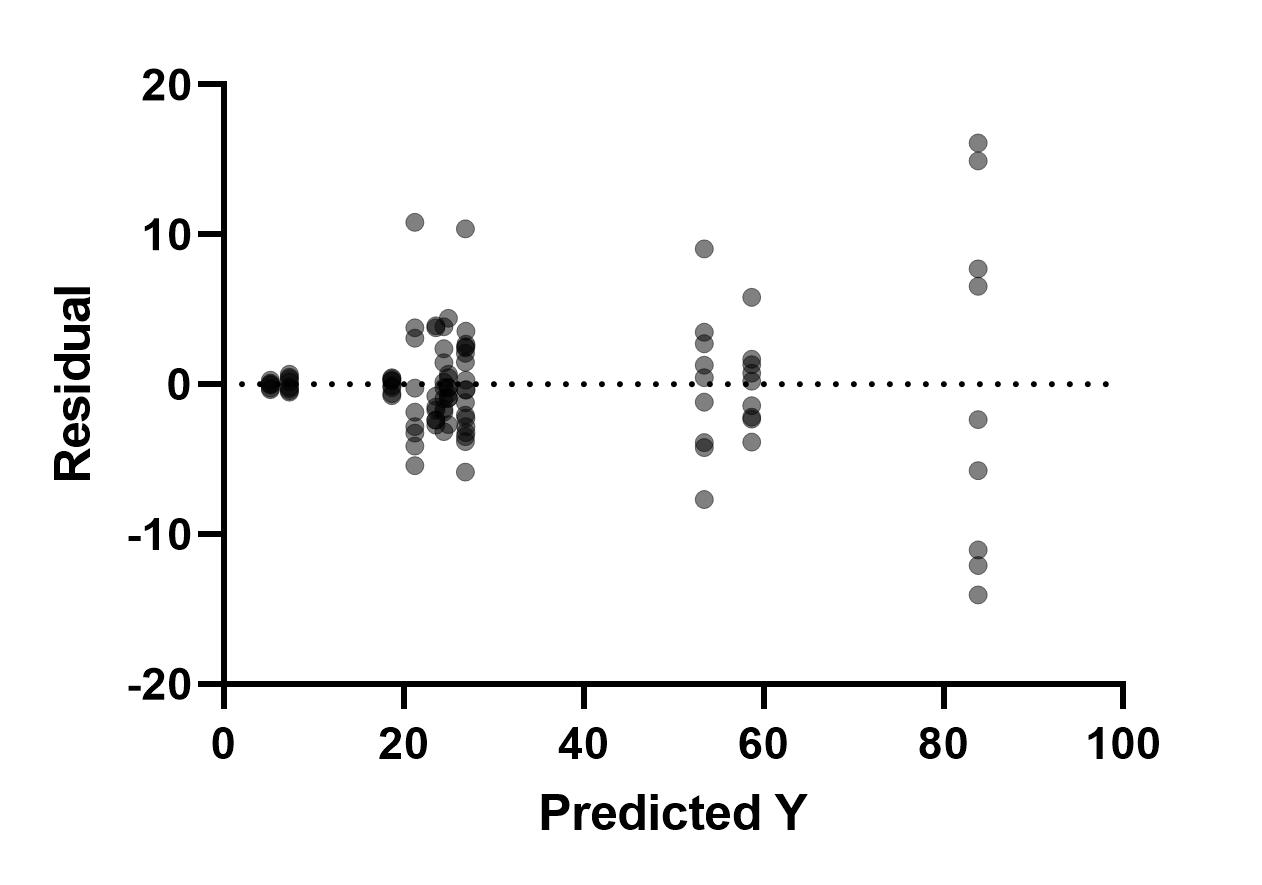
**

**Figure 4. Residual plot**

**Table 3. Brown-Forsythe and Welch ANOVA tests.**

| Brown-Forsythe ANOVA test |  |
| --- | --- |
| F* (DFn, DFd) | 231.1 (11.00, 22.83) |
| P value | <0.0001 |
| P value summary | **** |
| Significant diff. among means (P < 0.05)? | Yes |
|  |  |
| Welch's ANOVA test |  |
| W (DFn, DFd) | 1020 (11.00, 36.19) |
| P value | <0.0001 |
| P value summary | **** |
| Significant diff. among means (P < 0.05)? | Yes |

**Table 4. Dunnets’s T3 multiple comparisons test.**

| Dunnett's T3 multiple comparisons test | Mean Diff. | 95.00% CI of diff. | Summary | Adjusted P Value |
| --- | --- | --- | --- | --- |
| *B. pauloensis* vs. *B. mattogrossensis* | -0.08829 | -7.751 to 7.574 | ns | >0.9999 |
| *B. pauloensis* vs. *B. alternatus* | 1.870 | -5.778 to 9.519 | ns | >0.9999 |
| *B. pauloensis* vs. *B. caribbaeus* | 2.364 | -5.280 to 10.01 | ns | 0.9981 |
| *B. pauloensis* vs. *B. lanceolatus* | 5.611 | -3.772 to 14.99 | ns | 0.6596 |
| *B. pauloensis* vs. *B. atrox* | 3.270 | -4.566 to 11.11 | ns | 0.9627 |
| *B. pauloensis* vs. *B. asper* | -26.57 | -35.81 to -17.34 | **** | <0.0001 |
| *B. pauloensis* vs. *B. leucurus* | -31.88 | -39.67 to -24.09 | **** | <0.0001 |
| *B. pauloensis* vs. *B. pictus* | 8.113 | 0.5137 to 15.71 | * | 0.0346 |
| *B. pauloensis* vs. *B. diporus* | -57.04 | -75.17 to -38.90 | **** | <0.0001 |
| *B. pauloensis* vs. *B. taeniatus* | 19.54 | 11.94 to 27.14 | **** | <0.0001 |
| *B. pauloensis* vs. *B. oligolepis* | 21.61 | 14.04 to 29.19 | **** | <0.0001 |
| *B. mattogrossensis* vs. *B. alternatus* | 1.959 | -2.289 to 6.206 | ns | 0.9339 |
| *B. mattogrossensis* vs. *B. caribbaeus* | 2.452 | -2.007 to 6.912 | ns | 0.7815 |
| *B. mattogrossensis* vs. *B. lanceolatus* | 5.699 | -2.309 to 13.71 | ns | 0.3218 |
| *B. mattogrossensis* vs. *B. atrox* | 3.359 | -1.781 to 8.498 | ns | 0.5138 |
| *B. mattogrossensis* vs. *B. asper* | -26.48 | -34.30 to -18.67 | **** | <0.0001 |
| *B. mattogrossensis* vs. *B. leucurus* | -31.79 | -36.86 to -26.72 | **** | <0.0001 |
| *B. mattogrossensis* vs. *B. pictus* | 8.201 | 4.371 to 12.03 | *** | 0.0002 |
| *B. mattogrossensis* vs. *B. diporus* | -56.95 | -74.98 to -38.91 | **** | <0.0001 |
| *B. mattogrossensis* vs. *B. taeniatus* | 19.63 | 15.80 to 23.46 | **** | <0.0001 |
| *B. mattogrossensis* vs. *B. oligolepis* | 21.70 | 17.78 to 25.62 | **** | <0.0001 |
| *B. alternatus* vs. *B. caribbaeus* | 0.4937 | -3.430 to 4.418 | ns | >0.9999 |
| *B. alternatus* vs. *B. lanceolatus* | 3.741 | -4.285 to 11.77 | ns | 0.8388 |
| *B. alternatus* vs. *B. atrox* | 1.400 | -3.395 to 6.194 | ns | 0.9998 |
| *B. alternatus* vs. *B. asper* | -28.44 | -36.26 to -20.63 | **** | <0.0001 |
| *B. alternatus* vs. *B. leucurus* | -33.75 | -38.47 to -29.03 | **** | <0.0001 |
| *B. alternatus* vs. *B. pictus* | 6.243 | 3.259 to 9.226 | *** | 0.0002 |
| *B. alternatus* vs. *B. diporus* | -58.91 | -77.41 to -40.40 | **** | <0.0001 |
| *B. alternatus* vs. *B. taeniatus* | 17.67 | 14.69 to 20.66 | **** | <0.0001 |
| *B. alternatus* vs. *B. oligolepis* | 19.74 | 16.72 to 22.77 | **** | <0.0001 |
| *B. caribbaeus* vs. *B. lanceolatus* | 3.247 | -4.758 to 11.25 | ns | 0.9538 |
| *B. caribbaeus* vs. *B. atrox* | 0.9062 | -4.064 to 5.876 | ns | >0.9999 |
| *B. caribbaeus* vs. *B. asper* | -28.94 | -36.74 to -21.13 | **** | <0.0001 |
| *B. caribbaeus* vs. *B. leucurus* | -34.24 | -39.14 to -29.34 | **** | <0.0001 |
| *B. caribbaeus* vs. *B. pictus* | 5.749 | 2.318 to 9.180 | ** | 0.0014 |
| *B. caribbaeus* vs. *B. diporus* | -59.40 | -77.36 to -41.44 | **** | <0.0001 |
| *B. caribbaeus* vs. *B. taeniatus* | 17.18 | 13.75 to 20.61 | **** | <0.0001 |
| *B. caribbaeus* vs. *B. oligolepis* | 19.25 | 15.75 to 22.75 | **** | <0.0001 |
| *B. lanceolatus* vs. *B. atrox* | -2.341 | -10.51 to 5.824 | ns | 0.9998 |
| *B. lanceolatus* vs. *B. asper* | -32.18 | -41.68 to -22.69 | **** | <0.0001 |
| *B. lanceolatus* vs. *B. leucurus* | -37.49 | -45.61 to -29.37 | **** | <0.0001 |
| *B. lanceolatus* vs. *B. pictus* | 2.502 | -5.526 to 10.53 | ns | 0.9884 |
| *B. lanceolatus* vs. *B. diporus* | -62.65 | -80.94 to -44.35 | **** | <0.0001 |
| *B. lanceolatus* vs. *B. taeniatus* | 13.93 | 5.905 to 21.96 | ** | 0.0015 |
| *B. lanceolatus* vs. *B. oligolepis* | 16.00 | 7.998 to 24.00 | *** | 0.0005 |
| *B. atrox* vs. *B. asper* | -29.84 | -37.83 to -21.86 | **** | <0.0001 |
| *B. atrox* vs. *B. leucurus* | -35.15 | -40.62 to -29.67 | **** | <0.0001 |
| *B. atrox* vs. *B. pictus* | 4.843 | 0.1931 to 9.492 | * | 0.0398 |
| *B. atrox* vs. *B. diporus* | -60.31 | -78.49 to -42.12 | **** | <0.0001 |
| *B. atrox* vs. *B. taeniatus* | 16.27 | 11.62 to 20.92 | **** | <0.0001 |
| *B. atrox* vs. *B. oligolepis* | 18.34 | 13.73 to 22.95 | **** | <0.0001 |
| *B. asper* vs. *B. leucurus* | -5.304 | -13.24 to 2.631 | ns | 0.4278 |
| *B. asper* vs. *B. pictus* | 34.69 | 26.90 to 42.48 | **** | <0.0001 |
| *B. asper* vs. *B. diporus* | -30.46 | -48.67 to -12.26 | *** | 0.0009 |
| *B. asper* vs. *B. taeniatus* | 46.12 | 38.33 to 53.91 | **** | <0.0001 |
| *B. asper* vs. *B. oligolepis* | 48.19 | 40.42 to 55.95 | **** | <0.0001 |
| *B. leucurus* vs. *B. pictus* | 39.99 | 35.44 to 44.54 | **** | <0.0001 |
| *B. leucurus* vs. *B. diporus* | -25.16 | -43.32 to -6.995 | ** | 0.0059 |
| *B. leucurus* vs. *B. taeniatus* | 51.42 | 46.87 to 55.97 | **** | <0.0001 |
| *B. leucurus* vs. *B. oligolepis* | 53.49 | 48.99 to 57.99 | **** | <0.0001 |
| *B. pictus* vs. *B. diporus* | -65.15 | -83.42 to -46.87 | **** | <0.0001 |
| *B. pictus* vs. *B. taeniatus* | 11.43 | 10.59 to 12.27 | **** | <0.0001 |
| *B. pictus* vs. *B. oligolepis* | 13.50 | 12.81 to 14.19 | **** | <0.0001 |
| *B. diporus* vs. *B. taeniatus* | 76.58 | 58.30 to 94.86 | **** | <0.0001 |
| *B. diporus* vs. *B. oligolepis* | 78.65 | 60.38 to 96.91 | **** | <0.0001 |
| *B. taeniatus* vs. *B. oligolepis* | 2.068 | 1.370 to 2.766 | **** | <0.0001 |

**SHSY5Y LogEC50 DNA response (read mode 1) – comparing normalised AUC values at 6.66 mg/ml venom concentration**


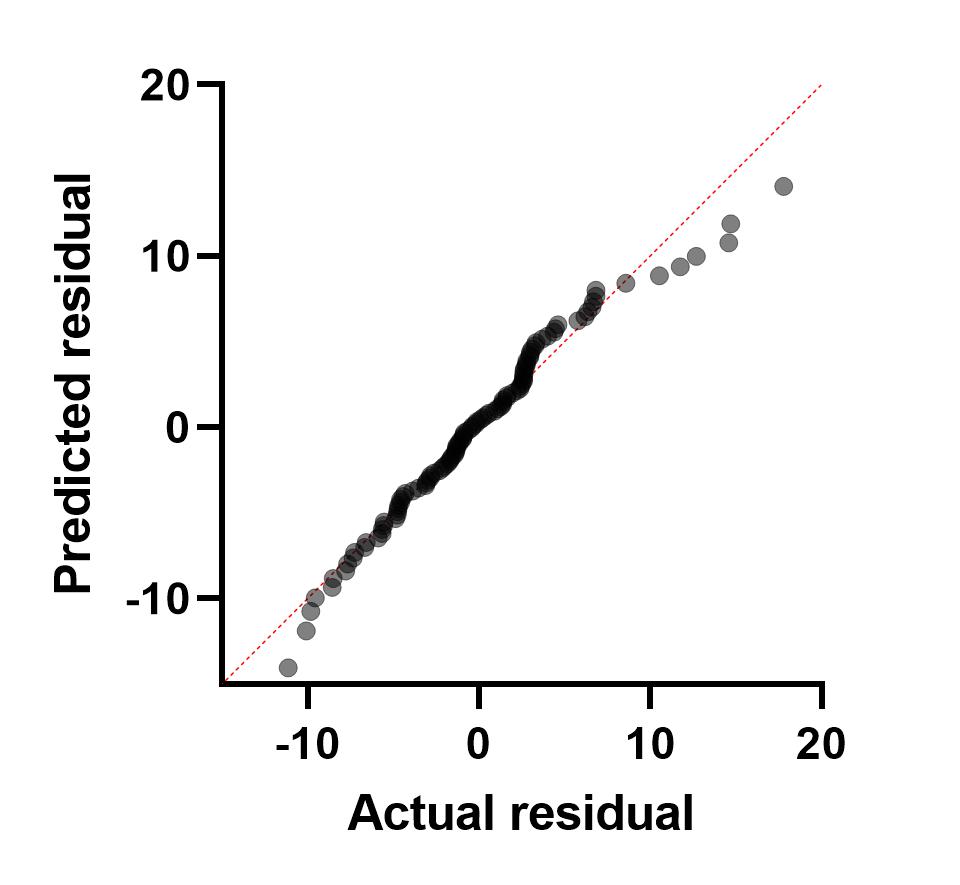


**Figure 5. QQ plot.**

**
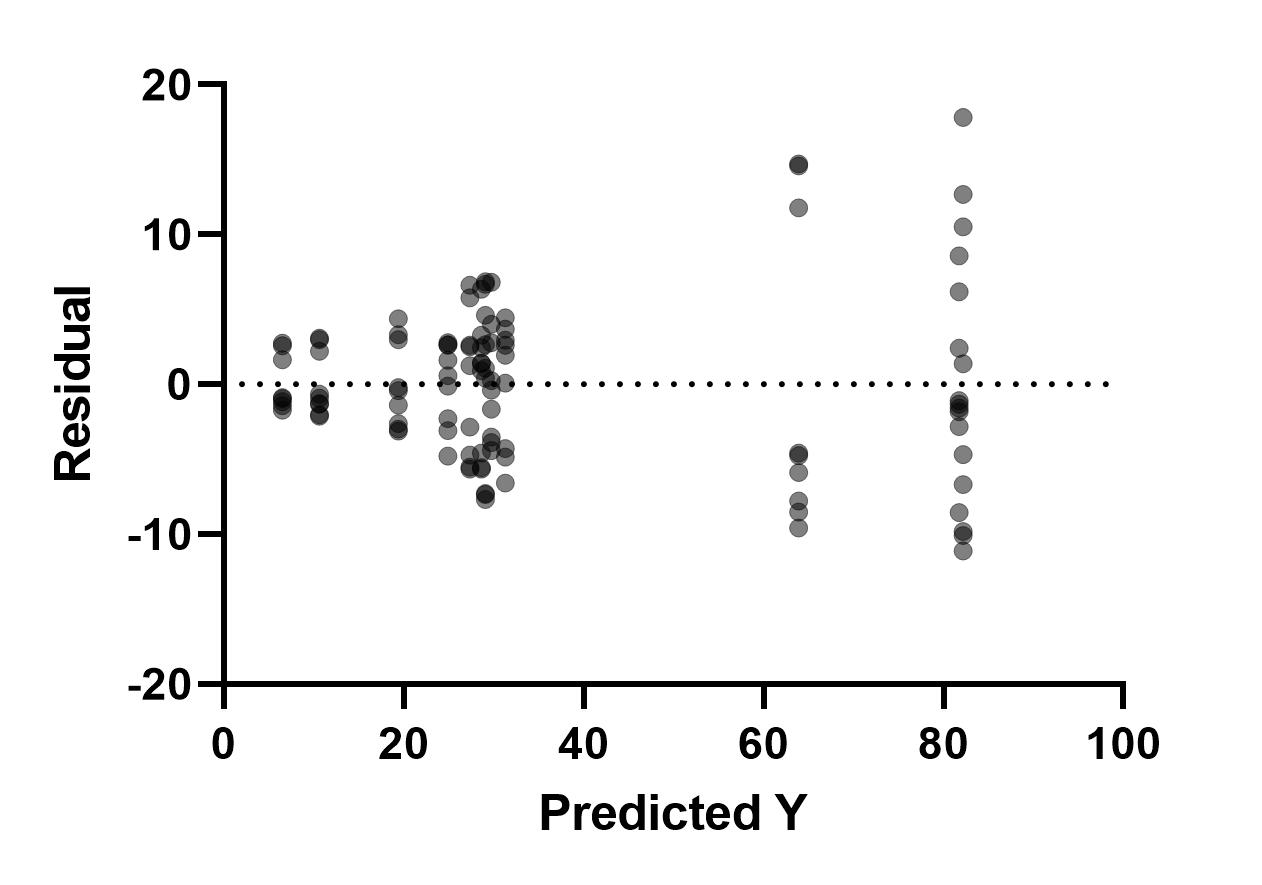
**

**Figure 6. Residual plot**

**Table 5. Brown-Forsythe and Welch ANOVA tests.**

| Brown-Forsythe ANOVA test |  |
| --- | --- |
| F* (DFn, DFd) | 179.8 (11.00, 40.24) |
| P value | <0.0001 |
| P value summary | **** |
| Significant diff. among means (P < 0.05)? | Yes |
|  |  |
| Welch's ANOVA test |  |
| W (DFn, DFd) | 207.0 (11.00, 37.52) |
| P value | <0.0001 |
| P value summary | **** |
| Significant diff. among means (P < 0.05)? | Yes |

**Table 6. Dunnets’s T3 multiple comparisons test.**

| Dunnett's T3 multiple comparisons test | Mean Diff. | 95.00% CI of diff. | Below threshold? | Summary | Adjusted P Value |
| --- | --- | --- | --- | --- | --- |
| *B. pauloensis* vs. *B. mattogrossensis* | 1.116 | -6.576 to 8.809 | No | ns | >0.9999 |
| *B. pauloensis* vs. *B. alternatus* | 0.6617 | -9.081 to 10.40 | No | ns | >0.9999 |
| *B. pauloensis* vs. *B. caribbaeus* | 2.398 | -5.925 to 10.72 | No | ns | >0.9999 |
| *B. pauloensis* vs. *B. lanceolatus* | 4.829 | -1.614 to 11.27 | No | ns | 0.2890 |
| *B. pauloensis* vs. *B. atrox* | -1.559 | -9.141 to 6.023 | No | ns | >0.9999 |
| *B. pauloensis* vs. *B. asper* | -34.14 | -50.50 to -17.79 | Yes | *** | 0.0002 |
| *B. pauloensis* vs. *B. leucurus* | -52.45 | -69.67 to -35.22 | Yes | **** | <0.0001 |
| *B. pauloensis* vs. *B. pictus* | 10.34 | 3.819 to 16.86 | Yes | *** | 0.0007 |
| *B. pauloensis* vs. *B. diporus* | -51.99 | -60.63 to -43.35 | Yes | **** | <0.0001 |
| *B. pauloensis* vs. *B. taeniatus* | 19.10 | 12.87 to 25.33 | Yes | **** | <0.0001 |
| *B. pauloensis* vs. *B. oligolepis* | 23.25 | 17.12 to 29.38 | Yes | **** | <0.0001 |
| *B. mattogrossensis* vs. *B. alternatus* | -0.4547 | -10.50 to 9.590 | No | ns | >0.9999 |
| *B. mattogrossensis* vs. *B. caribbaeus* | 1.282 | -7.289 to 9.853 | No | ns | >0.9999 |
| *B. mattogrossensis* vs. *B. lanceolatus* | 3.712 | -3.254 to 10.68 | No | ns | 0.7870 |
| *B. mattogrossensis* vs. *B. atrox* | -2.675 | -10.62 to 5.274 | No | ns | 0.9991 |
| *B. mattogrossensis* vs. *B. asper* | -35.26 | -51.44 to -19.07 | Yes | **** | <0.0001 |
| *B. mattogrossensis* vs. *B. leucurus* | -53.56 | -70.99 to -36.13 | Yes | **** | <0.0001 |
| *B. mattogrossensis* vs. *B. pictus* | 9.224 | 2.183 to 16.27 | Yes | ** | 0.0053 |
| *B. mattogrossensis* vs. *B. diporus* | -53.11 | -61.98 to -44.24 | Yes | **** | <0.0001 |
| *B. mattogrossensis* vs. *B. taeniatus* | 17.98 | 11.26 to 24.71 | Yes | **** | <0.0001 |
| *B. mattogrossensis* vs. *B. oligolepis* | 22.14 | 15.49 to 28.78 | Yes | **** | <0.0001 |
| *B. alternatus* vs. *B. caribbaeus* | 1.737 | -8.616 to 12.09 | No | ns | >0.9999 |
| *B. alternatus* vs. *B. lanceolatus* | 4.167 | -5.324 to 13.66 | No | ns | 0.9113 |
| *B. alternatus* vs. *B. atrox* | -2.221 | -12.18 to 7.736 | No | ns | >0.9999 |
| *B. alternatus* vs. *B. asper* | -34.80 | -51.46 to -18.14 | Yes | **** | <0.0001 |
| *B. alternatus* vs. *B. leucurus* | -53.11 | -70.82 to -35.39 | Yes | **** | <0.0001 |
| *B. alternatus* vs. *B. pictus* | 9.679 | 0.3114 to 19.05 | Yes | * | 0.0399 |
| *B. alternatus* vs. *B. diporus* | -52.65 | -63.14 to -42.16 | Yes | **** | <0.0001 |
| *B. alternatus* vs. *B. taeniatus* | 18.44 | 9.093 to 27.78 | Yes | *** | 0.0003 |
| *B. alternatus* vs. *B. oligolepis* | 22.59 | 13.14 to 32.04 | Yes | **** | <0.0001 |
| *B. caribbaeus* vs. *B. lanceolatus* | 2.430 | -5.265 to 10.13 | No | ns | 0.9990 |
| *B. caribbaeus* vs. *B. atrox* | -3.957 | -12.43 to 4.515 | No | ns | 0.9353 |
| *B. caribbaeus* vs. *B. asper* | -36.54 | -53.03 to -20.05 | Yes | **** | <0.0001 |
| *B. caribbaeus* vs. *B. leucurus* | -54.84 | -72.16 to -37.52 | Yes | **** | <0.0001 |
| *B. caribbaeus* vs. *B. pictus* | 7.942 | 0.1775 to 15.71 | Yes | * | 0.0426 |
| *B. caribbaeus* vs. *B. diporus* | -54.39 | -63.73 to -45.05 | Yes | **** | <0.0001 |
| *B. caribbaeus* vs. *B. taeniatus* | 16.70 | 9.155 to 24.25 | Yes | **** | <0.0001 |
| *B. caribbaeus* vs. *B. oligolepis* | 20.86 | 13.32 to 28.39 | Yes | **** | <0.0001 |
| *B. lanceolatus* vs. *B. atrox* | -6.388 | -13.23 to 0.4506 | No | ns | 0.0804 |
| *B. lanceolatus* vs. *B. asper* | -38.97 | -55.29 to -22.65 | Yes | **** | <0.0001 |
| *B. lanceolatus* vs. *B. leucurus* | -57.27 | -74.52 to -40.03 | Yes | **** | <0.0001 |
| *B. lanceolatus* vs. *B. pictus* | 5.512 | 0.1515 to 10.87 | Yes | * | 0.0402 |
| *B. lanceolatus* vs. *B. diporus* | -56.82 | -65.00 to -48.63 | Yes | **** | <0.0001 |
| *B. lanceolatus* vs. *B. taeniatus* | 14.27 | 9.533 to 19.01 | Yes | **** | <0.0001 |
| *B. lanceolatus* vs. *B. oligolepis* | 18.42 | 13.90 to 22.95 | Yes | **** | <0.0001 |
| *B. atrox* vs. *B. asper* | -32.58 | -49.09 to -16.08 | Yes | *** | 0.0003 |
| *B. atrox* vs. *B. leucurus* | -50.89 | -68.26 to -33.52 | Yes | **** | <0.0001 |
| *B. atrox* vs. *B. pictus* | 11.90 | 4.985 to 18.81 | Yes | *** | 0.0003 |
| *B. atrox* vs. *B. diporus* | -50.43 | -59.30 to -41.56 | Yes | **** | <0.0001 |
| *B. atrox* vs. *B. taeniatus* | 20.66 | 14.08 to 27.24 | Yes | **** | <0.0001 |
| *B. atrox* vs. *B. oligolepis* | 24.81 | 18.31 to 31.31 | Yes | **** | <0.0001 |
| *B. asper* vs. *B. leucurus* | -18.30 | -38.58 to 1.974 | No | ns | 0.1040 |
| *B. asper* vs. *B. pictus* | 44.48 | 28.12 to 60.84 | Yes | **** | <0.0001 |
| *B. asper* vs. *B. diporus* | -17.85 | -34.20 to -1.499 | Yes | * | 0.0269 |
| *B. asper* vs. *B. taeniatus* | 53.24 | 37.15 to 69.34 | Yes | **** | <0.0001 |
| *B. asper* vs. *B. oligolepis* | 57.40 | 40.84 to 73.96 | Yes | **** | <0.0001 |
| *B. leucurus* vs. *B. pictus* | 62.79 | 45.50 to 80.07 | Yes | **** | <0.0001 |
| *B. leucurus* vs. *B. diporus* | 0.4549 | -17.04 to 17.95 | No | ns | >0.9999 |
| *B. leucurus* vs. *B. taeniatus* | 71.55 | 54.52 to 88.58 | Yes | **** | <0.0001 |
| *B. leucurus* vs. *B. oligolepis* | 75.70 | 58.16 to 93.24 | Yes | **** | <0.0001 |
| *B. pictus* vs. *B. diporus* | -62.33 | -70.45 to -54.21 | Yes | **** | <0.0001 |
| *B. pictus* vs. *B. taeniatus* | 8.760 | 3.915 to 13.60 | Yes | *** | 0.0002 |
| *B. pictus* vs. *B. oligolepis* | 12.91 | 8.206 to 17.62 | Yes | **** | <0.0001 |
| *B. diporus* vs. *B. taeniatus* | 71.09 | 63.16 to 79.03 | Yes | **** | <0.0001 |
| *B. diporus* vs. *B. oligolepis* | 75.24 | 67.31 to 83.18 | Yes | **** | <0.0001 |
| *B. taeniatus* vs. *B. oligolepis* | 4.153 | 0.4426 to 7.864 | Yes | * | 0.0199 |

**SHSY5Y LogEC50 calcium response (read mode 2) – comparing normalised AUC values at 6.66 mg/ml venom concentration**

**
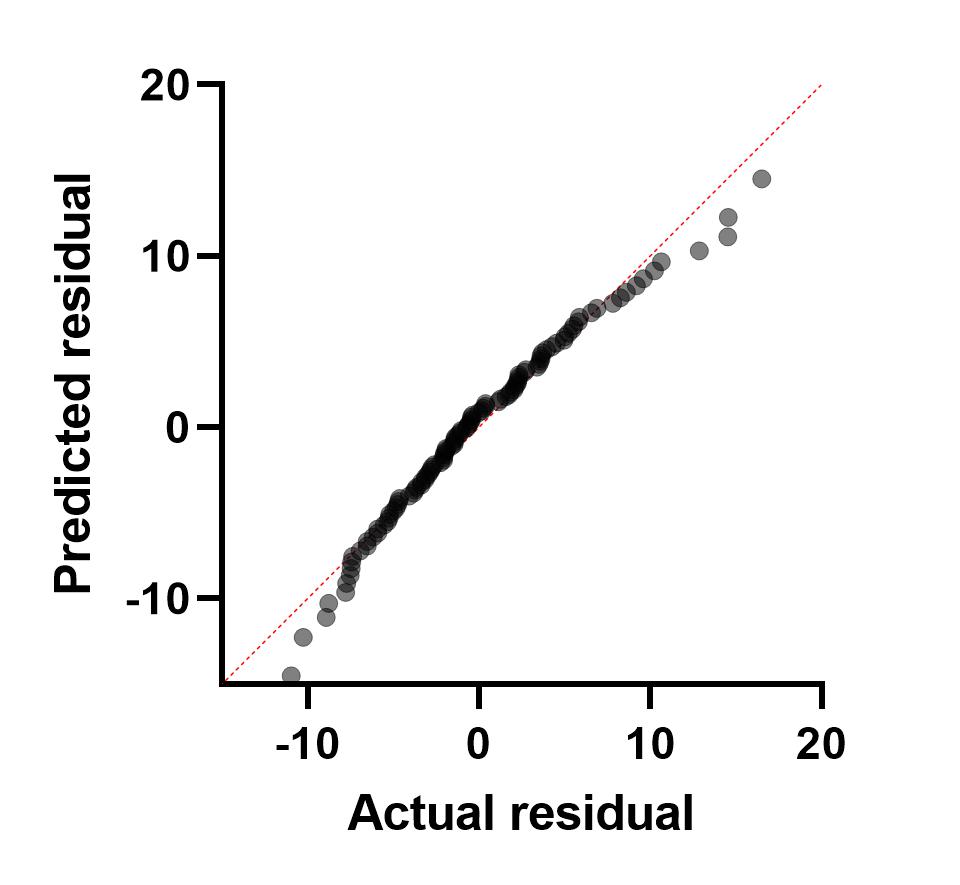
**

**Figure 7. QQ plot.**

**
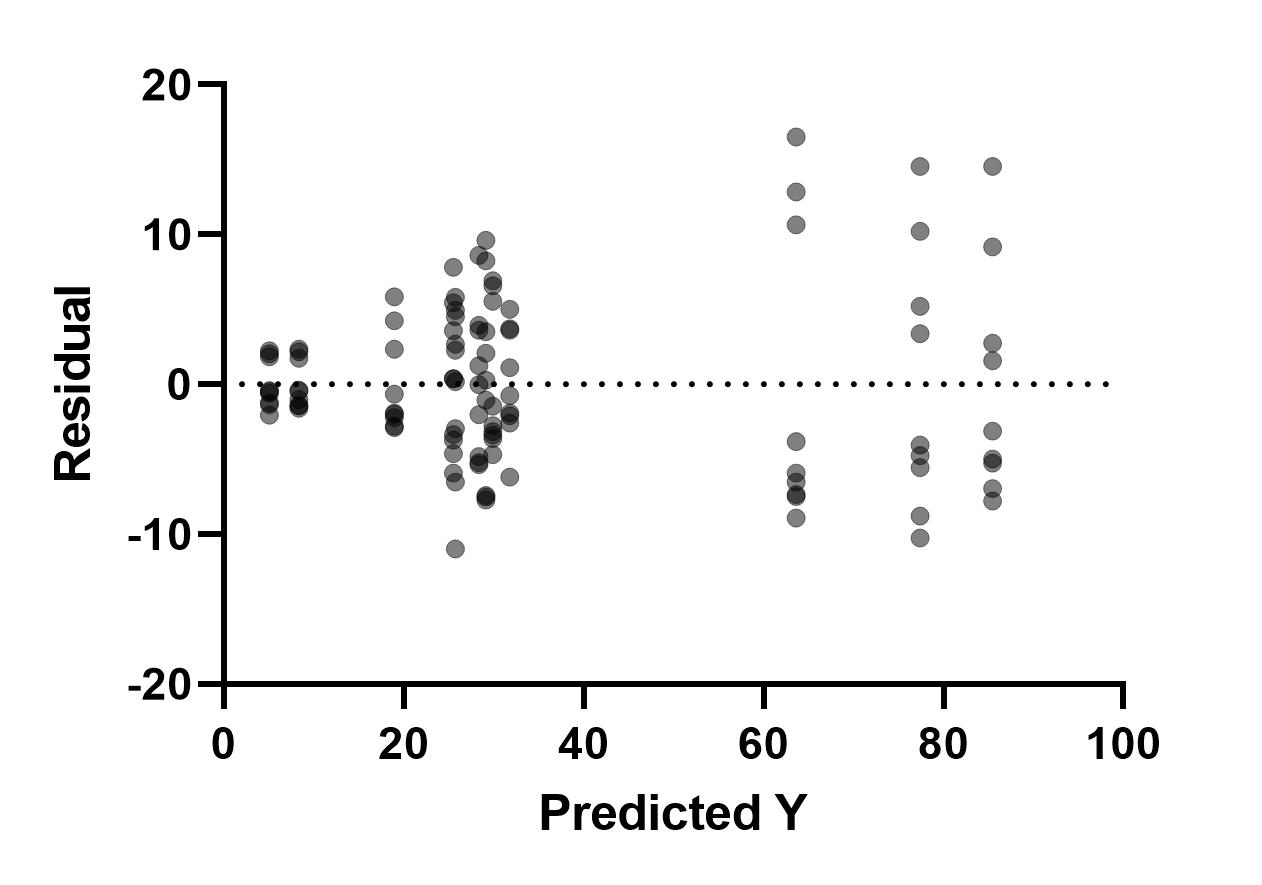
**

**Figure 8. Residual plot**

**Table 7. Brown-Forsythe and Welch ANOVA tests.**

| Brown-Forsythe ANOVA test |  |
| --- | --- |
| F* (DFn, DFd) | 172.6 (11.00, 55.13) |
| P value | <0.0001 |
| P value summary | **** |
| Significant diff. among means (P < 0.05)? | Yes |
|  |  |
| Welch's ANOVA test |  |
| W (DFn, DFd) | 183.2 (11.00, 37.26) |
| P value | <0.0001 |
| P value summary | **** |
| Significant diff. among means (P < 0.05)? | Yes |

**Table 8. Dunnets’s T3 multiple comparisons test.**

| Dunnett's T3 multiple comparisons test | Mean Diff. | 95.00% CI of diff. | Below threshold? | Summary | Adjusted P Value |
| --- | --- | --- | --- | --- | --- |
| *B. pauloensis* vs. *B. mattogrossensis* | 1.536 | -7.597 to 10.67 | No | ns | >0.9999 |
| *B. pauloensis* vs. *B. alternatus* | 0.7903 | -10.26 to 11.84 | No | ns | >0.9999 |
| *B. pauloensis* vs. *B. caribbaeus* | 4.403 | -4.704 to 13.51 | No | ns | 0.9127 |
| *B. pauloensis* vs. *B. lanceolatus* | 4.140 | -5.867 to 14.15 | No | ns | 0.9816 |
| *B. pauloensis* vs. *B. atrox* | -1.904 | -10.08 to 6.276 | No | ns | >0.9999 |
| *B. pauloensis* vs. *B. asper* | -33.73 | -49.95 to -17.51 | Yes | *** | 0.0001 |
| *B. pauloensis* vs. *B. leucurus* | -47.49 | -61.29 to -33.69 | Yes | **** | <0.0001 |
| *B. pauloensis* vs. *B. pictus* | 10.92 | 2.919 to 18.92 | Yes | ** | 0.0036 |
| *B. pauloensis* vs. *B. diporus* | -55.56 | -68.17 to -42.94 | Yes | **** | <0.0001 |
| *B. pauloensis* vs. *B. taeniatus* | 21.56 | 14.05 to 29.07 | Yes | **** | <0.0001 |
| *B. pauloensis* vs. *B. oligolepis* | 24.83 | 17.33 to 32.33 | Yes | **** | <0.0001 |
| *B. mattogrossensis* vs. *B. alternatus* | -0.7460 | -11.80 to 10.31 | No | ns | >0.9999 |
| *B. mattogrossensis* vs. *B. caribbaeus* | 2.866 | -6.244 to 11.98 | No | ns | 0.9997 |
| *B. mattogrossensis* vs. *B. lanceolatus* | 2.604 | -7.407 to 12.61 | No | ns | >0.9999 |
| *B. mattogrossensis* vs. *B. atrox* | -3.440 | -11.62 to 4.744 | No | ns | 0.9734 |
| *B. mattogrossensis* vs. *B. asper* | -35.27 | -51.49 to -19.04 | Yes | **** | <0.0001 |
| *B. mattogrossensis* vs. *B. leucurus* | -49.03 | -62.83 to -35.23 | Yes | **** | <0.0001 |
| *B. mattogrossensis* vs. *B. pictus* | 9.381 | 1.378 to 17.38 | Yes | * | 0.0142 |
| *B. mattogrossensis* vs. *B. diporus* | -57.09 | -69.71 to -44.47 | Yes | **** | <0.0001 |
| *B. mattogrossensis* vs. *B. taeniatus* | 20.02 | 12.51 to 27.54 | Yes | **** | <0.0001 |
| *B. mattogrossensis* vs. *B. oligolepis* | 23.29 | 15.78 to 30.80 | Yes | **** | <0.0001 |
| *B. alternatus* vs. *B. caribbaeus* | 3.613 | -7.418 to 14.64 | No | ns | 0.9992 |
| *B. alternatus* vs. *B. lanceolatus* | 3.350 | -8.322 to 15.02 | No | ns | >0.9999 |
| *B. alternatus* vs. *B. atrox* | -2.694 | -13.33 to 7.947 | No | ns | >0.9999 |
| *B. alternatus* vs. *B. asper* | -34.52 | -51.12 to -17.92 | Yes | **** | <0.0001 |
| *B. alternatus* vs. *B. leucurus* | -48.28 | -63.03 to -33.54 | Yes | **** | <0.0001 |
| *B. alternatus* vs. *B. pictus* | 10.13 | -0.2802 to 20.53 | No | ns | 0.0600 |
| *B. alternatus* vs. *B. diporus* | -56.35 | -69.90 to -42.79 | Yes | **** | <0.0001 |
| *B. alternatus* vs. *B. taeniatus* | 20.77 | 10.48 to 31.07 | Yes | *** | 0.0003 |
| *B. alternatus* vs. *B. oligolepis* | 24.04 | 13.75 to 34.33 | Yes | *** | 0.0001 |
| *B. caribbaeus* vs. *B. lanceolatus* | -0.2626 | -10.25 to 9.724 | No | ns | >0.9999 |
| *B. caribbaeus* vs. *B. atrox* | -6.306 | -14.46 to 1.847 | No | ns | 0.2484 |
| *B. caribbaeus* vs. *B. asper* | -38.13 | -54.34 to -21.93 | Yes | **** | <0.0001 |
| *B. caribbaeus* vs. *B. leucurus* | -51.89 | -65.91 to -37.88 | Yes | **** | <0.0001 |
| *B. caribbaeus* vs. *B. pictus* | 6.515 | -1.457 to 14.49 | No | ns | 0.1807 |
| *B. caribbaeus* vs. *B. diporus* | -59.96 | -72.56 to -47.36 | Yes | **** | <0.0001 |
| *B. caribbaeus* vs. *B. taeniatus* | 17.16 | 9.684 to 24.63 | Yes | **** | <0.0001 |
| *B. caribbaeus* vs. *B. oligolepis* | 20.42 | 12.96 to 27.89 | Yes | **** | <0.0001 |
| *B. lanceolatus* vs. *B. atrox* | -6.044 | -15.33 to 3.241 | No | ns | 0.4872 |
| *B. lanceolatus* vs. *B. asper* | -37.87 | -54.08 to -21.66 | Yes | **** | <0.0001 |
| *B. lanceolatus* vs. *B. leucurus* | -51.63 | -65.87 to -37.40 | Yes | **** | <0.0001 |
| *B. lanceolatus* vs. *B. pictus* | 6.777 | -2.386 to 15.94 | No | ns | 0.2853 |
| *B. lanceolatus* vs. *B. diporus* | -59.70 | -72.66 to -46.73 | Yes | **** | <0.0001 |
| *B. lanceolatus* vs. *B. taeniatus* | 17.42 | 8.410 to 26.43 | Yes | *** | 0.0005 |
| *B. lanceolatus* vs. *B. oligolepis* | 20.69 | 11.68 to 29.69 | Yes | *** | 0.0001 |
| *B. atrox* vs. *B. asper* | -31.83 | -47.76 to -15.89 | Yes | *** | 0.0002 |
| *B. atrox* vs. *B. leucurus* | -45.59 | -59.15 to -32.03 | Yes | **** | <0.0001 |
| *B. atrox* vs. *B. pictus* | 12.82 | 6.253 to 19.39 | Yes | **** | <0.0001 |
| *B. atrox* vs. *B. diporus* | -53.65 | -65.91 to -41.39 | Yes | **** | <0.0001 |
| *B. atrox* vs. *B. taeniatus* | 23.46 | 17.72 to 29.21 | Yes | **** | <0.0001 |
| *B. atrox* vs. *B. oligolepis* | 26.73 | 20.99 to 32.47 | Yes | **** | <0.0001 |
| *B. asper* vs. *B. leucurus* | -13.76 | -31.65 to 4.128 | No | ns | 0.2630 |
| *B. asper* vs. *B. pictus* | 44.65 | 28.88 to 60.41 | Yes | **** | <0.0001 |
| *B. asper* vs. *B. diporus* | -21.83 | -39.08 to -4.576 | Yes | ** | 0.0068 |
| *B. asper* vs. *B. taeniatus* | 55.29 | 39.12 to 71.46 | Yes | **** | <0.0001 |
| *B. asper* vs. *B. oligolepis* | 58.56 | 42.39 to 74.73 | Yes | **** | <0.0001 |
| *B. leucurus* vs. *B. pictus* | 58.41 | 44.72 to 72.10 | Yes | **** | <0.0001 |
| *B. leucurus* vs. *B. diporus* | -8.064 | -23.58 to 7.453 | No | ns | 0.8484 |
| *B. leucurus* vs. *B. taeniatus* | 69.05 | 55.66 to 82.44 | Yes | **** | <0.0001 |
| *B. leucurus* vs. *B. oligolepis* | 72.32 | 58.93 to 85.71 | Yes | **** | <0.0001 |
| *B. pictus* vs. *B. diporus* | -66.47 | -78.52 to -54.42 | Yes | **** | <0.0001 |
| *B. pictus* vs. *B. taeniatus* | 10.64 | 5.459 to 15.83 | Yes | **** | <0.0001 |
| *B. pictus* vs. *B. oligolepis* | 13.91 | 8.732 to 19.09 | Yes | **** | <0.0001 |
| *B. diporus* vs. *B. taeniatus* | 77.12 | 65.19 to 89.04 | Yes | **** | <0.0001 |
| *B. diporus* vs. *B. oligolepis* | 80.38 | 68.46 to 92.30 | Yes | **** | <0.0001 |
| *B. taeniatus* vs. *B. oligolepis* | 3.267 | 0.1995 to 6.335 | Yes | * | 0.0303 |

**HEK293 ratiometric response – comparing normalised AUC values at 0.009 mg/ml venom concentration**

**
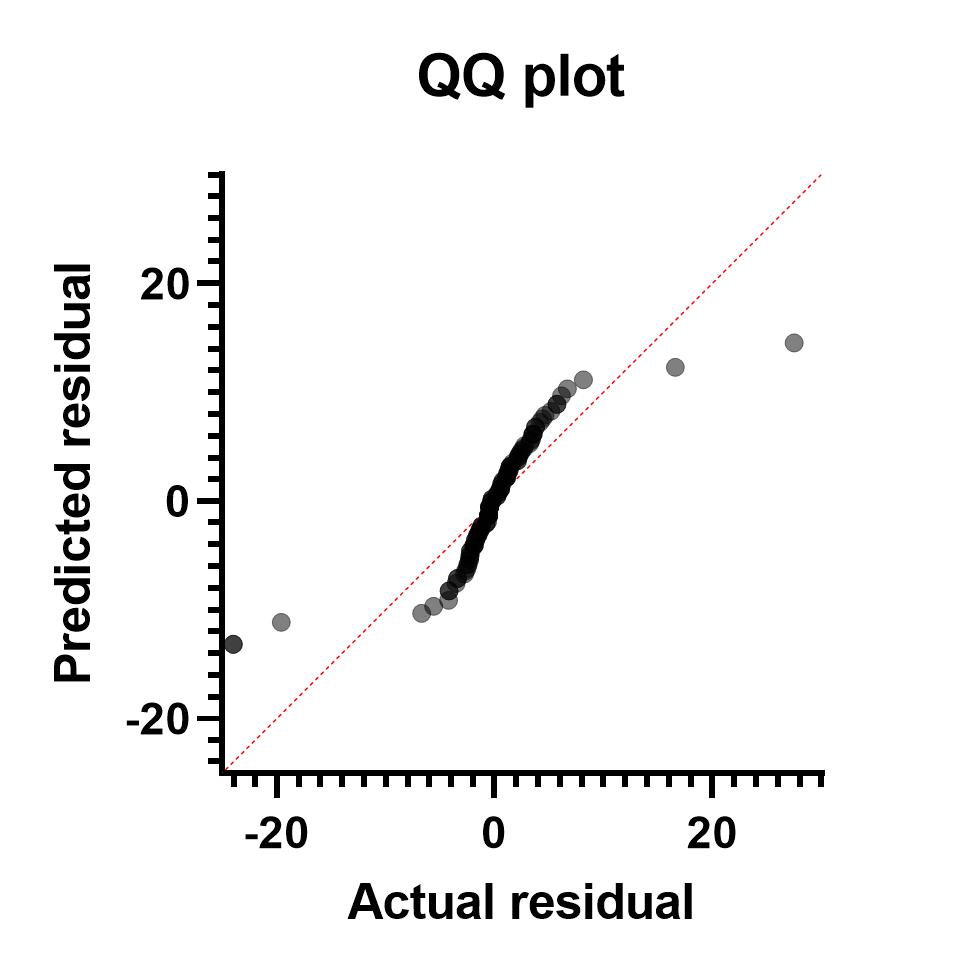
**

**Figure 9. QQ plot.**

**
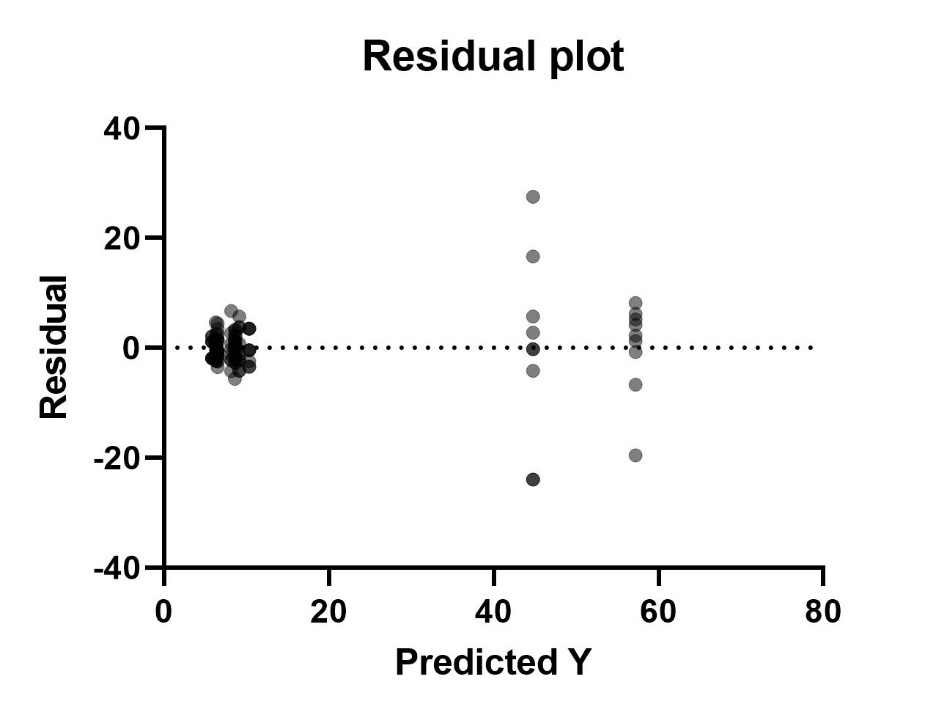
**

**Figure 10. Residual plot.**

**Table 9. Brown-Forsythe and Welch ANOVA tests.**

| Brown-Forsythe ANOVA test |  |
| --- | --- |
| F* (DFn, DFd) | 76.03 (11.00, 16.49) |
| P value | <0.0001 |
| P value summary | **** |
| Significant diff. among means (P < 0.05)? | Yes |
|  |  |
| Welch's ANOVA test |  |
| W (DFn, DFd) | 29.77 (11.00, 37.13) |
| P value | <0.0001 |
| P value summary | **** |
| Significant diff. among means (P < 0.05)? | Yes |

**Table 10. Dunnets’s T3 multiple comparisons test.**

| Dunnett's T3 multiple comparisons test | Mean Diff. | 95.00% CI of diff. | Summary | Adjusted P Value |
| --- | --- | --- | --- | --- |
| *B. pauloensis* vs. *B. mattogrossensis* | -12.43 | -38.89 to 14.03 | ns | 0.8791 |
| *B. pauloensis* vs. *B. alternatus* | 38.50 | 12.09 to 64.92 | ** | 0.0049 |
| *B. pauloensis* vs. *B. caribbaeus* | 38.28 | 11.99 to 64.58 | ** | 0.0050 |
| *B. pauloensis* vs. *B. lanceolatus* | 36.61 | 10.72 to 62.49 | ** | 0.0051 |
| *B. pauloensis* vs. *B. atrox* | 38.94 | 12.60 to 65.29 | ** | 0.0045 |
| *B. pauloensis* vs. *B. asper* | 38.39 | 12.11 to 64.68 | ** | 0.0049 |
| *B. pauloensis* vs. *B. leucurus* | 38.28 | 11.78 to 64.78 | ** | 0.0052 |
| *B. pauloensis* vs. *B. pictus* | 34.43 | 7.855 to 61.01 | * | 0.0107 |
| *B. pauloensis* vs. *B. diporus* | 36.19 | 9.661 to 62.73 | ** | 0.0077 |
| *B. pauloensis* vs. *B. taeniatus* | 35.64 | 9.749 to 61.54 | ** | 0.0062 |
| *B. pauloensis* vs. *B. oligolepis* | 36.08 | 9.713 to 62.45 | ** | 0.0075 |
| *B. mattogrossensis* vs. *B. alternatus* | 50.94 | 37.60 to 64.27 | **** | <0.0001 |
| *B. mattogrossensis* vs. *B. caribbaeus* | 50.72 | 37.14 to 64.29 | **** | <0.0001 |
| *B. mattogrossensis* vs. *B. lanceolatus* | 49.04 | 35.71 to 62.36 | **** | <0.0001 |
| *B. mattogrossensis* vs. *B. atrox* | 51.38 | 38.17 to 64.58 | **** | <0.0001 |
| *B. mattogrossensis* vs. *B. asper* | 50.83 | 37.27 to 64.38 | **** | <0.0001 |
| *B. mattogrossensis* vs. *B. leucurus* | 50.72 | 37.23 to 64.20 | **** | <0.0001 |
| *B. mattogrossensis* vs. *B. pictus* | 46.86 | 33.61 to 60.12 | **** | <0.0001 |
| *B. mattogrossensis* vs. *B. diporus* | 48.62 | 35.45 to 61.80 | **** | <0.0001 |
| *B. mattogrossensis* vs. *B. taeniatus* | 48.07 | 34.73 to 61.42 | **** | <0.0001 |
| *B. mattogrossensis* vs. *B. oligolepis* | 48.51 | 35.26 to 61.76 | **** | <0.0001 |
| *B. alternatus* vs. *B. caribbaeus* | -0.2200 | -3.867 to 3.427 | ns | >0.9999 |
| *B. alternatus* vs. *B. lanceolatus* | -1.898 | -7.882 to 4.086 | ns | 0.9983 |
| *B. alternatus* vs. *B. atrox* | 0.4400 | -3.420 to 4.300 | ns | >0.9999 |
| *B. alternatus* vs. *B. asper* | -0.1100 | -3.694 to 3.474 | ns | >0.9999 |
| *B. alternatus* vs. *B. leucurus* | -0.2200 | -4.751 to 4.311 | ns | >0.9999 |
| *B. alternatus* vs. *B. pictus* | -4.070 | -8.981 to 0.8397 | ns | 0.1717 |
| *B. alternatus* vs. *B. diporus* | -2.310 | -7.030 to 2.410 | ns | 0.8916 |
| *B. alternatus* vs. *B. taeniatus* | -2.860 | -8.790 to 3.069 | ns | 0.8744 |
| *B. alternatus* vs. *B. oligolepis* | -2.420 | -6.369 to 1.529 | ns | 0.6202 |
| *B. caribbaeus* vs. *B. lanceolatus* | -1.678 | -7.607 to 4.252 | ns | 0.9982 |
| *B. caribbaeus* vs. *B. atrox* | 0.6601 | -2.510 to 3.830 | ns | >0.9999 |
| *B. caribbaeus* vs. *B. asper* | 0.1100 | -2.630 to 2.850 | ns | >0.9999 |
| *B. caribbaeus* vs. *B. leucurus* | 0.000 | -4.127 to 4.127 | ns | >0.9999 |
| *B. caribbaeus* vs. *B. pictus* | -3.850 | -8.434 to 0.7330 | ns | 0.1456 |
| *B. caribbaeus* vs. *B. diporus* | -2.090 | -6.450 to 2.270 | ns | 0.8612 |
| *B. caribbaeus* vs. *B. taeniatus* | -2.640 | -8.322 to 3.042 | ns | 0.8675 |
| *B. caribbaeus* vs. *B. oligolepis* | -2.200 | -5.530 to 1.130 | ns | 0.4800 |
| *B. lanceolatus* vs. *B. atrox* | 2.338 | -3.623 to 8.298 | ns | 0.9544 |
| *B. lanceolatus* vs. *B. asper* | 1.788 | -4.094 to 7.670 | ns | 0.9953 |
| *B. lanceolatus* vs. *B. leucurus* | 1.678 | -4.485 to 7.841 | ns | >0.9999 |
| *B. lanceolatus* vs. *B. pictus* | -2.173 | -8.506 to 4.160 | ns | 0.9975 |
| *B. lanceolatus* vs. *B. diporus* | -0.4125 | -6.685 to 5.860 | ns | >0.9999 |
| *B. lanceolatus* vs. *B. taeniatus* | -0.9626 | -7.914 to 5.989 | ns | >0.9999 |
| *B. lanceolatus* vs. *B. oligolepis* | -0.5226 | -6.447 to 5.402 | ns | >0.9999 |
| *B. atrox* vs. *B. asper* | -0.5501 | -3.649 to 2.549 | ns | >0.9999 |
| *B. atrox* vs. *B. leucurus* | -0.6601 | -4.964 to 3.644 | ns | >0.9999 |
| *B. atrox* vs. *B. pictus* | -4.510 | -9.239 to 0.2180 | ns | 0.0691 |
| *B. atrox* vs. *B. diporus* | -2.750 | -7.205 to 1.705 | ns | 0.5723 |
| *B. atrox* vs. *B. taeniatus* | -3.300 | -9.059 to 2.458 | ns | 0.6409 |
| *B. atrox* vs. *B. oligolepis* | -2.860 | -6.429 to 0.7085 | ns | 0.2127 |
| *B. asper* vs. *B. leucurus* | -0.1100 | -4.248 to 4.027 | ns | >0.9999 |
| *B. asper* vs. *B. pictus* | -3.960 | -8.490 to 0.5696 | ns | 0.1164 |
| *B. asper* vs. *B. diporus* | -2.200 | -6.504 to 2.104 | ns | 0.7939 |
| *B. asper* vs. *B. taeniatus* | -2.750 | -8.520 to 3.019 | ns | 0.8164 |
| *B. asper* vs. *B. oligolepis* | -2.310 | -5.573 to 0.9523 | ns | 0.3705 |
| *B. leucurus* vs. *B. pictus* | -3.850 | -9.016 to 1.315 | ns | 0.3059 |
| *B. leucurus* vs. *B. diporus* | -2.090 | -7.079 to 2.899 | ns | 0.9788 |
| *B. leucurus* vs. *B. taeniatus* | -2.640 | -8.757 to 3.476 | ns | 0.9577 |
| *B. leucurus* vs. *B. oligolepis* | -2.200 | -6.572 to 2.171 | ns | 0.8667 |
| *B. pictus* vs. *B. diporus* | 1.760 | -3.526 to 7.046 | ns | 0.9992 |
| *B. pictus* vs. *B. taeniatus* | 1.210 | -5.083 to 7.503 | ns | >0.9999 |
| *B. pictus* vs. *B. oligolepis* | 1.650 | -3.124 to 6.424 | ns | 0.9972 |
| *B. diporus* vs. *B. taeniatus* | -0.5501 | -6.696 to 5.596 | ns | >0.9999 |
| *B. diporus* vs. *B. oligolepis* | -0.1100 | -4.626 to 4.406 | ns | >0.9999 |
| *B. taeniatus* vs. *B. oligolepis* | 0.4400 | -5.416 to 6.296 | ns | >0.9999 |

**SHSY5Y ratiometric response – comparing normalised AUC values at 0.009 mg/ml venom concentration**

**
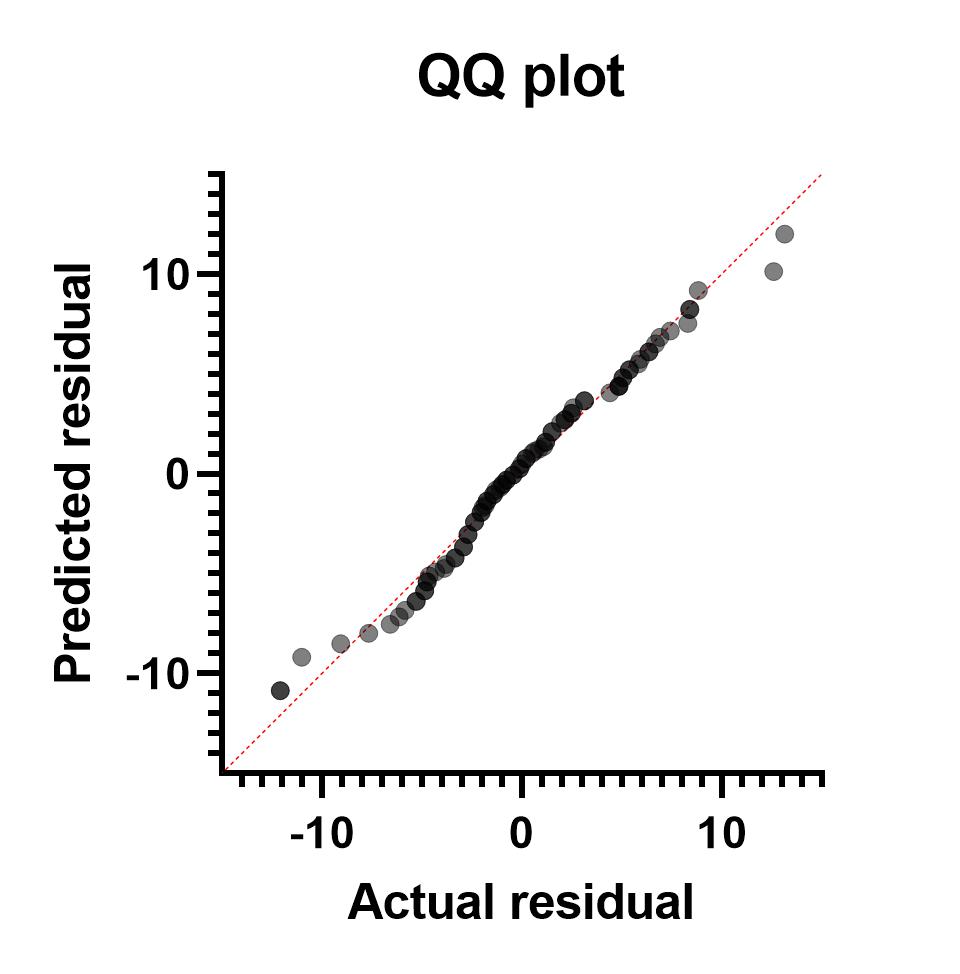
**

**Figure 11. QQ plot.**

**
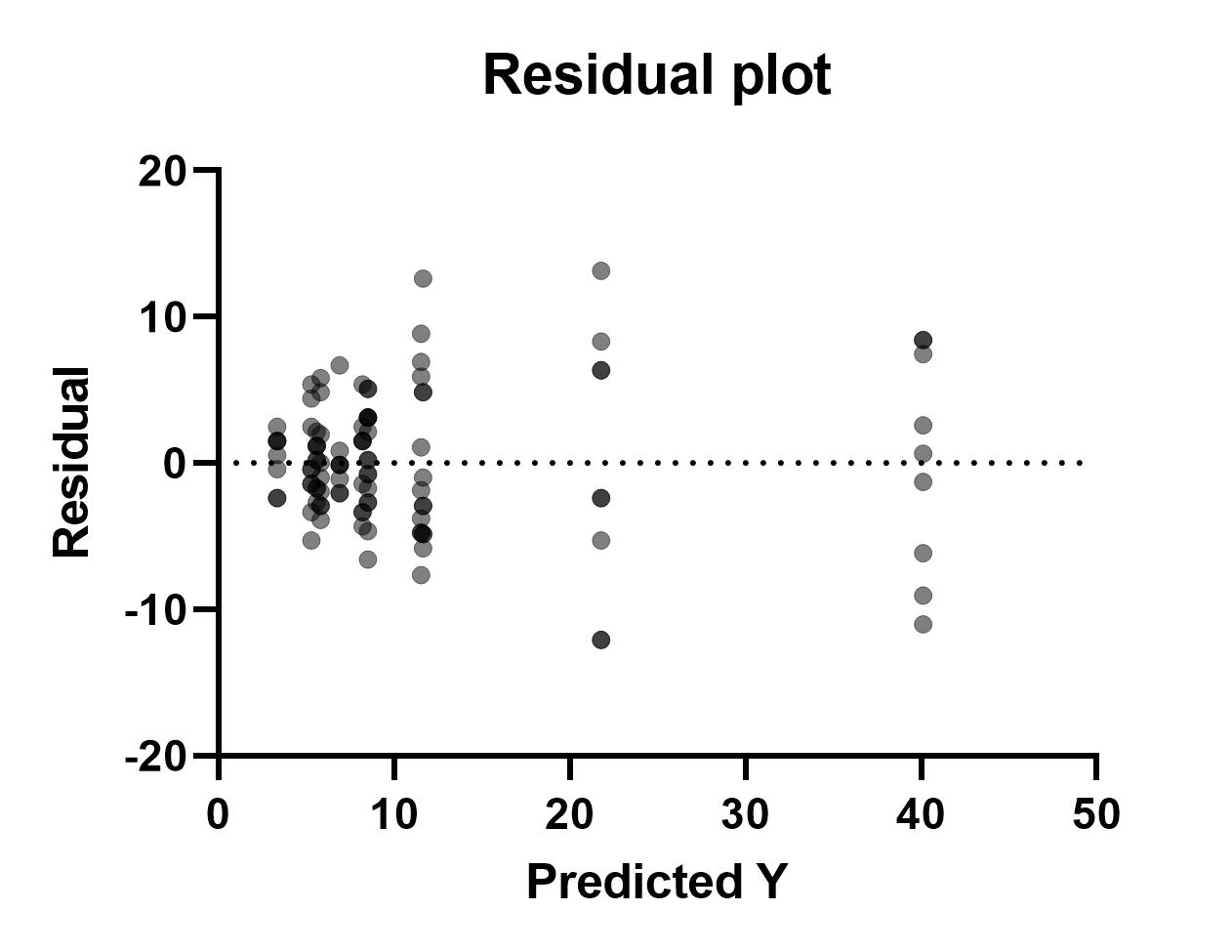
**

**Figure 12. Residual plot.**

**Table 11. Brown-Forsythe and Welch ANOVA tests.**

| Brown-Forsythe ANOVA test |  |
| --- | --- |
| F* (DFn, DFd) | 39.58 (11.00, 48.36) |
| P value | <0.0001 |
| P value summary | **** |
| Significant diff. among means (P < 0.05)? | Yes |
|  |  |
| Welch's ANOVA test |  |
| W (DFn, DFd) | 19.94 (11.00, 37.55) |
| P value | <0.0001 |
| P value summary | **** |
| Significant diff. among means (P < 0.05)? | Yes |

**Table 12. Dunnets’s T3 multiple comparisons test.**

| Dunnett's T3 multiple comparisons test | Mean Diff. | 95.00% CI of diff. | Summary | Adjusted P Value |
| --- | --- | --- | --- | --- |
| *B. pauloensis* vs. *B. mattogrossensis* | -18.34 | -34.16 to -2.520 | * | 0.0151 |
| *B. pauloensis* vs. *B. alternatus* | 18.45 | 4.463 to 32.43 | ** | 0.0083 |
| *B. pauloensis* vs. *B. caribbaeus* | 16.50 | 2.250 to 30.76 | * | 0.0190 |
| *B. pauloensis* vs. *B. lanceolatus* | 15.97 | 1.713 to 30.22 | * | 0.0239 |
| *B. pauloensis* vs. *B. atrox* | 13.27 | -0.8432 to 27.38 | ns | 0.0711 |
| *B. pauloensis* vs. *B. asper* | 13.59 | -0.5369 to 27.72 | ns | 0.0633 |
| *B. pauloensis* vs. *B. leucurus* | 13.27 | -1.134 to 27.67 | ns | 0.0834 |
| *B. pauloensis* vs. *B. pictus* | 10.25 | -4.536 to 25.03 | ns | 0.3891 |
| *B. pauloensis* vs. *B. diporus* | 10.14 | -4.814 to 25.09 | ns | 0.4228 |
| *B. pauloensis* vs. *B. taeniatus* | 14.89 | 0.6105 to 29.16 | * | 0.0389 |
| *B. pauloensis* vs. *B. oligolepis* | 16.18 | 2.281 to 30.08 | * | 0.0195 |
| *B. mattogrossensis* vs. *B. alternatus* | 36.79 | 25.11 to 48.46 | **** | <0.0001 |
| *B. mattogrossensis* vs. *B. caribbaeus* | 34.84 | 22.99 to 46.70 | **** | <0.0001 |
| *B. mattogrossensis* vs. *B. lanceolatus* | 34.30 | 22.45 to 46.15 | **** | <0.0001 |
| *B. mattogrossensis* vs. *B. atrox* | 31.61 | 20.11 to 43.11 | **** | <0.0001 |
| *B. mattogrossensis* vs. *B. asper* | 31.93 | 20.22 to 43.64 | **** | <0.0001 |
| *B. mattogrossensis* vs. *B. leucurus* | 31.61 | 19.64 to 43.57 | **** | <0.0001 |
| *B. mattogrossensis* vs. *B. pictus* | 28.59 | 15.70 to 41.47 | **** | <0.0001 |
| *B. mattogrossensis* vs. *B. diporus* | 28.48 | 15.40 to 41.55 | **** | <0.0001 |
| *B. mattogrossensis* vs. *B. taeniatus* | 33.23 | 21.54 to 44.91 | **** | <0.0001 |
| *B. mattogrossensis* vs. *B. oligolepis* | 34.52 | 22.95 to 46.09 | **** | <0.0001 |
| *B. alternatus* vs. *B. caribbaeus* | -1.942 | -7.495 to 3.612 | ns | 0.9953 |
| *B. alternatus* vs. *B. lanceolatus* | -2.481 | -8.030 to 3.068 | ns | 0.9301 |
| *B. alternatus* vs. *B. atrox* | -5.178 | -9.215 to -1.141 | ** | 0.0056 |
| *B. alternatus* vs. *B. asper* | -4.854 | -10.12 to 0.4064 | ns | 0.0857 |
| *B. alternatus* vs. *B. leucurus* | -5.178 | -12.00 to 1.648 | ns | 0.2323 |
| *B. alternatus* vs. *B. pictus* | -8.198 | -17.41 to 1.009 | ns | 0.0988 |
| *B. alternatus* vs. *B. diporus* | -8.306 | -17.83 to 1.215 | ns | 0.1104 |
| *B. alternatus* vs. *B. taeniatus* | -3.560 | -8.130 to 1.011 | ns | 0.2321 |
| *B. alternatus* vs. *B. oligolepis* | -2.265 | -5.684 to 1.153 | ns | 0.4904 |
| *B. caribbaeus* vs. *B. lanceolatus* | -0.5394 | -7.145 to 6.066 | ns | >0.9999 |
| *B. caribbaeus* vs. *B. atrox* | -3.236 | -8.972 to 2.499 | ns | 0.7103 |
| *B. caribbaeus* vs. *B. asper* | -2.913 | -9.296 to 3.471 | ns | 0.9480 |
| *B. caribbaeus* vs. *B. leucurus* | -3.236 | -10.75 to 4.281 | ns | 0.9657 |
| *B. caribbaeus* vs. *B. pictus* | -6.257 | -15.83 to 3.312 | ns | 0.4616 |
| *B. caribbaeus* vs. *B. diporus* | -6.365 | -16.20 to 3.474 | ns | 0.4782 |
| *B. caribbaeus* vs. *B. taeniatus* | -1.618 | -7.601 to 4.365 | ns | >0.9999 |
| *B. caribbaeus* vs. *B. oligolepis* | -0.3236 | -5.895 to 5.248 | ns | >0.9999 |
| *B. lanceolatus* vs. *B. atrox* | -2.697 | -8.428 to 3.034 | ns | 0.9094 |
| *B. lanceolatus* vs. *B. asper* | -2.373 | -8.753 to 4.006 | ns | 0.9954 |
| *B. lanceolatus* vs. *B. leucurus* | -2.697 | -10.21 to 4.817 | ns | 0.9964 |
| *B. lanceolatus* vs. *B. pictus* | -5.717 | -15.28 to 3.849 | ns | 0.6021 |
| *B. lanceolatus* vs. *B. diporus* | -5.825 | -15.66 to 4.010 | ns | 0.6161 |
| *B. lanceolatus* vs. *B. taeniatus* | -1.079 | -7.058 to 4.900 | ns | >0.9999 |
| *B. lanceolatus* vs. *B. oligolepis* | 0.2157 | -5.351 to 5.783 | ns | >0.9999 |
| *B. atrox* vs. *B. asper* | 0.3236 | -5.137 to 5.785 | ns | >0.9999 |
| *B. atrox* vs. *B. leucurus* | 0.000 | -6.923 to 6.923 | ns | >0.9999 |
| *B. atrox* vs. *B. pictus* | -3.020 | -12.41 to 6.369 | ns | 0.9945 |
| *B. atrox* vs. *B. diporus* | -3.128 | -12.83 to 6.569 | ns | 0.9943 |
| *B. atrox* vs. *B. taeniatus* | 1.618 | -3.149 to 6.385 | ns | 0.9989 |
| *B. atrox* vs. *B. oligolepis* | 2.913 | -0.9350 to 6.760 | ns | 0.2758 |
| *B. asper* vs. *B. leucurus* | -0.3236 | -7.638 to 6.991 | ns | >0.9999 |
| *B. asper* vs. *B. pictus* | -3.344 | -12.90 to 6.216 | ns | 0.9932 |
| *B. asper* vs. *B. diporus* | -3.452 | -13.29 to 6.386 | ns | 0.9930 |
| *B. asper* vs. *B. taeniatus* | 1.294 | -4.370 to 6.959 | ns | >0.9999 |
| *B. asper* vs. *B. oligolepis* | 2.589 | -2.567 to 7.745 | ns | 0.8143 |
| *B. leucurus* vs. *B. pictus* | -3.020 | -12.95 to 6.911 | ns | 0.9998 |
| *B. leucurus* vs. *B. diporus* | -3.128 | -13.43 to 7.177 | ns | 0.9997 |
| *B. leucurus* vs. *B. taeniatus* | 1.618 | -5.473 to 8.709 | ns | >0.9999 |
| *B. leucurus* vs. *B. oligolepis* | 2.913 | -3.914 to 9.739 | ns | 0.9103 |
| *B. pictus* vs. *B. diporus* | -0.1079 | -11.55 to 11.34 | ns | >0.9999 |
| *B. pictus* vs. *B. taeniatus* | 4.639 | -4.763 to 14.04 | ns | 0.8084 |
| *B. pictus* vs. *B. oligolepis* | 5.933 | -3.412 to 15.28 | ns | 0.4063 |
| *B. diporus* vs. *B. taeniatus* | 4.746 | -4.949 to 14.44 | ns | 0.8169 |
| *B. diporus* vs. *B. oligolepis* | 6.041 | -3.632 to 15.71 | ns | 0.4279 |
| *B. taeniatus* vs. *B. oligolepis* | 1.294 | -3.126 to 5.715 | ns | 0.9997 |

**SHSY5Y ratiometric response – comparing normalised AUC values at 6.66 mg/ml venom concentration**

**
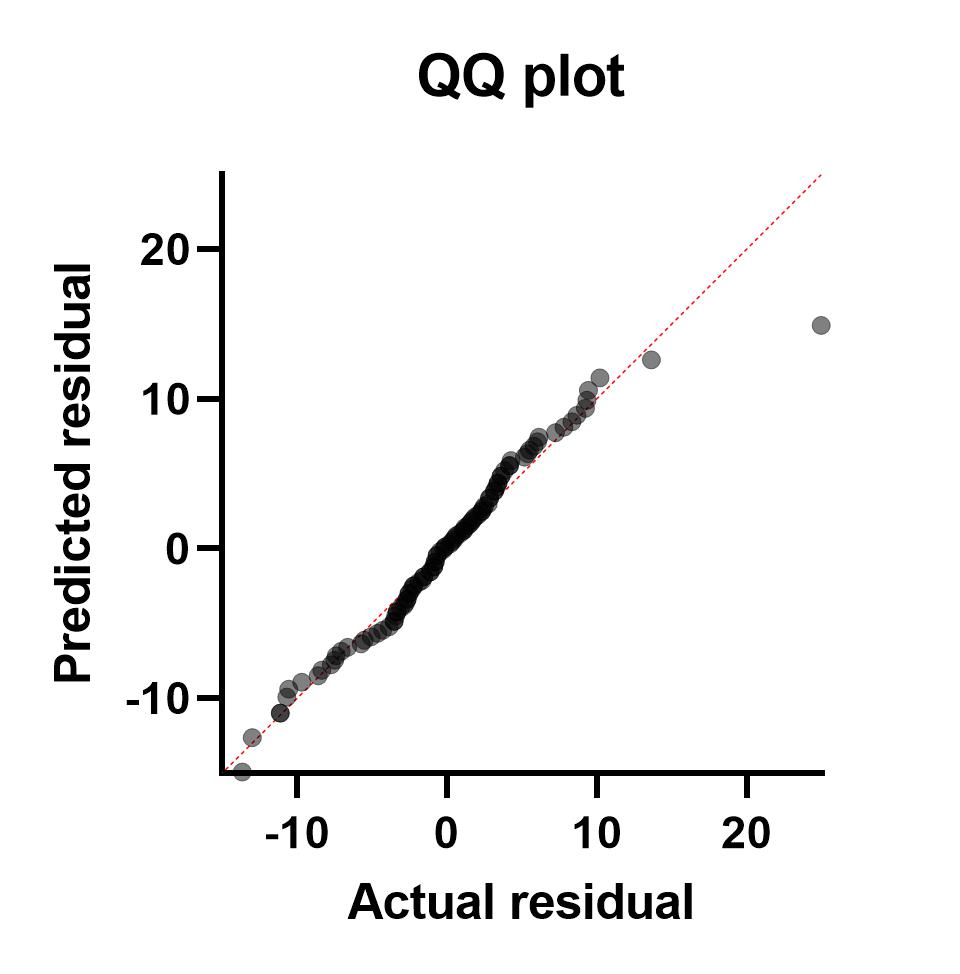
**

**Figure 12. QQ plot.**

**
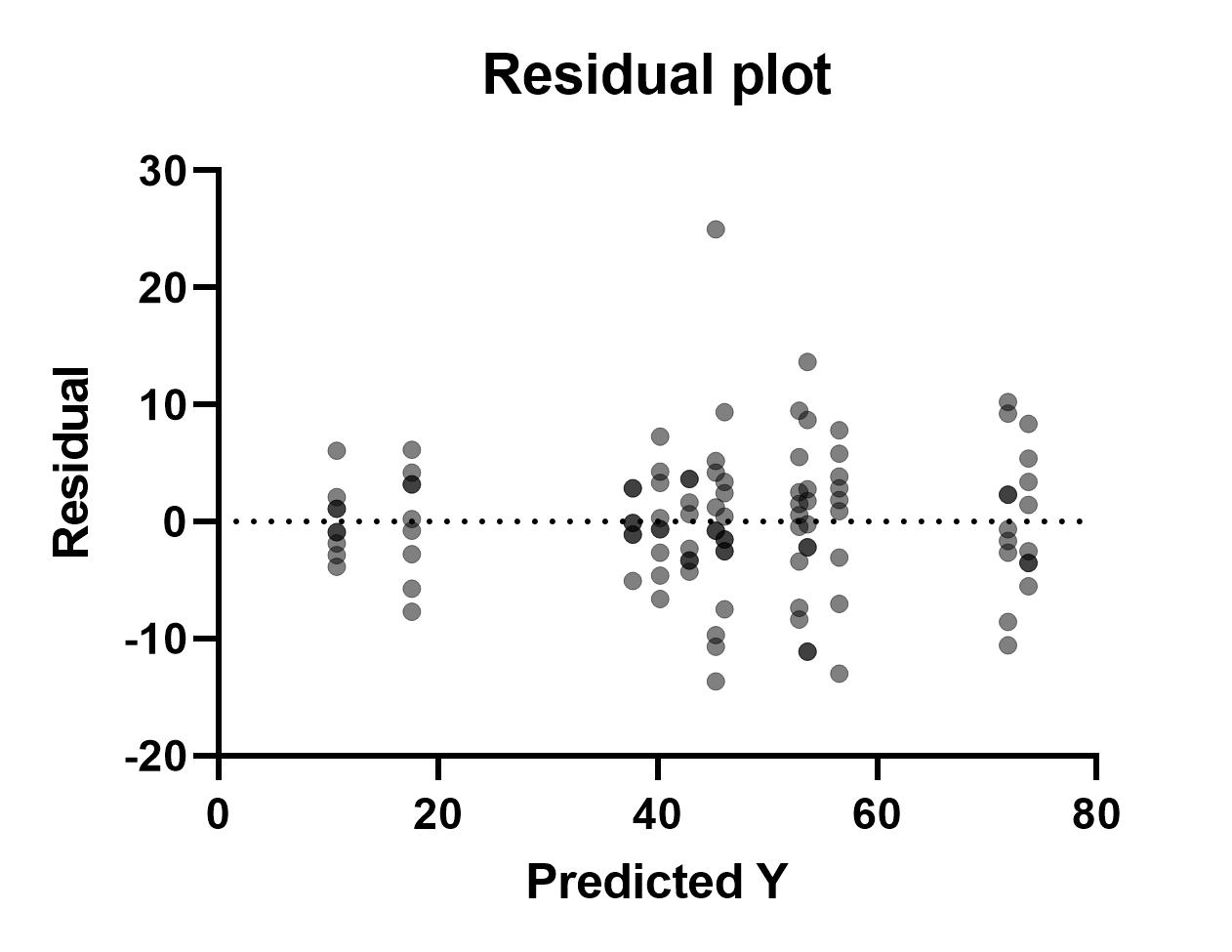
**

**Figure 13. Residual plot.**

**Table 12. Brown-Forsythe and Welch ANOVA tests.**

| Brown-Forsythe ANOVA test |  |
| --- | --- |
| F* (DFn, DFd) | 85.46 (11.00, 52.00) |
| P value | <0.0001 |
| P value summary | **** |
| Significant diff. among means (P < 0.05)? | Yes |
|  |  |
| Welch's ANOVA test |  |
| W (DFn, DFd) | 141.9 (11.00, 37.63) |
| P value | <0.0001 |
| P value summary | **** |
| Significant diff. among means (P < 0.05)? | Yes |

**Table 13. Dunnets’s T3 multiple comparisons test.**

| Dunnett's T3 multiple comparisons test | Mean Diff. | 95.00% CI of diff. | Summary | Adjusted P Value |
| --- | --- | --- | --- | --- |
| *B. pauloensis* vs. *B. mattogrossensis* | -1.870 | -13.52 to 9.780 | ns | >0.9999 |
| *B. pauloensis* vs. *B. alternatus* | 25.85 | 14.28 to 37.42 | **** | <0.0001 |
| *B. pauloensis* vs. *B. caribbaeus* | 18.26 | 3.922 to 32.60 | ** | 0.0060 |
| *B. pauloensis* vs. *B. lanceolatus* | 26.62 | 7.910 to 45.33 | ** | 0.0027 |
| *B. pauloensis* vs. *B. atrox* | 31.68 | 20.19 to 43.18 | **** | <0.0001 |
| *B. pauloensis* vs. *B. asper* | 19.03 | 6.776 to 31.29 | *** | 0.0008 |
| *B. pauloensis* vs. *B. leucurus* | 29.04 | 17.87 to 40.22 | **** | <0.0001 |
| *B. pauloensis* vs. *B. pictus* | 34.21 | 23.19 to 45.23 | **** | <0.0001 |
| *B. pauloensis* vs. *B. diporus* | 15.40 | 2.525 to 28.28 | * | 0.0109 |
| *B. pauloensis* vs. *B. taeniatus* | 54.35 | 42.80 to 65.89 | **** | <0.0001 |
| *B. pauloensis* vs. *B. oligolepis* | 61.17 | 50.19 to 72.14 | **** | <0.0001 |
| *B. mattogrossensis* vs. *B. alternatus* | 27.72 | 18.71 to 36.73 | **** | <0.0001 |
| *B. mattogrossensis* vs. *B. caribbaeus* | 20.13 | 7.034 to 33.23 | ** | 0.0013 |
| *B. mattogrossensis* vs. *B. lanceolatus* | 28.49 | 10.54 to 46.45 | ** | 0.0014 |
| *B. mattogrossensis* vs. *B. atrox* | 33.55 | 24.84 to 42.26 | **** | <0.0001 |
| *B. mattogrossensis* vs. *B. asper* | 20.90 | 10.88 to 30.92 | **** | <0.0001 |
| *B. mattogrossensis* vs. *B. leucurus* | 30.91 | 22.91 to 38.91 | **** | <0.0001 |
| *B. mattogrossensis* vs. *B. pictus* | 36.08 | 28.37 to 43.80 | **** | <0.0001 |
| *B. mattogrossensis* vs. *B. diporus* | 17.27 | 6.232 to 28.31 | *** | 0.0008 |
| *B. mattogrossensis* vs. *B. taeniatus* | 56.22 | 47.24 to 65.19 | **** | <0.0001 |
| *B. mattogrossensis* vs. *B. oligolepis* | 63.04 | 55.17 to 70.90 | **** | <0.0001 |
| *B. alternatus* vs. *B. caribbaeus* | -7.591 | -20.62 to 5.435 | ns | 0.6407 |
| *B. alternatus* vs. *B. lanceolatus* | 0.7701 | -17.13 to 18.67 | ns | >0.9999 |
| *B. alternatus* vs. *B. atrox* | 5.831 | -2.780 to 14.44 | ns | 0.4545 |
| *B. alternatus* vs. *B. asper* | -6.821 | -16.86 to 3.219 | ns | 0.4351 |
| *B. alternatus* vs. *B. leucurus* | 3.190 | -4.695 to 11.08 | ns | 0.9784 |
| *B. alternatus* vs. *B. pictus* | 8.361 | 0.7700 to 15.95 | * | 0.0252 |
| *B. alternatus* vs. *B. diporus* | -10.45 | -21.55 to 0.6460 | ns | 0.0761 |
| *B. alternatus* vs. *B. taeniatus* | 28.49 | 19.61 to 37.37 | **** | <0.0001 |
| *B. alternatus* vs. *B. oligolepis* | 35.31 | 27.68 to 42.95 | **** | <0.0001 |
| *B. caribbaeus* vs. *B. lanceolatus* | 8.361 | -10.91 to 27.63 | ns | 0.9555 |
| *B. caribbaeus* vs. *B. atrox* | 13.42 | 0.4015 to 26.44 | * | 0.0406 |
| *B. caribbaeus* vs. *B. asper* | 0.7701 | -12.83 to 14.37 | ns | >0.9999 |
| *B. caribbaeus* vs. *B. leucurus* | 10.78 | -1.831 to 23.39 | ns | 0.1278 |
| *B. caribbaeus* vs. *B. pictus* | 15.95 | 3.408 to 28.49 | ** | 0.0097 |
| *B. caribbaeus* vs. *B. diporus* | -2.860 | -16.99 to 11.27 | ns | >0.9999 |
| *B. caribbaeus* vs. *B. taeniatus* | 36.08 | 23.08 to 49.09 | **** | <0.0001 |
| *B. caribbaeus* vs. *B. oligolepis* | 42.90 | 30.17 to 55.64 | **** | <0.0001 |
| *B. lanceolatus* vs. *B. atrox* | 5.061 | -13.08 to 23.20 | ns | 0.9993 |
| *B. lanceolatus* vs. *B. asper* | -7.591 | -25.74 to 10.56 | ns | 0.9524 |
| *B. lanceolatus* vs. *B. leucurus* | 2.420 | -15.72 to 20.56 | ns | >0.9999 |
| *B. lanceolatus* vs. *B. pictus* | 7.591 | -10.28 to 25.46 | ns | 0.8884 |
| *B. lanceolatus* vs. *B. diporus* | -11.22 | -29.64 to 7.193 | ns | 0.5719 |
| *B. lanceolatus* vs. *B. taeniatus* | 27.72 | 9.844 to 45.60 | ** | 0.0017 |
| *B. lanceolatus* vs. *B. oligolepis* | 34.54 | 16.53 to 52.55 | *** | 0.0005 |
| *B. atrox* vs. *B. asper* | -12.65 | -22.42 to -2.884 | ** | 0.0054 |
| *B. atrox* vs. *B. leucurus* | -2.640 | -10.07 to 4.793 | ns | 0.9969 |
| *B. atrox* vs. *B. pictus* | 2.530 | -4.544 to 9.604 | ns | 0.9936 |
| *B. atrox* vs. *B. diporus* | -16.28 | -27.13 to -5.437 | ** | 0.0014 |
| *B. atrox* vs. *B. taeniatus* | 22.66 | 14.09 to 31.24 | **** | <0.0001 |
| *B. atrox* vs. *B. oligolepis* | 29.48 | 22.22 to 36.75 | **** | <0.0001 |
| *B. asper* vs. *B. leucurus* | 10.01 | 0.8090 to 19.21 | * | 0.0269 |
| *B. asper* vs. *B. pictus* | 15.18 | 6.118 to 24.24 | *** | 0.0009 |
| *B. asper* vs. *B. diporus* | -3.630 | -15.32 to 8.060 | ns | 0.9998 |
| *B. asper* vs. *B. taeniatus* | 35.31 | 25.30 to 45.32 | **** | <0.0001 |
| *B. asper* vs. *B. oligolepis* | 42.13 | 33.00 to 51.27 | **** | <0.0001 |
| *B. leucurus* vs. *B. pictus* | 5.171 | -0.5109 to 10.85 | ns | 0.0969 |
| *B. leucurus* vs. *B. diporus* | -13.64 | -24.06 to -3.220 | ** | 0.0064 |
| *B. leucurus* vs. *B. taeniatus* | 25.30 | 17.46 to 33.15 | **** | <0.0001 |
| *B. leucurus* vs. *B. oligolepis* | 32.12 | 26.17 to 38.08 | **** | <0.0001 |
| *B. pictus* vs. *B. diporus* | -18.81 | -29.25 to -8.372 | *** | 0.0006 |
| *B. pictus* vs. *B. taeniatus* | 20.13 | 12.58 to 27.68 | **** | <0.0001 |
| *B. pictus* vs. *B. oligolepis* | 26.95 | 21.68 to 32.23 | **** | <0.0001 |
| *B. diporus* vs. *B. taeniatus* | 38.94 | 27.87 to 50.01 | **** | <0.0001 |
| *B. diporus* vs. *B. oligolepis* | 45.76 | 35.34 to 56.19 | **** | <0.0001 |
| *B. taeniatus* vs. *B. oligolepis* | 6.821 | -0.7730 to 14.41 | ns | 0.1036 |

**SHSY5Y ratiometric response – comparing normalised AUC values at 6.66 mg/ml venom concentration**

**
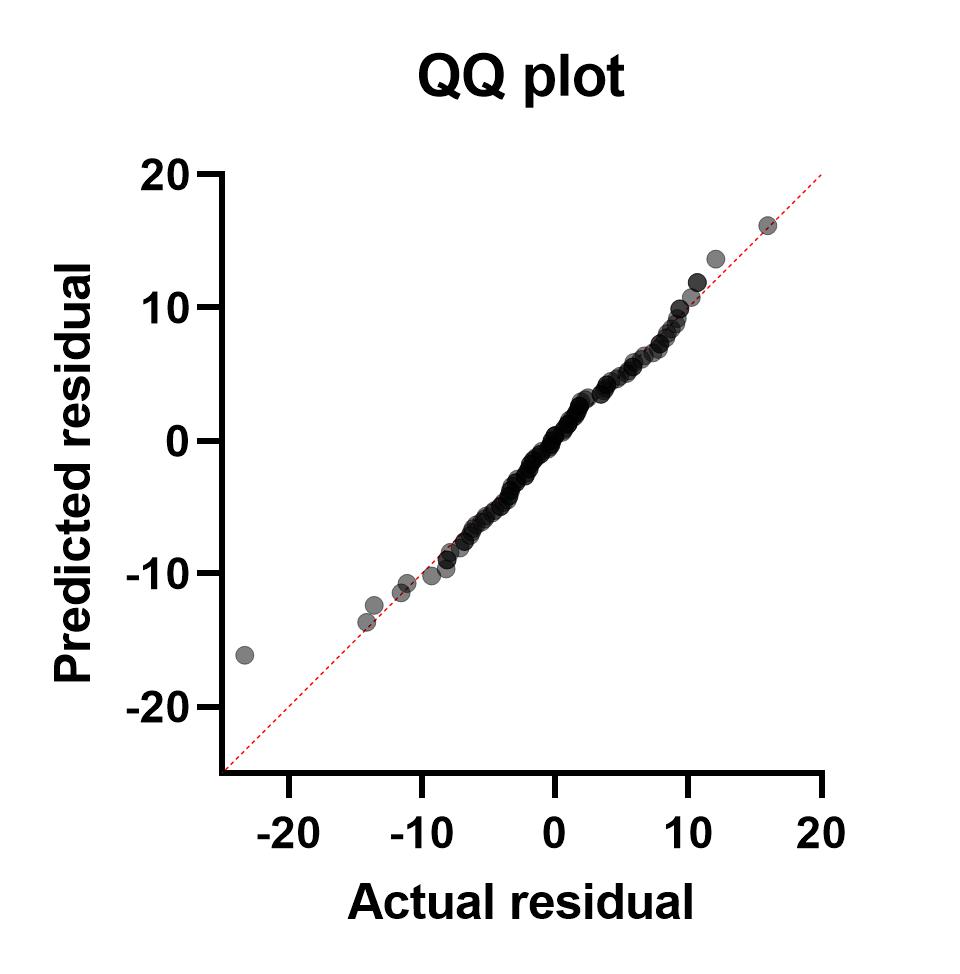
**

**Figure 14. QQ plot.**

**
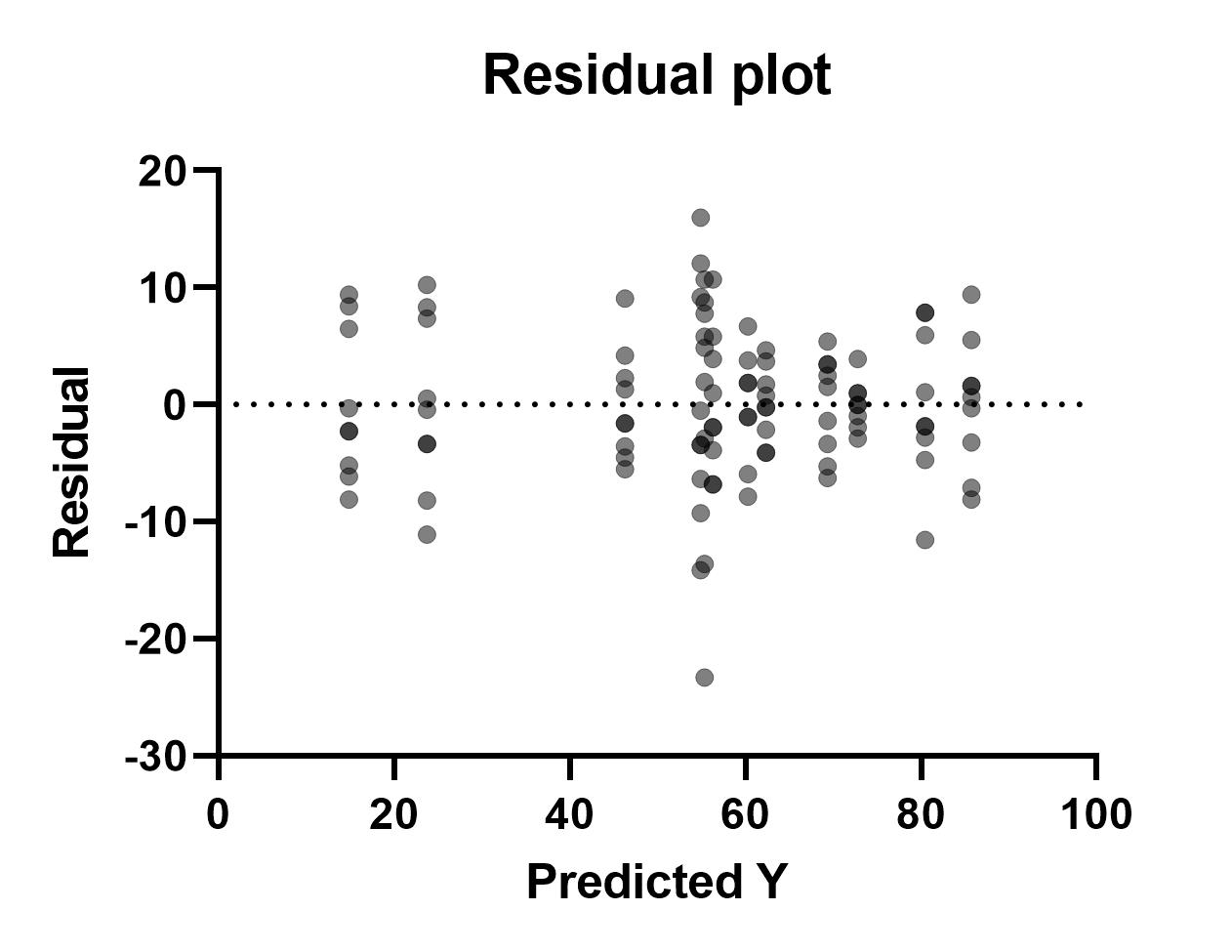
**

**Figure 15. Residual plot.**

**Table 14. Brown-Forsythe and Welch ANOVA tests.**

| Brown-Forsythe ANOVA test |  |
| --- | --- |
| F* (DFn, DFd) | 91.97 (11.00, 55.54) |
| P value | <0.0001 |
| P value summary | **** |
| Significant diff. among means (P < 0.05)? | Yes |
|  |  |
| Welch's ANOVA test |  |
| W (DFn, DFd) | 102.8 (11.00, 37.35) |
| P value | <0.0001 |
| P value summary | **** |
| Significant diff. among means (P < 0.05)? | Yes |

**Table 15. Dunnets’s T3 multiple comparisons test.**

| Dunnett's T3 multiple comparisons test | Mean Diff. | 95.00% CI of diff. | Summary | Adjusted P Value |
| --- | --- | --- | --- | --- |
| *B. pauloensis* vs. *B. mattogrossensis* | -5.286 | -16.66 to 6.084 | ns | 0.9380 |
| *B. pauloensis* vs. *B. alternatus* | 20.17 | 9.386 to 30.96 | *** | 0.0001 |
| *B. pauloensis* vs. *B. caribbaeus* | 25.57 | 8.844 to 42.29 | ** | 0.0014 |
| *B. pauloensis* vs. *B. lanceolatus* | 25.13 | 6.951 to 43.32 | ** | 0.0035 |
| *B. pauloensis* vs. *B. atrox* | 24.16 | 12.52 to 35.81 | **** | <0.0001 |
| *B. pauloensis* vs. *B. asper* | 11.11 | 0.6200 to 21.60 | * | 0.0325 |
| *B. pauloensis* vs. *B. leucurus* | 18.12 | 8.071 to 28.18 | *** | 0.0003 |
| *B. pauloensis* vs. *B. pictus* | 34.20 | 23.47 to 44.92 | **** | <0.0001 |
| *B. pauloensis* vs. *B. diporus* | 7.659 | -2.493 to 17.81 | ns | 0.2120 |
| *B. pauloensis* vs. *B. taeniatus* | 56.74 | 43.64 to 69.84 | **** | <0.0001 |
| *B. pauloensis* vs. *B. oligolepis* | 65.59 | 53.37 to 77.81 | **** | <0.0001 |
| *B. mattogrossensis* vs. *B. alternatus* | 25.46 | 15.70 to 35.22 | **** | <0.0001 |
| *B. mattogrossensis* vs. *B. caribbaeus* | 30.85 | 14.44 to 47.26 | *** | 0.0002 |
| *B. mattogrossensis* vs. *B. lanceolatus* | 30.42 | 12.48 to 48.36 | *** | 0.0006 |
| *B. mattogrossensis* vs. *B. atrox* | 29.45 | 18.60 to 40.30 | **** | <0.0001 |
| *B. mattogrossensis* vs. *B. asper* | 16.40 | 6.955 to 25.84 | *** | 0.0002 |
| *B. mattogrossensis* vs. *B. leucurus* | 23.41 | 14.40 to 32.42 | **** | <0.0001 |
| *B. mattogrossensis* vs. *B. pictus* | 39.48 | 29.75 to 49.21 | **** | <0.0001 |
| *B. mattogrossensis* vs. *B. diporus* | 12.94 | 4.237 to 21.65 | ** | 0.0029 |
| *B. mattogrossensis* vs. *B. taeniatus* | 62.03 | 49.49 to 74.57 | **** | <0.0001 |
| *B. mattogrossensis* vs. *B. oligolepis* | 70.87 | 59.40 to 82.35 | **** | <0.0001 |
| *B. alternatus* vs. *B. caribbaeus* | 5.394 | -10.69 to 21.48 | ns | 0.9941 |
| *B. alternatus* vs. *B. lanceolatus* | 4.962 | -12.75 to 22.67 | ns | 0.9996 |
| *B. alternatus* vs. *B. atrox* | 3.991 | -6.095 to 14.08 | ns | 0.9867 |
| *B. alternatus* vs. *B. asper* | -9.061 | -17.37 to -0.7571 | * | 0.0247 |
| *B. alternatus* vs. *B. leucurus* | -2.050 | -9.613 to 5.514 | ns | >0.9999 |
| *B. alternatus* vs. *B. pictus* | 14.02 | 5.280 to 22.77 | *** | 0.0005 |
| *B. alternatus* vs. *B. diporus* | -12.51 | -19.67 to -5.356 | *** | 0.0006 |
| *B. alternatus* vs. *B. taeniatus* | 36.57 | 24.48 to 48.66 | **** | <0.0001 |
| *B. alternatus* vs. *B. oligolepis* | 45.42 | 34.52 to 56.31 | **** | <0.0001 |
| *B. caribbaeus* vs. *B. lanceolatus* | -0.4315 | -20.89 to 20.03 | ns | >0.9999 |
| *B. caribbaeus* vs. *B. atrox* | -1.402 | -17.75 to 14.94 | ns | >0.9999 |
| *B. caribbaeus* vs. *B. asper* | -14.46 | -30.32 to 1.413 | ns | 0.0892 |
| *B. caribbaeus* vs. *B. leucurus* | -7.443 | -23.58 to 8.697 | ns | 0.8169 |
| *B. caribbaeus* vs. *B. pictus* | 8.630 | -7.510 to 24.77 | ns | 0.7104 |
| *B. caribbaeus* vs. *B. diporus* | -17.91 | -33.63 to -2.185 | * | 0.0224 |
| *B. caribbaeus* vs. *B. taeniatus* | 31.18 | 14.14 to 48.21 | *** | 0.0001 |
| *B. caribbaeus* vs. *B. oligolepis* | 40.02 | 23.46 to 56.58 | **** | <0.0001 |
| *B. lanceolatus* vs. *B. atrox* | -0.9709 | -19.11 to 17.17 | ns | >0.9999 |
| *B. lanceolatus* vs. *B. asper* | -14.02 | -31.95 to 3.899 | ns | 0.1910 |
| *B. lanceolatus* vs. *B. leucurus* | -7.012 | -24.94 to 10.91 | ns | 0.9387 |
| *B. lanceolatus* vs. *B. pictus* | 9.061 | -8.697 to 26.82 | ns | 0.7698 |
| *B. lanceolatus* vs. *B. diporus* | -17.48 | -35.65 to 0.6939 | ns | 0.0617 |
| *B. lanceolatus* vs. *B. taeniatus* | 31.61 | 12.98 to 50.24 | *** | 0.0004 |
| *B. lanceolatus* vs. *B. oligolepis* | 40.45 | 22.20 to 58.71 | **** | <0.0001 |
| *B. atrox* vs. *B. asper* | -13.05 | -22.95 to -3.153 | ** | 0.0050 |
| *B. atrox* vs. *B. leucurus* | -6.041 | -15.43 to 3.352 | ns | 0.4662 |
| *B. atrox* vs. *B. pictus* | 10.03 | -0.1288 to 20.19 | ns | 0.0550 |
| *B. atrox* vs. *B. diporus* | -16.50 | -25.64 to -7.369 | *** | 0.0006 |
| *B. atrox* vs. *B. taeniatus* | 32.58 | 19.78 to 45.37 | **** | <0.0001 |
| *B. atrox* vs. *B. oligolepis* | 41.42 | 29.68 to 53.17 | **** | <0.0001 |
| *B. asper* vs. *B. leucurus* | 7.012 | -0.03436 to 14.06 | ns | 0.0519 |
| *B. asper* vs. *B. pictus* | 23.09 | 14.69 to 31.48 | **** | <0.0001 |
| *B. asper* vs. *B. diporus* | -3.452 | -10.11 to 3.202 | ns | 0.7495 |
| *B. asper* vs. *B. taeniatus* | 45.63 | 33.81 to 57.45 | **** | <0.0001 |
| *B. asper* vs. *B. oligolepis* | 54.48 | 43.87 to 65.08 | **** | <0.0001 |
| *B. leucurus* vs. *B. pictus* | 16.07 | 8.407 to 23.74 | **** | <0.0001 |
| *B. leucurus* vs. *B. diporus* | -10.46 | -15.53 to -5.398 | **** | <0.0001 |
| *B. leucurus* vs. *B. taeniatus* | 38.62 | 27.06 to 50.18 | **** | <0.0001 |
| *B. leucurus* vs. *B. oligolepis* | 47.46 | 37.08 to 57.85 | **** | <0.0001 |
| *B. pictus* vs. *B. diporus* | -26.54 | -33.81 to -19.26 | **** | <0.0001 |
| *B. pictus* vs. *B. taeniatus* | 22.55 | 10.39 to 34.71 | *** | 0.0002 |
| *B. pictus* vs. *B. oligolepis* | 31.39 | 20.56 to 42.23 | **** | <0.0001 |
| *B. diporus* vs. *B. taeniatus* | 49.08 | 37.48 to 60.69 | **** | <0.0001 |
| *B. diporus* vs. *B. oligolepis* | 57.93 | 47.63 to 68.23 | **** | <0.0001 |
| *B. taeniatus* vs. *B. oligolepis* | 8.846 | -4.344 to 22.04 | ns | 0.4704 |
