# Supplementary material for: Bothrops venom variation drives niche-specific pharmacology through Ca2+ signalling and membrane damage
Source: Front Pharmacol. 2026 Mar 31;17:1769550. doi: 10.3389/fphar.2026.1769550 (PMC13076241; doi:10.3389/fphar.2026.1769550)
Supplement: Supplementary file 3 [file Supplementaryfile2.docx]

**Supplementary Material 2**


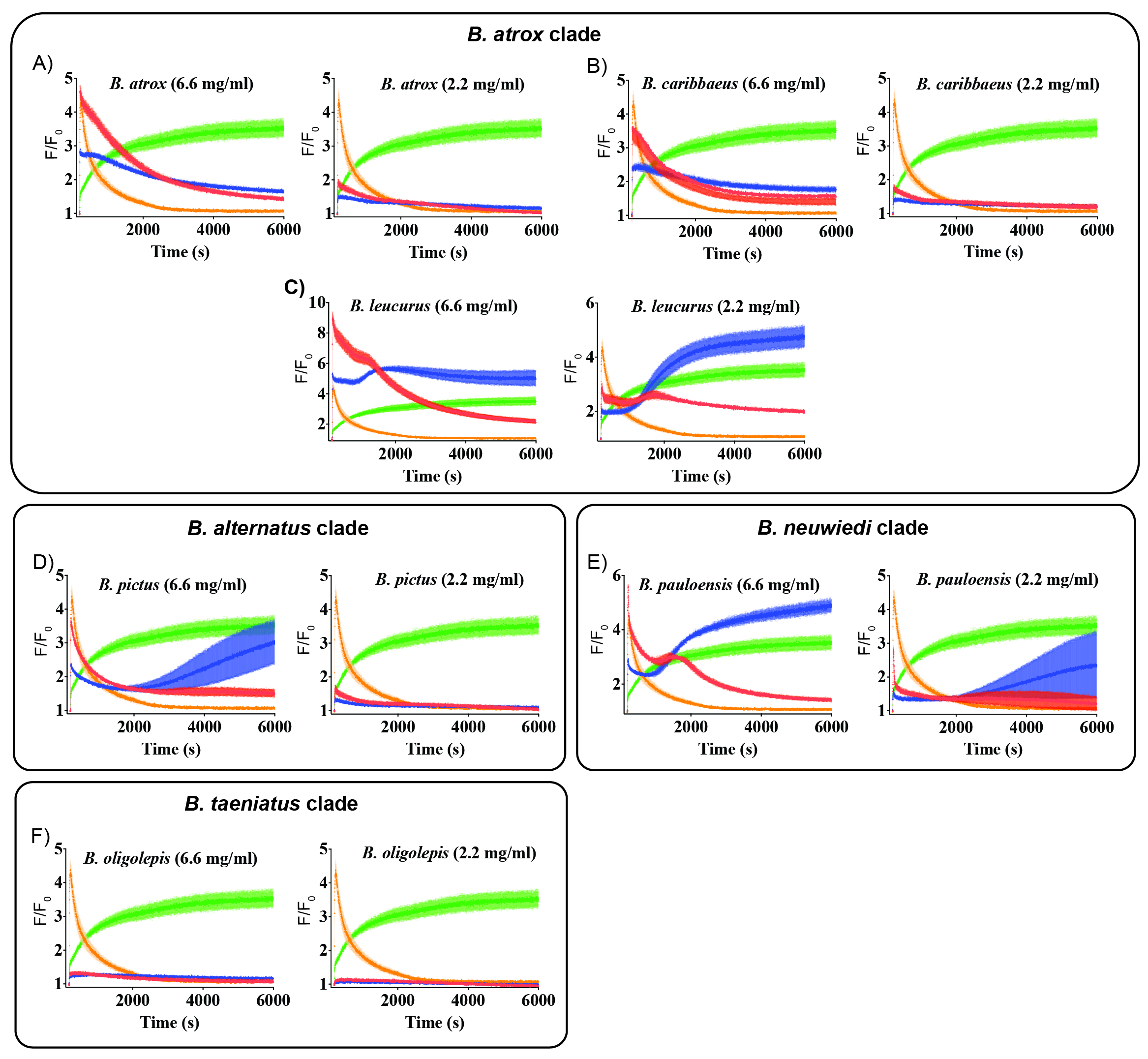


**Supplementary Material 2.1.** Fluorescence traces of propidium iodide (DNA response, blue traces) and Ca 4 dye ([Ca^2+^]_i_ response, red traces) in SHSY5Y cells exposed to venom from representative species of the clades *B. atrox* (A-C), *B. alternatus* (D), *B. neuwiedi*  (E), and *B. taeniatus* (F). All graphs include the positive control: fluorescence traces of cells exposed to melittin_._ Data are represented by mean ± SEM of F/F_0_ recorded for approximately 6000 s (n = 9 from 3 independent experiments performed in triplicates).

**
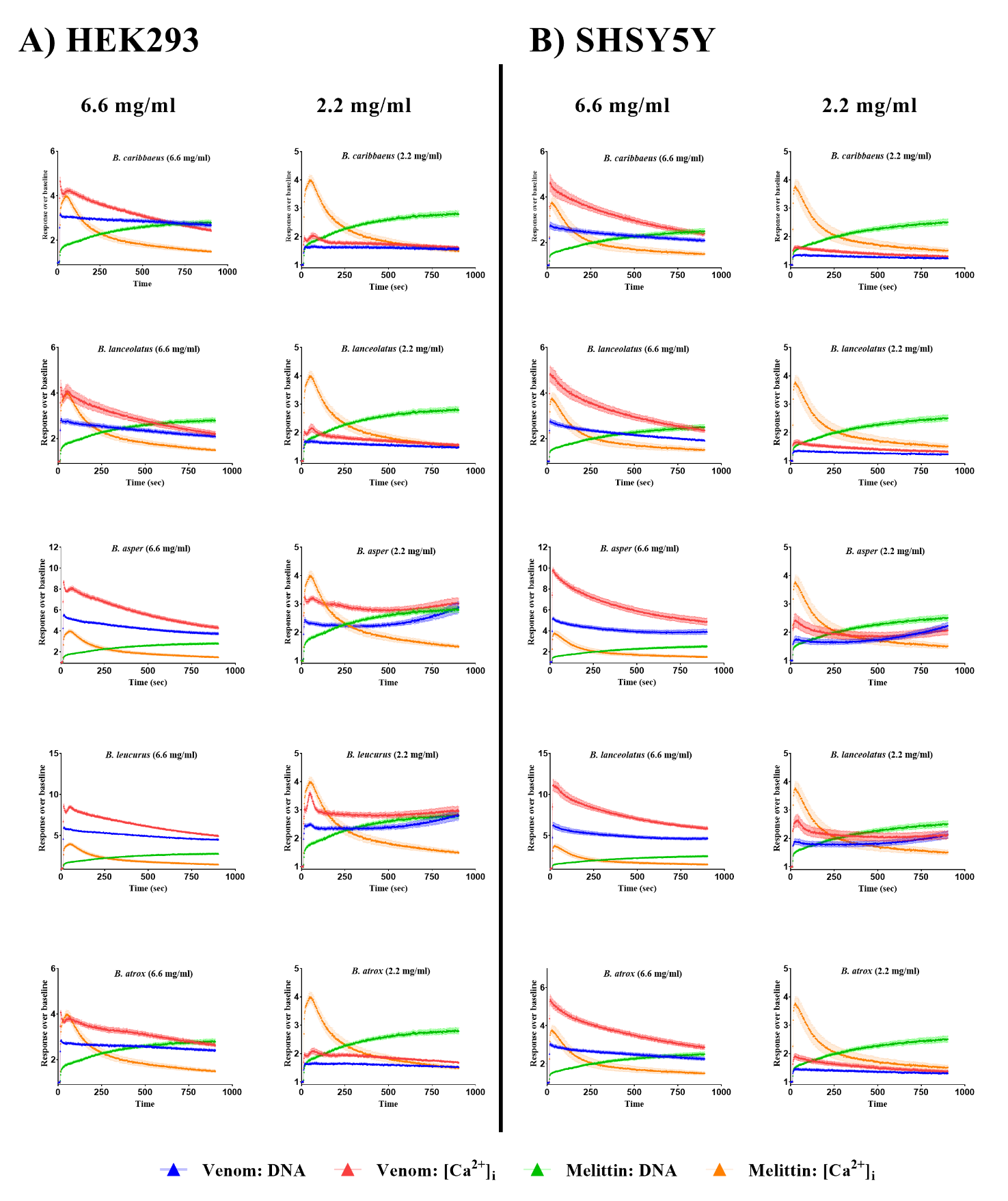
**

**Supplementary Material 2.2.** Fluorescence traces of propidium iodide (DNA exposure, blue traces) and Ca 4 dye ([Ca^2+^]_i_ response, red traces) in HEK293 and SHSY5Y cells exposed to venom from five *Bothrops* species in the *B. atrox* clade. All graphs include the positive control: fluorescence traces of cells exposed to melittin. Data are represented by mean ± SEM of F/F_0_ recorded for approximately 800 s (n = 9 from 3 independent experiments performed in triplicates).

**
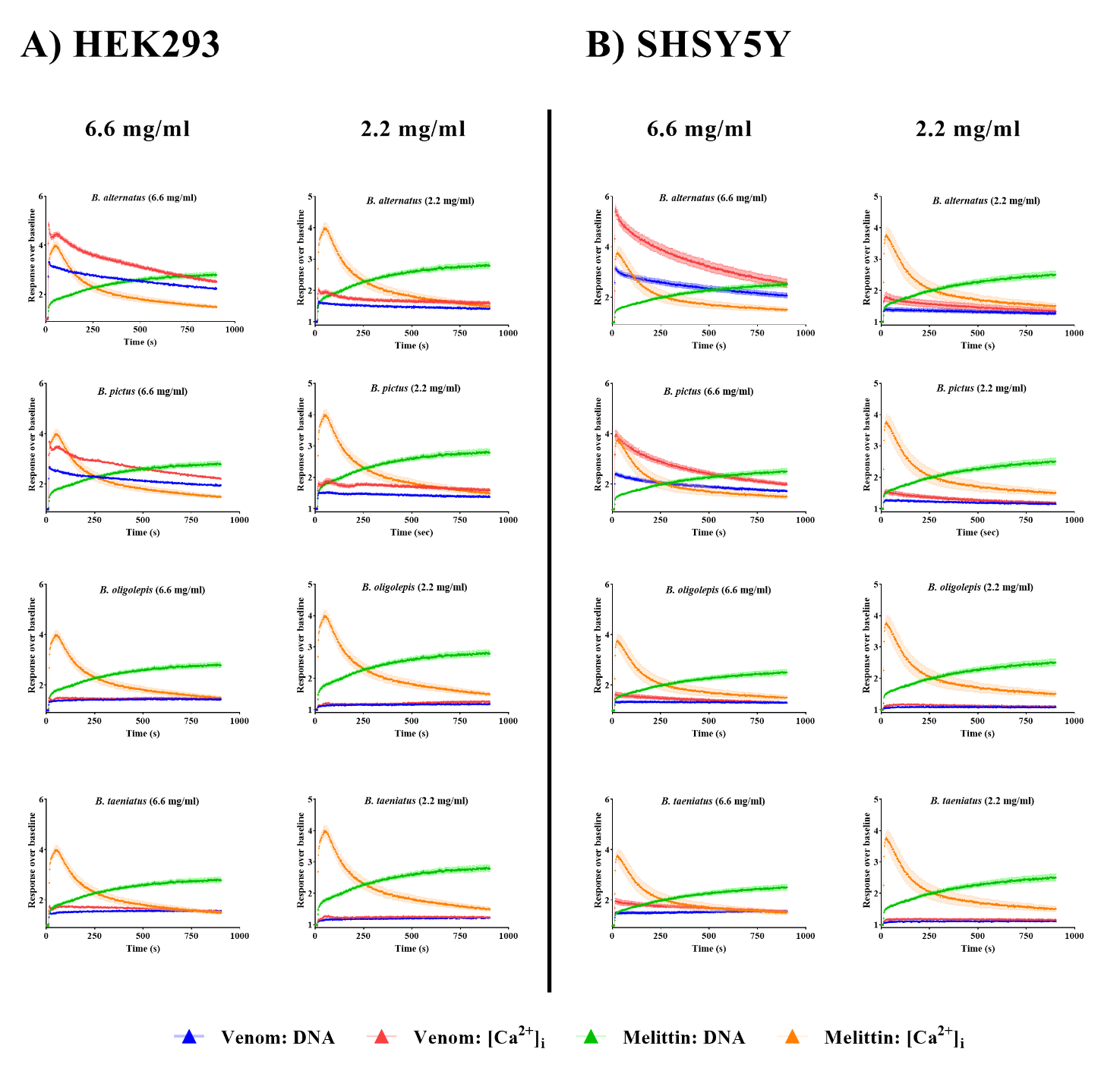
**

**Supplementary Material 2.3.** Fluorescence traces of propidium iodide (DNA exposure, blue traces) and Ca 4 dye ([Ca^2+^]_i_ response, red traces) in HEK293 and SHSY5Y cells exposed to venom from two *Bothrops* species in the *B. alternatus* clade and two *Bothrops* species in the *B. taeniatus* clade. All graphs include the positive control: fluorescence traces of cells exposed to melittin. Response over baseline is equivalent to F/F_0_. Data are represented by mean ± SEM of F/F_0_ recorded for approximately 800 s (n = 9 from 3 independent experiments performed in triplicates).

**
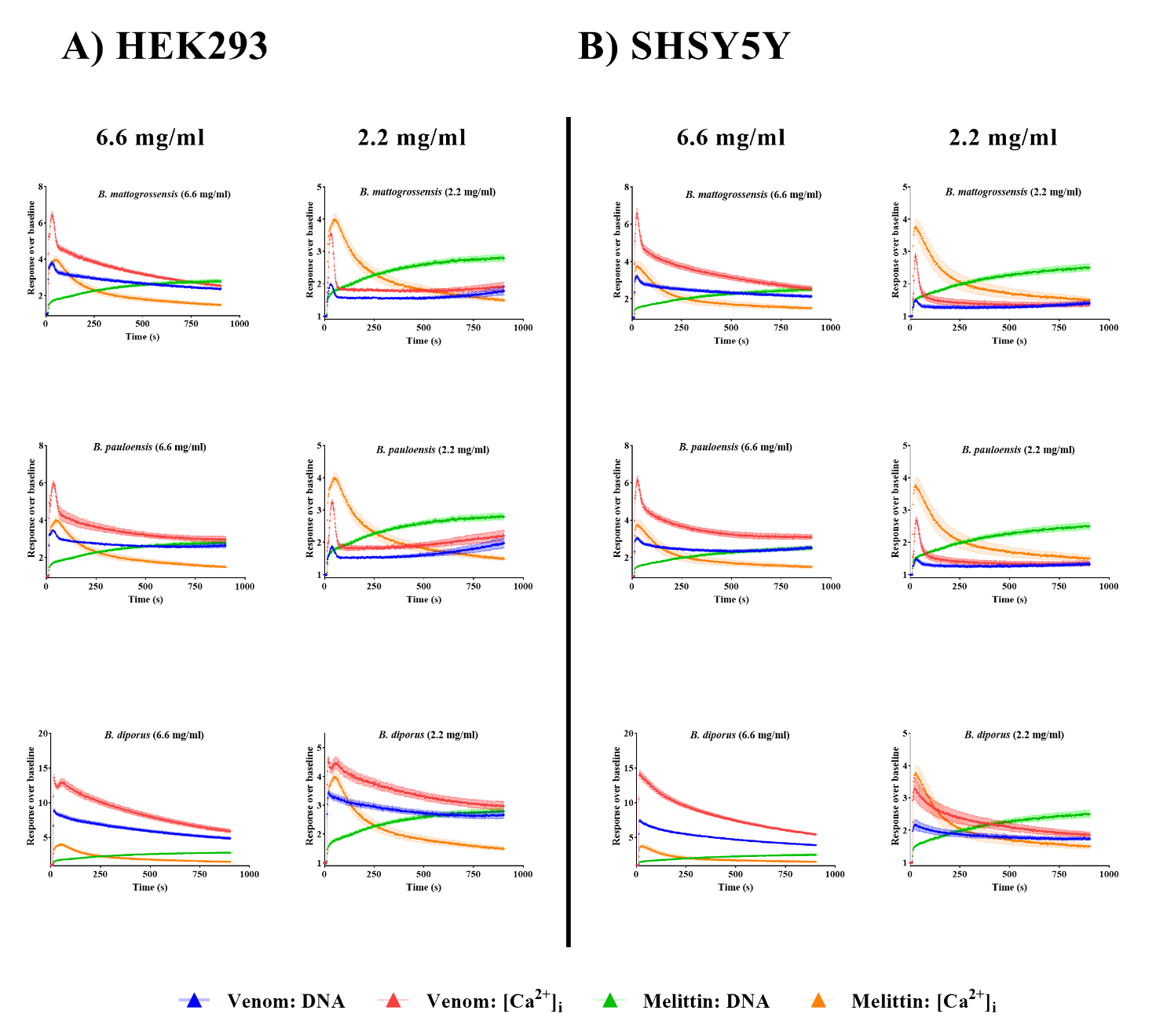
**

**Supplementary Material 2.4.** Fluorescence traces of propidium iodide (DNA exposure, blue traces) and Ca 4 dye ([Ca^2+^]_i_ response, red traces) in HEK293 and SHSY5Y cells exposed to venom from three *Bothrops* species in the *B. neuwiedi* clade. All graphs include the positive control: fluorescence traces of cells exposed to melittin. Data are represented by mean ± SEM of F/F_0_ recorded for approximately 800 s (n = 9 from 3 independent experiments performed in triplicates).
