# Supplementary material for: Bothrops venom variation drives niche-specific pharmacology through Ca2+ signalling and membrane damage
Source: Front Pharmacol. 2026 Mar 31;17:1769550. doi: 10.3389/fphar.2026.1769550 (PMC13076241; doi:10.3389/fphar.2026.1769550)
Supplement: Supplementary file 4 [file Supplementaryfile4.docx]

**
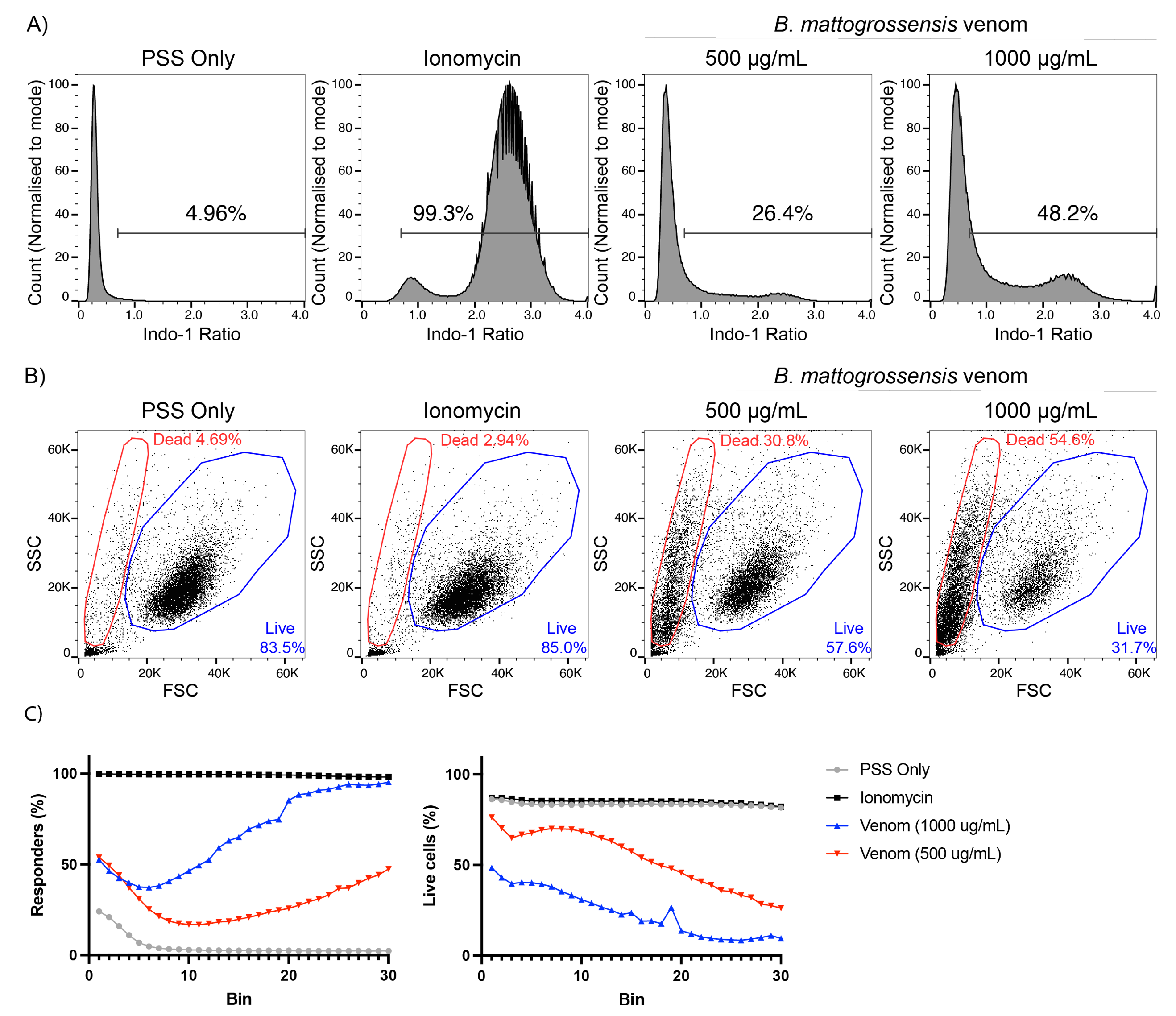
Supplementary Material 4**

**Supplementary Material 4.** (A-C) Flow cytometry data for HEK293T cells loaded with 5 μM Indo-1 and resuspended in either PSS only, Ionomycin (10 μM), or B. mattogrossensis venom at 500 μg/mL or 1000 μg/mL, immediately prior to analysis for 5 min. Gate for responding cells was set based on top ~5% of cells by Indo-1 ratio in ‘PSS Only’ control sample. (A) Histograms of Indo-1 ratio (Fluorescence intensity at 460 nm / 379 nm). Counts are normalised to mode for all samples. (B) FSC vs. SSC dot plots for samples analysed by flow cytometry. 10,000 events are displayed and are representative of the total events run over 5 minutes of recording. (C) Proportion of responder cells and live cells across time bins for each sample. Each bin is representative of 10 s of recording.
